# Influence of Halogen and Its Position on Crystal Packing: Proposals for Molecular-Level Crystallization Mechanisms of Isomeric Halogenated Benzoximes

**DOI:** 10.1021/acsomega.5c08976

**Published:** 2025-12-16

**Authors:** Patrick Teixeira Campos, Isabella Burchardt Ferreira, Pedro Henrique Cunha do Couto, Davi Fernando Back

**Affiliations:** † Laboratório de Química Orgânica Sintética, Estrutural e Computacional (LaQuiOSEC), Instituto Federal de Educação, Ciência e Tecnologia Sul-rio-grandense (IFSul) − Câmpus Pelotas, Pelotas 96015-360, Brazil; ‡ Laboratório de Materiais Inorgânicos (LMI), Departamento de Química − UFSM, Av. Roraima 1000, Santa Maria, RS 97105-900, Brazil

## Abstract

A thorough understanding of the crystallization process is crucial for several fields that depend on meticulous control of the formation of crystalline solids. Therefore, the elucidation of crystallization mechanisms at the molecular level is fundamental to understanding the influence of interactions on the formation of organized solid structures. We propose a crystallization mechanism for isomeric halogenated benzoximes with distinct crystal morphologies based on nonclassical nucleation theory. The results were supported by computational tools that calculate theoretical energetic and topological data for intermolecular and supramolecular interactions. The interactions were verified and energetically classified by density functional theory (DFT) and their contribution per contact point was analyzed using Quantum Theory of Atoms in Molecules (QTAIM) topological Analysis. The crystallization mechanisms were proposed based on the model developed by our research group, in which the strongest interactions of the first coordination sphere (O–H···N or π···π) form one-dimensional (1D) supramolecular chains. From this chain, hypotheses of approximation between chains were analyzed to determine the most stable arrangement for the formation of the two-dimensional supramolecular layer (2D, mainly C–H···H–C, C–H···X, and C–H···O). Following this same evaluation, it was determined that three-dimensional (3D) growth is completed by the X···X (halogen··halogen) and C···X interactions. This work studies the influence of different halogen substituents, in addition to the influence of their positions in the benzene ring, and investigates the possibility of isostructurality and the crystallization mechanism of isomeric benzoximes, expanding the understanding of how small structural variations impact crystal packing.

## Graphical abstract

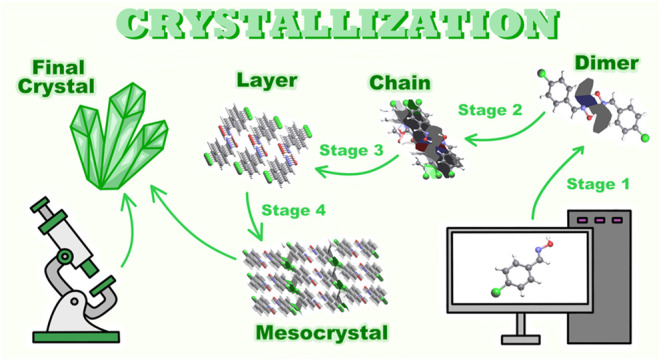

## 1 Introduction

The use of oximes is widely described in the literature, mainly in the medical field. This class of compounds is widely used in the treatment of people exposed to organophosphate substances.^1,2^ In this same area, some recent studies have shown that oximes have anti-inflammatory^3^ and anticancer activity.^3,4^ In addition to applications in the medical field, oximes can also be used to replace some pesticides. Replacing, for example, chemical nematicides, which control plant parasites, but are harmful to the environment and human health.^5^ The diversity of applications of oximes is based on the different properties that each molecular structure presents,^6^ due to the intermolecular interactions present in the compound, and the presence of different acceptors and donors can favor the formation of different structures with different applications.^7^ Studies on intermolecular interactions, as well as studies in the field of supramolecular chemistry (chemistry beyond the molecule)^8^ and crystal engineering (functional crystalline solids)^9^ are extremely important in understanding the relationship between structure and property. These studies, mainly on compounds containing the aryl halide fragment (*para*-substituted),^10^ have revealed remarkable plastic and elastic mechanical properties.^11^ These properties enable the development of devices used in biomedicine^12^ and mechano-pharmaceuticals,^13^ as well as other applications in optical devices,^14^ and mechanosensors,^15^ for example.

Decades of work have examined how crystals form.^16,17^ Although there is no consensus on how this process occurs, various theories have different approaches to the formation of crystal structures at the molecular level. The most discussed frameworks are the classical nucleation theory,^18,19^ the prenucleation theory^19^ and the nonclassical (two-step) theory.^20,21^ The classical theory treats nucleation as the barriered formation of a crystalline cluster from monomers of the crystallization process, as it suggests the approximation of monomers to critical agglomerates with crystalline characteristics, forming a nucleus which then gives rise to the crystal.^22,23^ The nonclassical theories, whether prenucleation or two-step, suggest the formation of disordered, nonsolid intermediate structures that approach each other, forming, over time, a liquid agglomerate, which is still unstable, and then this agglomerate undergoes an ordering process, giving rise to the solid crystal.^21,23,24^ Given the difficulties in ensuring that all important crystal forms have been obtained experimentally, researchers in this field have long sought the ability to predict crystal polymorphs theoretically.^25^ Computational analysis of intermolecular interactions^26,27^ (DFT/QTAIM)^26–28,30^ helps rationalize how topological evaluation^26^ and noncovalent bonds affect the crystallization process.^25,28^ Furthermore, these tools can also be used to predict electrical and electronic properties.^28,29^ Comparing experimental data recently obtained by Biran et al.^31^ and Elizebath et al.,^32^ it is possible to observe that the computational data presented in this work (stabilization energies that are calculated to hierarchically order supramolecular growths guided by self-assembly) are in agreement with the results obtained by them (reports of crystal growth by self-assembly). Under thermodynamic control, crystal formation follows the lowest-free-energy pathway. To determine the possible mechanisms for assembly, computer simulations are used to compute the energy released by intermolecular interactions.^33,34^

Previous computational studies^35–41^ have proposed molecular-level crystallization mechanisms based solely on the energies of intermolecular interactions within the first coordination sphere. We have previously employed the same approach and applied it to halogenated benzoic acids,^42^ phenols^43^ and benzamides.^44^ Due to observed factors that were neglected in the previous approach (only computational calculus were made with supramolecular dimers), a new model was created by our research group and applied to both halogenated anilines,^44^ and halogenated benzyl alcohols.^45^ The model consists of energetic and topological analyses to sequentially order the approximations of intermediate supramolecular structures (whether chains or layers). This approach can determine the energy of this approximation using the concepts of compact packing (contact area between supramolecular structures) in the crystal structure of the compounds. In addition to highlighting the interactions that form supramolecular chains (1D) and how approaching chains form supramolecular layers (2D), the model also suggests that there is the formation of a mesocrystal (3D) starting from the approximation of layers. Another advantage in this model is that before the 3D growth is completed, it investigates whether growth can occur in a direction already observed at some previous stage of the mechanism.^44^

Therefore, the objective of this work is to apply our model to halogenated benzoximes to develop proposals for crystallization mechanisms at the molecular level. By applying the model to several halogenated benzoximes, especially the isomeric ones, it is possible to analyze the influence of the different halogens present on the benzene ring, as well as the influence of their positions (ortho, meta, or para), and how these differences in molecular structure affect the proposed mechanisms. Furthermore, we aimed to investigate the possibility of isostructurality in benzoximes substituted with halogens in the same position. We also sought to investigate whether the orientation of the proposed supramolecular growths is directly related to the unit cell parameters, which normally reflect the crystal habit.

## 2 Results and Discussion

For the development of this study, the class of compounds chosen was halogenated benzoximes (Figure 1). *Ortho*-, *meta*-, and *para*-substituted halogenated benzoximes, as well as geometric isomers, were used to investigate how these characteristics impact the proposed crystallization mechanisms. The compounds studied are identified by the CCDC code and the abbreviation used, and both pieces of information are listed in the table in Figure 1.

**Figure 1:**
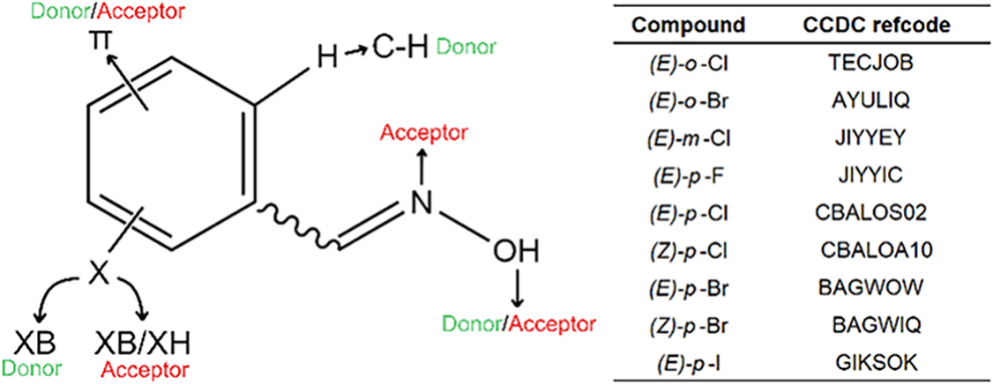
Structures, codes, and possible sites of intermolecular interactions of halogenated oximes present in CCDC.

The crystal structures used for this work have already been reported in the literature and were introduced at different times (*(E)-p*-Cl,^46^ *(Z)-p*-Cl,^47,48^ *(E)-o*-Cl,^49,50^ *(E)-p*-Br,^50,51^ *(Z)-p*-Br,^52,53^ *(E)-*o-Br,^54^ *(E)-m*-Cl,^55^ *(E)-p*-F,^55^ and *(E)-p*-I^7^). The study began by obtaining. CIF files from the CSD, and the first step was to determine the first coordination spheres (clusters) using the Voronoi-Dirichlet Polyhedron (VDP) method.^56^ Figures representing the clusters are available in the Supporting Information (Figure S1).

The analysis performed on the clusters begins with determination of the molecular coordination number (MCN). These values were obtained by using the VDP method. With the exception of the *(E)-p*-I oxime cluster (which had an MCN of 13) because iodine is a large atom, and this can cause the MCN to be smaller, the other clusters studied had an MCN of 14. The clusters were then divided into principal and secondary planes to facilitate visualization of the interactions present. The principal plane was defined as the plane parallel to the central molecule, and the secondary plane was subdivided into upper and lower planes, as shown in Figure 2. The oxime *(E)-p*-Cl was used to represent the division of the planes (**2a**—principal plane, **2b**—secondary plane). The planes for the other compounds are shown in the Supporting Information, in Figures S3–S9.

**Figure 2:**
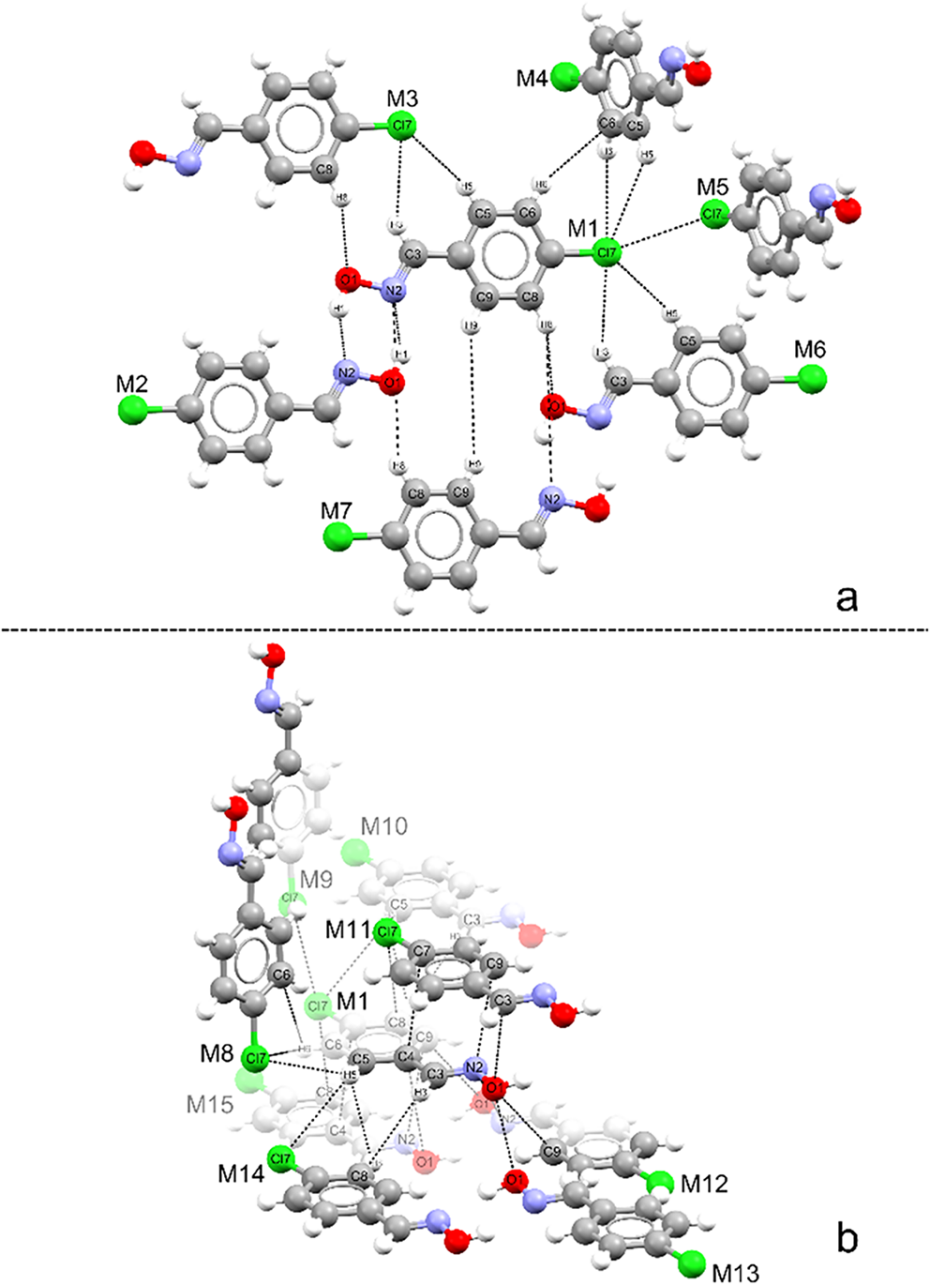
Intermolecular interactions between the central molecule and neighboring molecules in the first coordination sphere of compound *(E)-p*-Cl: (a) region adjacent to the principal plane and (b) upper/lower region.

The clusters were isolated in pairs, named M1···Mn, where M1 is the central molecule and Mn represents the peripheral molecules up to the MCN of the compound. Figure 3 shows some characteristic interactions of the compounds with their contact areas, namely O–H···N (Figure 3a), π···π (Figure 3b), C–H···X (Figure 3c), and X···X (Figure 3d), where X is the halogen atom. The energies of the pairs of molecules were calculated using a quantitative parameter calculated by DFT (Density Functional Theory),^57–59^ at the ωB97X-D3 ^60^ level of theory and the set of bases def2-tzvp.^61^

**Figure 3:**
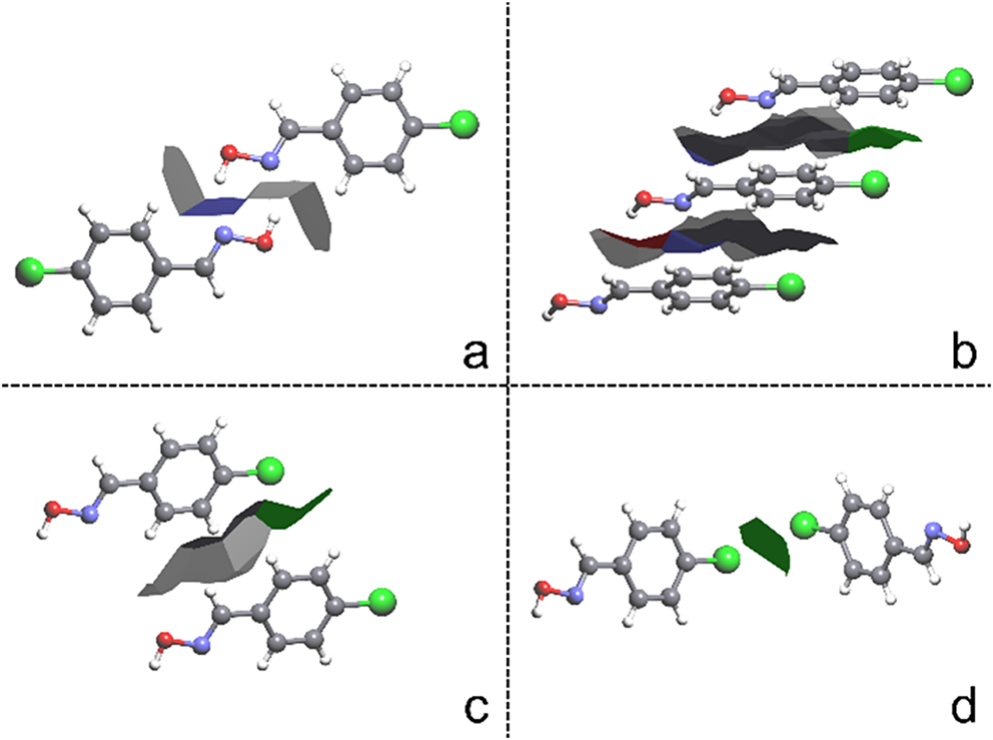
Areas of each contact; (a) O–H···N dimer, present in all compounds. (b) π stacking, vertical alignment of molecules, where mainly C···C interactions occur. (c) C–H···Cl interaction. (d) Cl···Cl interaction.

This approach was used to determine the energy arising from the intermolecular interactions between the pairs of molecules. The choice of this level of theory is due to the dispersion correction that the method applies, which is essential for calculations of weak interactions involving C–H groups.^60^ The choice of the basis set is due to it being a robust basis to the point of allowing calculations with heavy atoms, including iodine.^61^ QTAIM (Quantum Theory of Atoms in Molecules) analysis was used to determine the contact points between the molecules as well as the energy contribution of each one, as illustrated in Figure 4a with the M1···M8 interaction of the oxime *(E)-p*-Cl. When a bonding critical point (BCP)^62,63^ appears between the atoms of each molecule, it is in this region that the electron density gradient is canceled out and, therefore, the orange path, shown in Figure 4b, is the path in which there is the greatest variation in electron density, which describes the existence of a contact point (interaction) between the two molecules.

**Figure 4:**
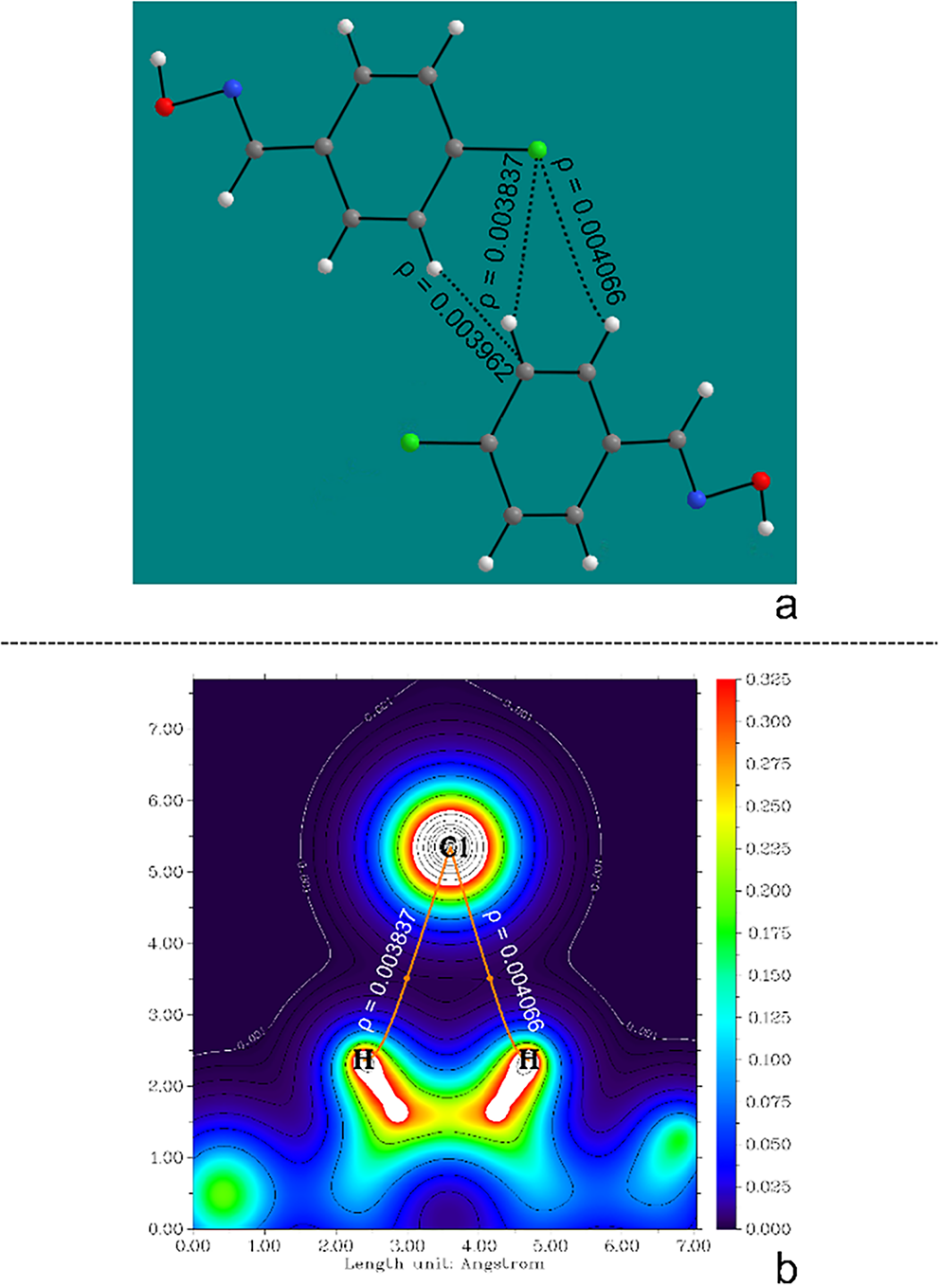
Pair of molecules M1···M8 of the compound (*E*)-*p*-Cl (a) used to demonstrate the obtaining of ρ and (b) used to construct the electron density image of two contact points between the molecules.

In this way, two distinct C–H···Cl intermolecular interactions between the molecules were determined. The energy of the contact points was calculated from a proportional relationship using the electron density (ρ) of each contact and comparing it to the total energy of the interaction between each pair of molecules. The equation used for this contribution is illustrated in eq 1, then Table 1 represents how this calculation is done.

1 $${c\mathrm{ontact}\;\mathrm{energy}}_{1}=\frac{\rho_{1}{\;}^{*}i\mathrm{nteraction}\;\mathrm{energy}}{\sum \rho }$$

**Table 1:** Example of Determination of the Contribution of Interaction, in Terms of Electron Density (ρ), Percentage, and Contact Energy^a^

**interaction energy** (kcal/mol)	**ρ**	**%**	**contact energy** (kcal/mol)
**–2.79**	0.003962	33	–0.93
	0.003837	32	–0.90
	0.004066	34	–0.96
	**∑0.011865**	**100**	**–2.79**

a Pair of molecules M1···M8 of compound (*E*)-*p*-Cl.

In continuation of the supramolecular study of halogenated benzoximes, compound *(E)-p*-Cl was used as an example. In the pair M1···M8, it was observed that the total interaction energy is −2.79 kcal·mol^–1^ and the contact area is 15.96 Å^2^. In this pair of molecules, there is a C–H···C interaction and two C–H···Cl contact points. However, even if the two contact points are of the same classification (C–H···Cl), the energy contribution of each contact varies because the interatomic distance is different, presenting a variation of less than 0.1 Å between each measurement (3.170 and 3.212) and, consequently, one of the electron densities will also be greater than the other (Figure 4b). The smallest ρ is 0.003837, and the largest is 0.004066; with this, it is possible to calculate the energy contribution proportion and obtain the energy of each contact point. The contact energy of the shortest distance is the one with the largest ρ, being −0.96 kcal mol^–1^, and the longest distance, therefore, the one with the smallest ρ, has a contact energy of −0.90 kcal mol^–1^. The remainder of the stabilization energy refers to the C–H···C contact point, which has an energy of −0.93 kcal·mol^–1^ and the percentage contribution of the data presented is, respectively, 34, 32 and 33%. Table 2 presents data on the contact area, interaction energy, interatomic distance, and electron density for all pairs of molecules in the benzoxime (*E*)-*p*-Cl cluster. This evaluation was performed for all compounds, the tables for which can be found in the Supporting Information (Tables S1–S8).

**Table 2:** Contact Area, BSSE Corrected Interaction Energy, Interaction, Interatomic Distance, Contact Energy, Contribution Percentage, and Electron Density of M1···Mn for the Supramolecular Cluster of *(E)-p*-Cl

dimer	contact area^a^ (Å^2^)	interaction energy^b^ (kcal·mol^–1^)	interaction^c^	interatomic distance (Å)	contact energy^d^ (kcal·mol^–1^)	contribution percentage (%)^e^	**ρ**_INT_ ^c^ (u.a.)
M1···M2	22.39	–10.64	N2···H1–O1	1.952	–5.32	50	0.028964
			O1–H1···N2	1.952	–5.32	50	0.029004
M1···M3	17.85	–3.01	O1···H8–C8	2.581	–1.36	45	0.006848
			C3–H3···Cl7	3.083	–0.92	30	0.004608
			C5–H5···Cl7	3.121	–0.73	24	0.003665
M1···M4	15.96	–2.79	C6–H6···C6	2.970	–0.93	33	0.003969
			Cl7···H6–C6	3.212	–0.90	32	0.003837
			Cl7···H5–C5	3.170	–0.95	34	0.004065
M1···M5	3.92	–0.71	Cl7···Cl7	3.576	–0.71	100	0.005352
M1···M6	17.85	–3.01	C8–H8···O1	2.581	–1.36	45	0.006848
			Cl7···H3–C3	3.083	–0.92	30	0.004608
			Cl7···H5–C5	3.121	–0.73	24	0.003665
M1···M7	1.93	–0.48	N2···H8–C8	5.086	–0.13	26	0.000045
			C9–H9···H9–C9	4.046	–0.23	47	0.000081
			C8–H8···N2	5.086	–0.13	26	0.000045
M1···M8	15.96	–2.79	C5–H5···Cl7	3.170	–0.96	34	0.004066
			C6–H6···Cl7	3.212	–0.90	32	0.003837
			C6···H6–C6	2.970	–0.93	33	0.003962
M1···M9	3.92	–0.71	Cl7···Cl7	3.576	–0.71	100	0.005352
M1···M10	19.36	–2.98	Cl7···H5–C5	3.363	–0.96	32	0.003026
			C8–H8···H5–C5	2.772	–1.08	36	0.003399
			C8–H8···H3–C3	2.833	–0.95	32	0.002991
M1···M11	23.65	–6.05	C8···Cl7	3.751	–1.58	26	0.004252
			C4···C7	3.614	–1.38	23	0.003728
			N2···C9	3.457	–1.69	28	0.004566
			O1···C4	3.484	–1.40	23	0.003787
M1···M12	19.09	–3.93	C9···O1	3.412	–1.55	39	0.004286
			N2···N2	3.772	–0.83	21	0.002284
			O1···C9	3.412	–1.55	39	0.004283
M1···M13	4.40	0.07	O1···O1	3.345	0.07	100	0.003138
M1···M14	19.36	–2.98	C5–H5···Cl7	3.363	–0.96	32	0.003026
			C5–H5···H8–C8	2.772	–1.08	36	0.003399
			C3–H3···C8	3.183	–0.95	32	0.002991
M1···M15	23.65	–6.05	C4···O1	3.485	–1.40	23	0.003787
			C9···N2	3.457	–1.69	28	0.004568

a ToposPro.

b (GM1···Mn = EM1···Mn – 2*EM1) corrected by BSSE.

c QTAIM (MultiWFN).

d Ec = GCM1···Mn * Contribution percentage.

e heuristic values.

An analysis of the first coordination sphere was performed, and the energetic contribution of each interaction present in the cluster was verified for all compounds (Figure S1). For example, the O–H···N and C–H···X contact points, which are both present in all oximes. The number of O–H···N interactions does not vary between the compounds, always being 2 interactions. However, in relation to the percentage of energy that this interaction presents in relation to the total energy, there is a variation between 17.3 and 29.8% (Figure 5). The C–H···X interactions showed the highest number of occurrences, although their amount of stabilizing energy was not the highest in all compounds (Figure 5). When analyzing the first coordination sphere (Figure S1), it is found that the compounds *(E)-p*-Cl and *(E)-p*-Br are isostructural so that the number of occurrences and the energetic contributions of the interactions of these compounds are comparable. Interactions of the type C–H···X, C–H···O, X···X and C–H···C, for example, have the same occurrences and practically the same percentages of energy contribution. Another analysis of the interactions in the compound clusters is the occurrence of the O···X interaction, via short contact halogen bond.^64^ In compound *(E)-o*-Cl, it occurs twice and represents only 0.8% of the total energy of this compound. However, when this interaction occurs in *(E)-p*-I, there are five occurrences, and the contribution percentage is 15.7%, due to iodine having a larger radius. These various possibilities result in compounds exhibiting different crystallization mechanisms. The number of occurrences and energy contribution of each class of intermolecular interactions for each compound are presented in Figure 5. Figure S11 shows the number of occurrences and energy contribution of each class of intermolecular interactions considering all compounds.

**Figure 5:**
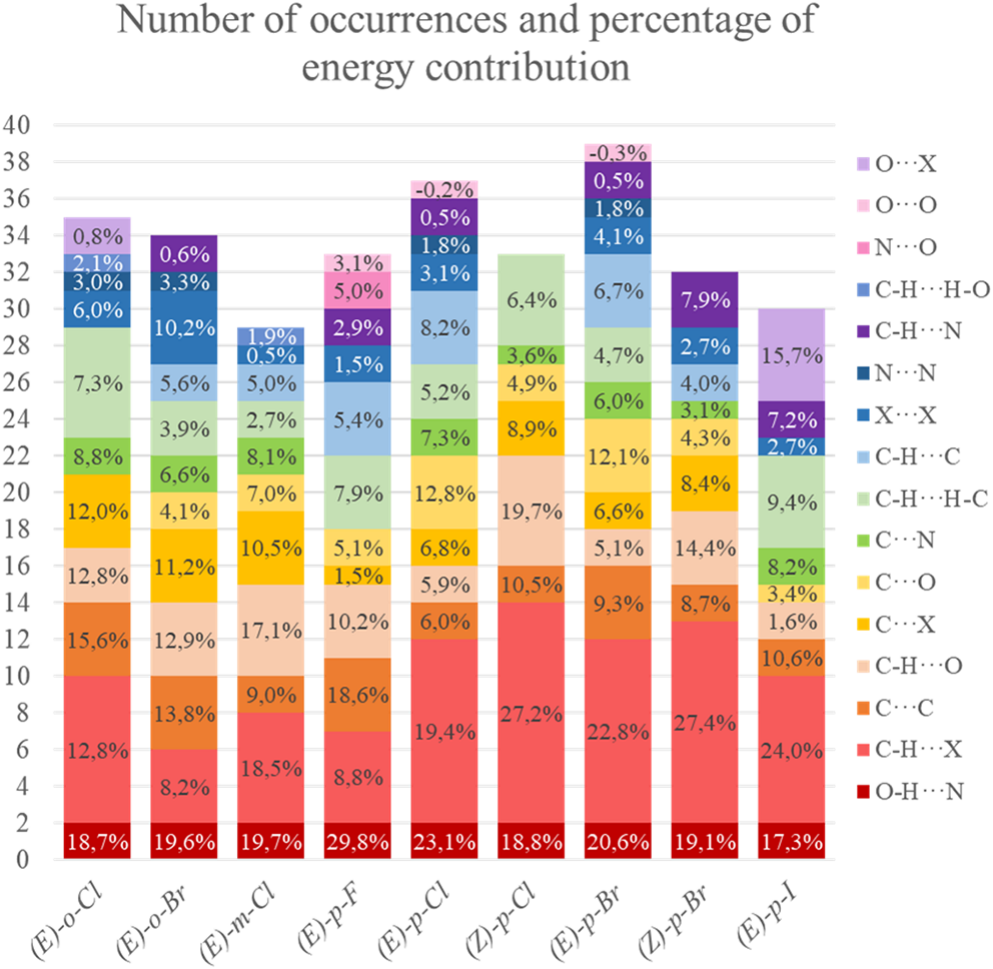
Number of occurrences and energetic contribution of each class of intermolecular interactions of each compound.

The following evaluation is a comparison of theoretical and experimental data. This evaluation is crucial to determine whether the energy data are reliable and can be applied to the model for developing the proposed crystallization mechanisms. The cohesive energy of the first coordination sphere resulting from the distinct intermolecular interactions of the molecular pairs is the theoretical part of the comparison (Table S9). The melting points (MP) of the studied compounds are experimental data and demonstrate the precise temperature at which a substance changes from the solid to the liquid state based on the energy supplied to break the intermolecular interactions. The values of these temperatures, in °C, were obtained from the references in Table S9. Compounds with similar characteristics were analyzed to see whether there was a good correlation between theoretical data (cohesive energy of the first coordination sphere) and experimental data (melting points). This analysis is presented in the supporting material (Figure S13). In summary, it was possible to verify that there is a directly proportional trend between the cohesive energy of the first coordination sphere and melting point for almost all compounds when evaluating different halogens in the same position or the same halogen in different positions.

This model^44^ advocates crystal growth guided by a hierarchy of the most stabilizing intermolecular interactions (via thermodynamics) by only the aid of computational tools beginning the study with crystallographic data obtained by X-ray diffraction. Given the fundamentally theoretical nature of this study, a comparison with experimental data from the literature is essential. Biran et al.^31^ and Elizebath et al.^32^ describe crystal growth initially guided by strongly stabilizing interactions, which then form supramolecular chains (1D). These chains then interact with each other to form two-dimensional structures (2D, supramolecular layers). Finally, the interaction between layers results in mesoscale (3D) structures. Our model was based on these recent experimental observations. Our model presents some conditions for the nucleation process to be completed: all interactions present in the first coordination sphere must occur during growth and growth must necessarily occur along the three direction axes (*a, b*, and *c*). By meeting these requirements and following the hierarchy of interactions, this process can be completed, and the result of applying this model is shown in Figures 6–8.

**Figure 6:**
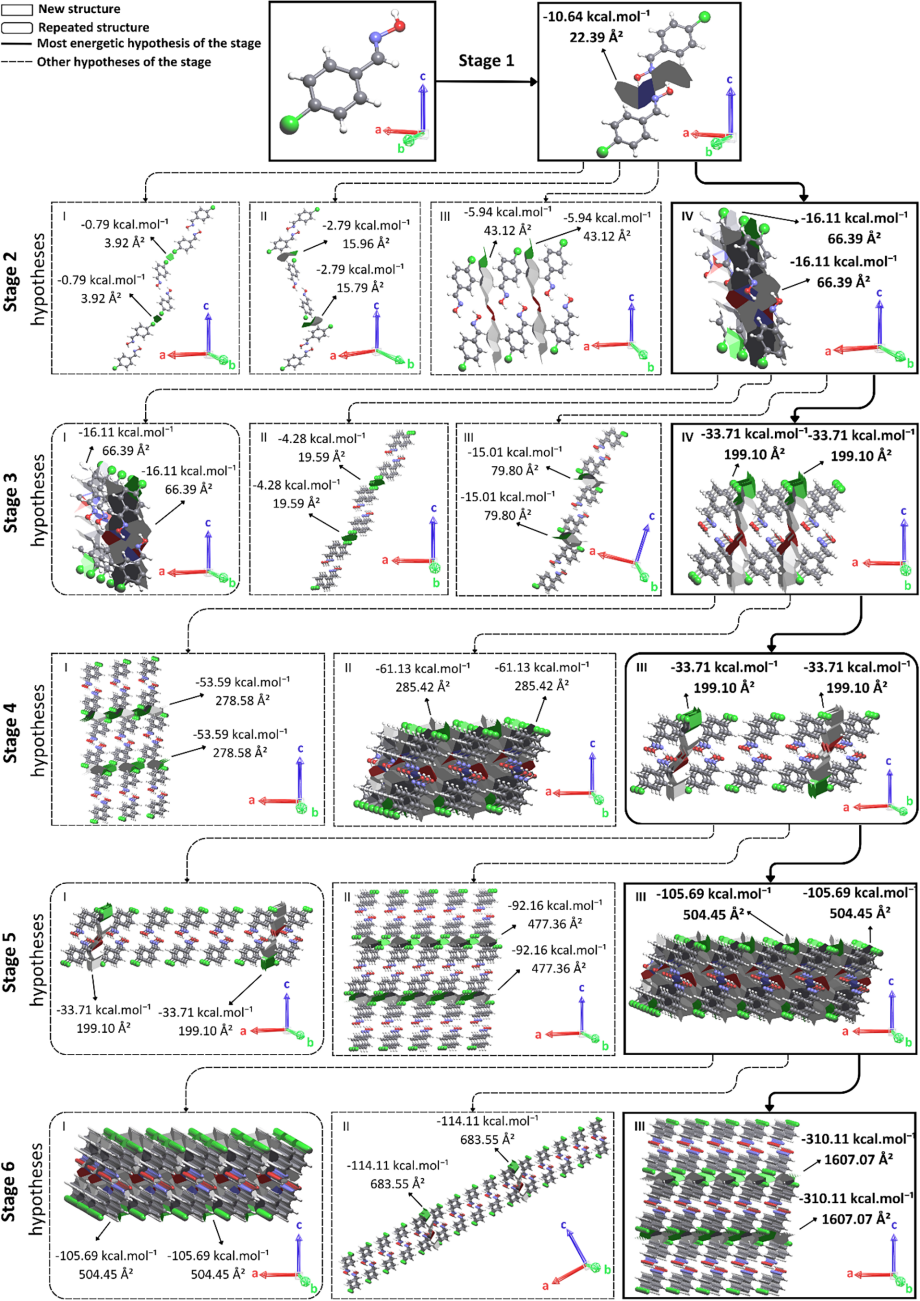
Proposed mechanism for compound *(E)-p*-Cl. For better understanding, access Video S1.

When initiating the crystallization mechanism, hypotheses are determined to create a sequential logic of supramolecular and subsequent crystalline growth. As a tool for comparing the proposed hypotheses, a comparison of the calculated stabilization energies is used. For example, if the stabilization energy of hypothesis II is greater than that of hypothesis I, then hypothesis II occurs first. Previous work by our research group^45,46^ has identified factors that influence the final number of steps in the proposed crystallization mechanisms. These factors include the expansion of a previous step, formation of a dimeric structure, and growth along an axis already observed in a previous step.

Stage 1 of the proposed crystallization mechanism of benzoxime *(E)-p*-Cl begins with the O–H···N interaction, which has an energy of −10.64 kcal·mol^–1^ and forms a contact area of 22.39 Å^2^, resulting in a supramolecular dimer (Figure 6).

In stage 2, four distinct hypotheses are suggested. The first hypothesis suggests growth in the direction of the *c* axis, guided only via a σ-hole that represents an area of 3.92 Å^2^ and contributes to an energy of −0.79 kcal·mol^–1^. Hypothesis II suggests growth in the direction of the *c* axis. This hypothesis presents an energy of −2.79 kcal·mol^–1^ and an area of 15.96 Å^2^. In hypothesis III, the dimer approximation results in a contact area of 43.12 Å^2^. The growth suggested in this hypothesis is along the *a* axis and the energy is −5.94 kcal·mol^–1^. In hypothesis IV, π-stacking should occur, forming a supramolecular chain with growth along the *b* axis. This hypothesis has a contact area of 66.39 Å^2^ and a stabilization energy of −16.11 kcal·mol^–1^. Therefore, the growth of stage 2 should be guided by hypotheses IV (Figure 6).

In stage 3, four hypotheses were suggested. Hypothesis I presents an expansion of the previous step; that is, growth occurs in the same direction, from the same interactions, and with the same contact area, only the energy is considered double, being −32.22 kcal·mol^–1^. In Hypothesis II, the approximation of supramolecular chains occurs along the *c* axis, resulting in a stabilization energy of −4.48 kcal·mol^–1^ and a contact area equal to 19.59 Å^2^. Hypothesis III suggests growth that results in a contact area of 79.80 Å^2^ and a stabilization energy of −15.01 kcal·mol^–1^. In Hypothesis IV, growth should occur along the *a*-axis, resulting in an energy of −33.71 kcal·mol^–1^ and a contact area of 199.10 Å^2^. Step 3 should be guided by the growth described in hypothesis IV, forming a supramolecular layer, because this approximation presents greater stabilization for the system.

In step 4, only three hypotheses are suggested. Hypothesis I suggests growth along the *c* axis, with stabilization energy and contact area equal to −53.59 kcal·mol^–1^ and 278.58 Å^2^, respectively. In Hypothesis II, the suggested growth results in a contact area of 285.42 Å^2^ and a stabilization energy of −61.13 kcal·mol^–1^. Hypothesis III describes an expansion of the previous step, resulting in an energy of −67.42 kcal·mol^–1^, with the same area and growth axis as hypothesis IV of step 3. Therefore, hypothesis III should guide the growth of step 4, increasing the supramolecular layer along the *a* axis.

Step 5 presents three distinct hypotheses for supramolecular approximations. Hypothesis I suggests an expansion of the previous step, maintaining the same information presented in Hypothesis III of Step 4, while Hypothesis II presents an increase in the direction of the *c*-axis, with contact area and stabilization energy values equal to 477.36 Å^2^ and −92.16 kcal·mol^–1^, respectively. Finally, Hypothesis III should present an increase along the *b*-axis with a contact area of 504.45 Å^2^ and a stabilization energy equal to −105.69 kcal·mol^–1^. Therefore, the growth of Step 5 should be driven by Hypothesis III, increasing the supramolecular layer along the *b*-axis.

In step 6, we suggest three approximation hypotheses. Hypothesis I suggests an expansion of the previous step, resulting in a stabilization energy of −211.28 kcal·mol^–1^. Hypothesis II suggests growth along the *a*-axis, resulting in a stabilization energy and contact area of −114.11 kcal·mol^–1^ and 683.55 Å^2^, respectively. Hypothesis III suggests growth along the *c*-axis, with a contact area of 1607.07 Å^2^ and a stabilization energy of −310.11 kcal·mol^–1^. Stage 6’s growth should be guided by hypothesis III, which presents the highest stabilizing energy value. This growth results in a three-dimensional structure that exhibits all of the interactions present in the first coordination sphere, as can be seen in Table 2. Therefore, the proposed crystallization mechanism of *(E)*-p-Cl concludes with six stages (Figure 6, Table 3, and video S1).

**Table 3:** Summary of the Proposed Crystallization Mechanism for *(E)-p*-Cl

**(** ***E*** **)-** ***p*** **-Cl**							
**hypotheses**	**description**	**stage 1**	**stage 2**	**stage 3**	**stage 4**	**stage 5**	**stage 6**
**I**	stabilization energy	**–10.64 kcal·mol** ^**–1**^	–0.79 kcal·mol^–1^	–16.11 kcal·mol^–1^	–53.59 kcal·mol^–1^	–67.41 kcal·mol^–1^	–105.69 kcal·mol^–1^
	contact area	**22.39 Å** ^**2**^	3.92 Å^2^	66.39 Å^2^	278.58 Å^2^	199.10 Å^2^	504.45 Å^2^
	intermolecular interaction	**O–H···N**	Cl···Cl	π stacking	C–H···Cl	C–H···O and C–H···Cl	π stacking
	growth axis		*c*	*b*	*c*	*a*	*b*
**II**	stabilization energy		–2.79 kcal·mol^–1^	–4.48 kcal·mol^–1^	–61.13 kcal·mol^–1^	–92.16 kcal·mol^–1^	–114.11 kcal·mol^–1^
	contact area		15.96 Å^2^	19.59 Å^2^	285.42 Å^2^	477.36 Å^2^	683.55 Å^2^
	intermolecular interaction		C–H···C and C–H···Cl	Cl···Cl	π stacking	Cl···Cl and C–H···Cl	C–H···O and C–H···Cl
	growth axis		*c*	*c*	*b*	*c*	*a*
**III**	stabilization energy		–5.94 kcal·mol^–1^	–15.01 kcal·mol^–1^	**–67.41 kcal·mol** ^**–1**^	**–105.69 kcal·mol** ^**–1**^	**–310.11 kcal·mol** ^**–1**^
	contact area		43.12 Å^2^	79.80 Å^2^	**199.10 Å** ^**2**^	**504.45 Å** ^**2**^	**1607.07 Å** ^**2**^
	intermolecular interaction		C–H···O and C–H···Cl	C–H···Cl	**C–H···O and C–H···Cl**	***π*** **stacking**	**Cl···Cl and C–H···Cl**
	growth axis		*a*	*c*	***a***	***b***	***c***
**IV**	stabilization energy		**–16.11 kcal·mol** ^**–1**^	–**33.71 kcal·mol** ^**–1**^			
	contact area		**66.39 Å** ^**2**^	**199.10 Å** ^**2**^			
	intermolecular interaction		**π stacking**	**C–H···O e C–H···Cl**			
	growth axis		***b***	***a***			

An interesting point to be addressed in this work is the issue of isostructurality between compounds *(E)-p*-Cl and *(E)-p*-Br, in which the difference in the size of the halogen radius did not affect the proposed mechanism. The Supporting Information contains both a detailed description of the process and an illustrative figure of the proposed crystallization mechanism for the oxime *(E)-p*-Br (Figure S17). However, when performing this same analysis with oximes substituted with these same halogens in the ortho position, it is clear that isostructurality is not present in the proposed crystallization mechanisms. The para position in the aromatic ring is favorable for the occurrence of σ-hole interactions. In this position, the distance between the halogen and oxime group minimizes steric effects, allowing this type of interaction to be observed in all *para*-substituted compounds analyzed, regardless of the size of the atomic radius of the halogen. However, substitution at the *ortho* position brings the halogen closer to the oxime functional group, resulting in a steric hindrance that compromises the formation of the σ-hole, especially in the case of chlorine. As a result, the oxime *(E)-o*-Cl does not interact in a way to form interactions via σ-hole and ends up presenting a greater occurrence of C–H···X and O···X interactions in comparison with the oxime *(E)-o*-Br. In the oxime *(E)-o*-Br, the halogen has a larger radius and, consequently, a larger σ-hole, which allows the formation of halogen bonds of the type X···X (observed in the last stage of the proposed mechanism). This difference in the interaction pattern contributes to the distinct crystallization mechanisms observed among ortho-substituted compounds, explaining why substitution at this position does not exhibit isostructurality. These halogen bonds have also been observed in benzoic acids^42^ and benzamides^44^ *para*-substituted.

In 2001, Tiekink et al. synthesized the isomers *(E)*-4-bromobenzaldehyde oxime^51^ and *(Z)*-4-bromobenzaldehyde oxime,^52^ in this work abbreviated as *(E)-p*-Br and *(Z)-p*-Br, respectively. When the conversion of *p*-bromobenzaldehyde to *p-*bromobenzonitrile is incomplete, the isomers *(E)-*4-bromobenzaldehyde oxime and *(Z)-*4-bromobenzaldehyde oxime were obtained and were partially separated by column chromatography (diethyl ether/hexane gradient, which is the same solvent as the slow evaporation to obtain the crystals). For the *E* isomer, it was observed that the final crystal had a plate shape, while the *Z* isomer had a needle shape (distinct crystal morphology). By applying our model to propose crystallization mechanisms, it was also possible to verify the difference in the crystallization pathway when comparing the *E* and *Z* isomers of oximes *p*-Cl and *p*-Br. The proposed crystallization mechanism for the oxime *(Z)-p*-Cl is described below; the corresponding mechanism for the oxime *(Z)-p*-Br is available in the Supporting Information (Figure S18).

Nucleation begins with the most energetic interaction in the first coordination sphere. In stage 1, π stacking should occur along the *b* axis, with each interaction having an energy of −5.73 kcal·mol^–1^ and a contact area of 27.79 Å^2^, for *(Z)-p*-Cl as can be seen in Figure 7. In stage 2, five different approximation hypotheses are investigated. Hypothesis I suggests an expansion of the previous stage; therefore, its energy is assumed to be double for the purpose of competition between hypotheses. Thus, the energy and area of hypothesis I are, respectively, −11.46 kcal mol^–1^ and 27.79 Å^2^. In hypothesis II, two supramolecular chain approximations identical to the initial chain are indicated with growth along the *ac* plane. These approximations result in an energy of −9.71 kcal·mol^–1^ and an area of 60.97 Å^2^. Hypothesis III points to an approximation of two supramolecular chains identical to the initial chain along the *a* axis, presenting values of −12.65 kcal·mol^–1^ and 63.04 Å^2^ of stabilization energy and contact area, respectively. In hypothesis IV, an approximation of only one supramolecular chain (along the *c* axis) was proposed, resulting in a dimeric approximation that presents an energy of −16.78 kcal·mol^–1^ and an area of 89.54 Å^2^. In hypothesis V, a dimeric approximation should occur, forming a contact area of 40.53 Å^2^ and an energy of −20.85 kcal·mol^–1^. Hypothesis V should guide the growth in stage 2, where a supramolecular dimeric structure was formed (Figure 7).

**Figure 7:**
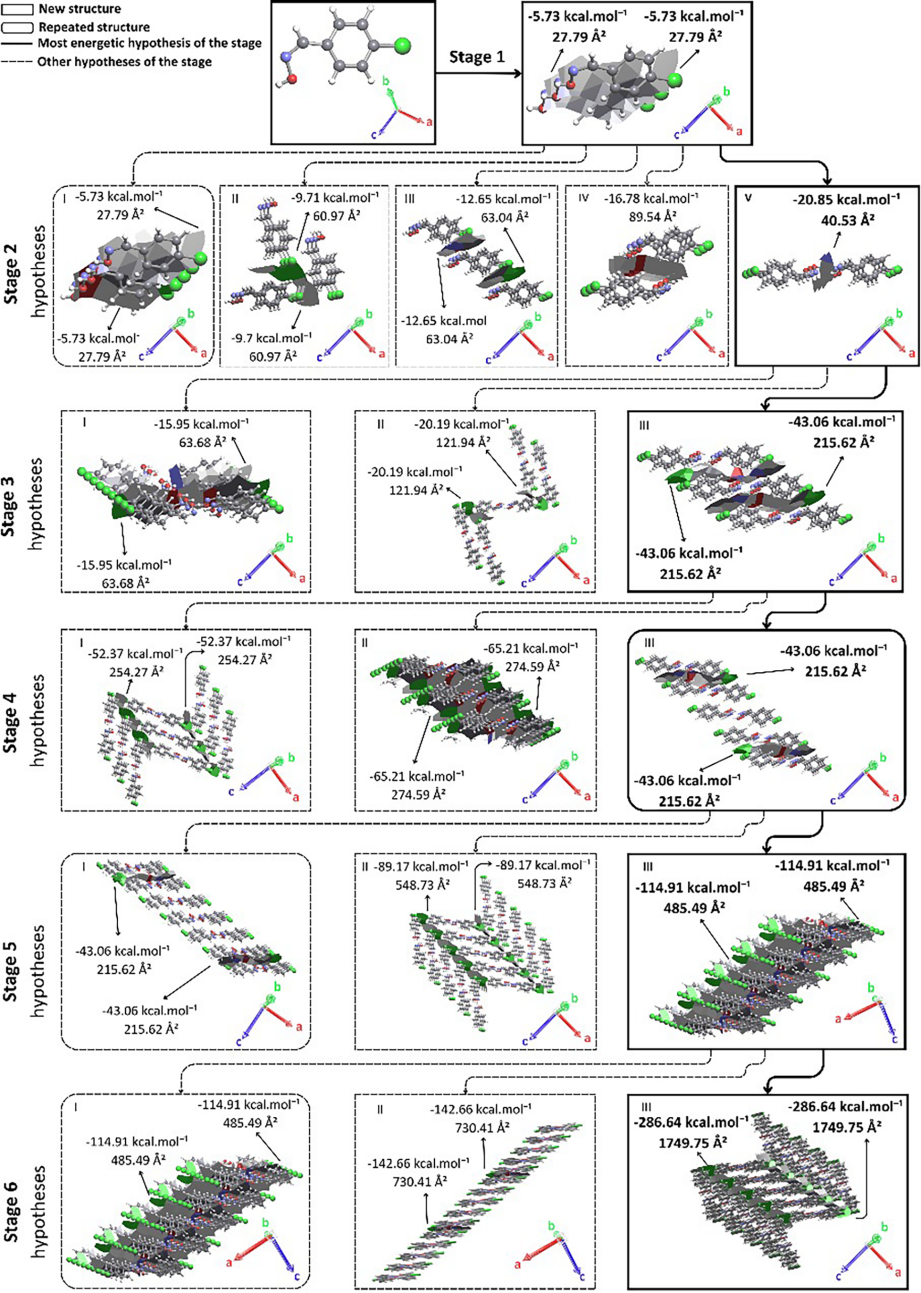
Proposed mechanism for compound *(Z)-p*-Cl. For better understanding, access Video S2.

In stage 3, only three hypotheses are suggested, with hypothesis I being π stacking (growth along the *b* axis), which results in an area of 63.68 Å^2^ and an energy of −15.95 kcal·mol^–1^. Hypothesis II suggests an approximation along the *ac* plane, which presents contact area and stabilization energy values equal to 121.94 Å^2^ and −20.19 kcal·mol^–1^, respectively. Hypothesis III indicates the occurrence of growth along the *a* axis, resulting in an area of 215.62 Å^2^ and a stabilization energy of −43.06 kcal·mol^–1^. The supramolecular growth in stage 3 of the proposed mechanism should be driven by hypothesis III, from the formation of a supramolecular layer (Figure 7).

In stage 4, three hypotheses are investigated. Hypothesis I suggests an approximation along the *ac* plane, through, which have an energy of −52.37 kcal·mol^–1^ and a contact area of 254.27 Å^2^. Hypothesis II represents π stacking along the *b* axis, increasing the depth of the previously formed layer. This hypothesis presents energy and contact area values equal to −65.21 kcal·mol^–1^ and 274.59 Å^2^, respectively. Hypothesis III indicates an expansion of the previous stage, in which growth occurs along the *a* axis, with the same area and the same interactions as hypothesis III of stage 3, although the value of the stabilization energy considered is double (−86.12 kcal·mol^–1^). Supramolecular growth in stage 4 should occur according to hypothesis III, increasing the layer formed in a direction already observed in the previous stage (Figure 7).

In stage 5, three hypotheses are investigated. Hypothesis I suggests another expansion, following the same interactions and the same value for the area. For comparison purposes with the other hypotheses, the energy considered is −86.12 kcal·mol^–1^. Hypothesis II presents growth along the *ac* plane, which results in energy and contact area values of −89.17 kcal·mol^–1^ and 548.73 Å^2^, respectively. In hypothesis III, a new π stacking should occur along the *b* axis, which results in a stabilizing energy of −114.91 kcal·mol^–1^ and presents a contact area of 485.49 Å^2^. Because it is the most energetically favorable hypothesis, hypothesis III should guide the growth of stage 5, increasing the depth of the already formed layer and repeating a growth in a direction already observed (Figure 7).

In stage 6, three distinct hypotheses are suggested, with hypothesis I being π stacking (growth along the *b* axis), which presents contact area and stabilization energy values equal to 485.49 Å^2^ and −114.91 kcal·mol^–1^, respectively. Hypothesis II points to growth along the *a* axis, which results in an energy of −142.66 kcal·mol^–1^ and a contact area of 730.41 Å^2^. In hypothesis III, growth should occur along the *ac* plane, which forms a contact area of 1749.75 Å^2^ and results in an energy of −286.64 kcal·mol^–1^. Hypothesis III should guide the growth in stage 6, finalizing the proposed crystallization mechanism, forming a three-dimensional structure that includes all the interactions present in the first coordination sphere of the *(Z)-p*-Cl oxime (Figure 7, Table 4, and video S2).

**Table 4:** Summary of the Proposed Crystallization Mechanism for *(Z)-p*-Cl

**(** ***Z*** **)-** ***p*** **-** ***Cl***							
**hypothesis**	**description**	**stage 1**	**stage 2**	**stage 3**	**stage 4**	**stage 5**	**stage 6**
**I**	stabilization energy	**–5.73 kcal·mol** ^**–1**^	–11.46 kcal·mol^–1^	–15.95 kcal·mol^–1^	–52.37 kcal·mol^–1^	–86.12 kcal·mol^–1^	–229.82 kcal·mol^–1^
	contact area	**27.79 Å** ^**2**^	27.79 Å^2^	63.68 Å^2^	254.27 Å^2^	215.62 Å^2^	485.49 Å^2^
	intermolecular interaction	**π-stacking**	π-stacking	π-stacking	C–H···H–C and C–H···Cl	C–H···H–C and C–H···O	π-stacking
	growth axis	***b***	*b*	*b*	*ac plane*	*a*	*b*
**II**	stabilization energy		–9.71 kcal·mol^–1^	–20.19 kcal·mol^–1^	–65.21 kcal·mol^–1^	–89.17 kcal·mol^–1^	–142.6 kcal·mol^–1^
	contact area		60.97 Å^2^	121.94 Å^2^	274.59 Å^2^	548.73 Å^2^	730.41 Å^2^
	intermolecular interaction		C–H···H–C and C–H···Cl	C–H···H–C and C–H···Cl	π-stacking	C–H···H–C and C–H···Cl	C–H···H–C, C–H···Cl and C–H···O
	growth axis		*ac plane*	*ac plane*	*b*	*ac plane*	*ac plane*
**III**	stabilization energy		–12.65 kcal·mol^–1^	**–43.06 kcal·mol** ^**–1**^	**–86.12 kcal·mol** ^**–1**^	**–114.91 kcal·mol** ^**–1**^	**–286.64 kcal·mol** ^**–1**^
	contact area		63.04 Å^2^	**215.62 Å** ^**2**^	**215.62 Å** ^**2**^	**485.49 Å** ^**2**^	**1749.75 Å** ^**2**^
	intermolecular interaction		C–H···H–C and C–H···Cl	**C–H···H–C, C–H···Cl and C–H···O**	**C–H···H–C, C–H···Cl and C–H···O**	**π-stacking**	**C–H···H–C and C–H···Cl**
	growth axis		***a***	***a***	***a***	***b***	***ac plane***
**IV**	stabilization energy		–16.78 kcal·mol^–1^				
	contact area		89.84 Å^2^				
	intermolecular interaction		C···O, C–H···N and C–H···O				
	growth axis		*c*				
**V**	stabilization energy		**–20.85 kcal·mol** ^**–1**^				
	contact area		**40.53 Å** ^**2**^				
	intermolecular interaction		**O–H···N**				
	growth axis						

In addition to the geometric isomers, the present study also addresses the differences in the proposed crystallization mechanisms for the planar isomers of the compound C_7_H_6_ClNO, namely: (*E)-o*-Cl and (*E)-m*-Cl, represented in Figure 1. Detailed descriptions of the proposed mechanisms are found in the Supporting Information, accompanied by figures that illustrate the hypotheses considered in the construction of this process (Figure S14, Video S3 and Figure S16, Video S4). Figure 8 presents the proposed crystallization mechanisms for both isomers, highlighting only the energetically favored hypothesis at each stage. Interestingly, it is worth noting that the *(E)-o*-Cl isomer must initiate its crystallization mechanism from N–H···O hydrogen bonds, followed by π stacking. The *(E)-m*-Cl isomer must initiate from π stacking, followed by the formation of N–H···O hydrogen bonds. This same characteristic was observed in the substituted p-Cl and p-Br compounds (Figure S10). The geometric isomers (E) of each pair of compounds showed the initiation of nucleation from the dimeric N–H···O hydrogen bond, followed by pi stacking, whereas the isomers (*Z*) exhibited the reverse process. In addition to the presented isomeric compounds, the present study includes the investigation of other nonisomeric compounds, as represented in Figure 1. The discussion on the proposed crystallization mechanisms for (*E*)-*o*-Br (Figure S15, Video S5), (*E*)-*p*-F (Figure S17, Video S6), and (*E*)-*p*-I (Figure S20, Video S7) are presented in the Supporting Information.

**Figure 8:**
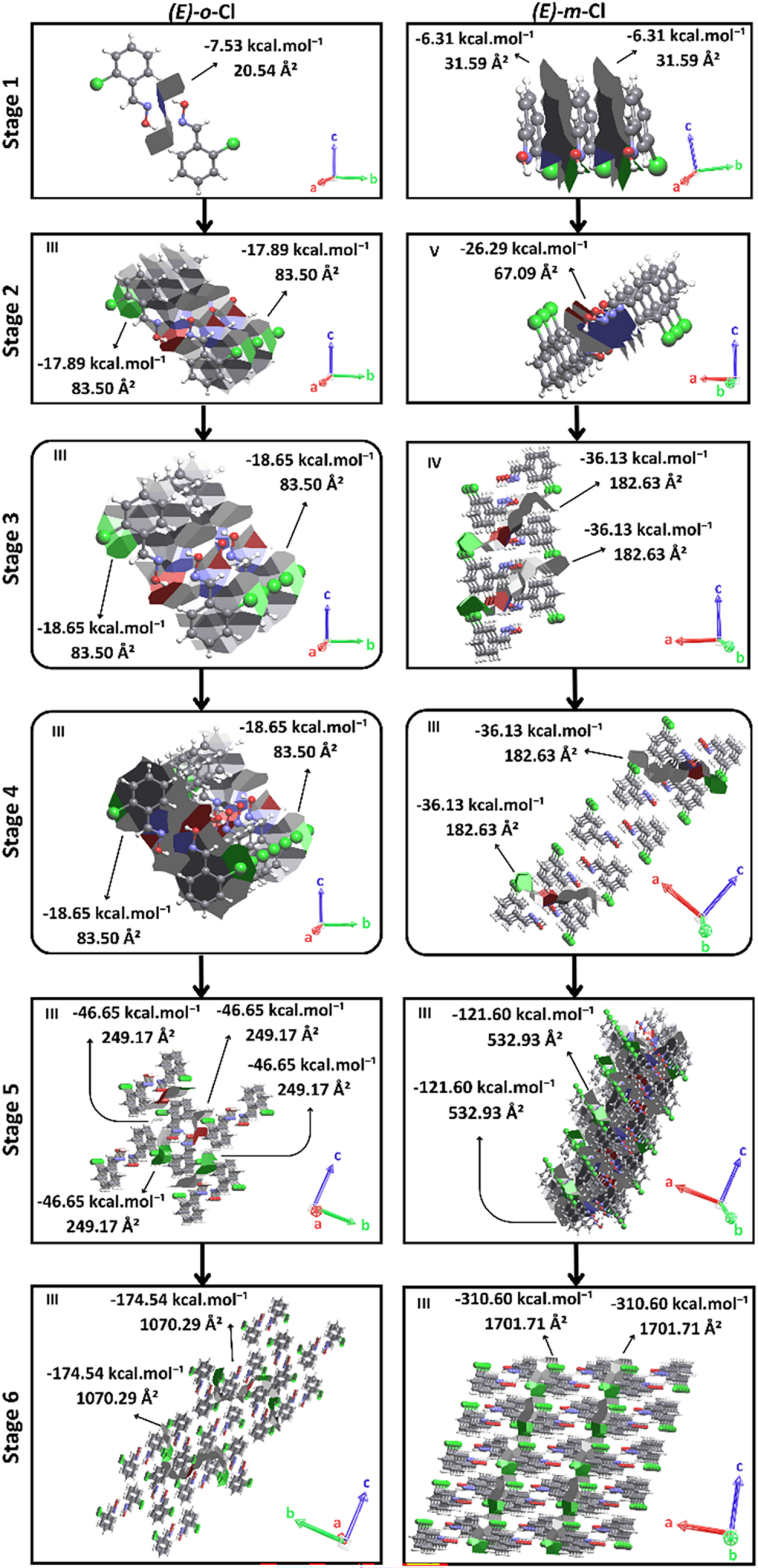
Simplified crystallization mechanism proposals for compounds *(E)-o*-Cl and *(E)-m*-Cl.

One of the topics addressed by our model concerns the order of orientation of the proposed supramolecular growth along the axes of the unit cell. In most cases, we observed a tendency for initial growth to occur along the shortest axis, followed by the intermediate-length axis and, finally, the longest axis, according to the hierarchy of stabilization energies of the interactions involved. Among the nine compounds studied, only two compounds (*(E)-m*-Cl and *(E)-p*-I) did not follow this trend. Both showed initial growth along the minor axis, but as the mechanism progressed, the growth deviated from the expected direction. However, upon comparison of the lengths of the axes along which this inversion occurred, it is noted that they are approximately the same size, which may explain this phenomenon. The remaining halogenated benzoximes showed the standard growth trend according to the model (from the smallest axis to the largest axis). This reinforces that the order of crystal growth along the cell axes is directly associated with the crystal morphology.

## 3 Conclusion

This study was based on a model developed by our research group, which describes crystallization mechanisms at the molecular level based on theoretical data on the energy and topology of intermolecular and supramolecular interactions. The interactions were verified by using QTAIM, and their robustness was assessed energetically. The model was applied to *ortho*-, *meta*-, and *para*-substituted halogenated aryl oximes. Nine oximes were analyzed, and only *(E)-p*-I presented a molecular coordination number (MCN) different from 14, with a value equal to 13. A correlation was observed between the theoretical cohesive energy of the first coordination sphere data and the experimental melting point data of the compounds, which proved to be reliable since there was a correlation between these data. The iodine atom has a large volume and high polarizability, which intensifies the strength of its intermolecular interactions. However, the larger atomic radius also reduces packing efficiency due to strong steric hindrances, as evidenced by its MCN = 13 (lower than the others at 14). This can result in lower cohesion of the final crystal structure, also affecting the melting point. Furthermore, the unit cell of compound (*E*)-*p*-I has *Z* = 2, and the unit cells of the other *para*-substituted compounds have *Z* = 4, which influences the lower packing of compounds containing iodine, lowering the melting point.

Regarding the proposed crystallization mechanisms, it was found that the formation of the O–H···N dimer was not the strongest interaction present in the first coordination sphere in all the compounds studied. The exceptions were the oximes *(E)-m*-Cl, *(Z)-p*-Cl, and *(Z)-p*-Br, which presented π stacking as the first stage in nucleation. In these cases, the sum of weaker interactions of the C···C and C–H···C types highlighted the importance of considering all interactions energetically.

Among the factors contributing to the increase in the number of steps in the mechanism, the following expansion cases stand out: stages 3 and 4 of *(E)-o*-Cl, stage 3 of *(E)-o*-Br, and stage 4 of *(E)-m*-Cl, *(E)-p*-F, *(Z)-p*-Cl, and *(Z)-p*-Br. Furthermore, growth along a previously observed direction occurred in stage 5 of *(E)-p*-Cl, *(E)-p*-Br, *(Z)-p*-Cl, *(Z)-p*-Br, *(E)-m*-Cl, and *(E)-p*-F. Finally, dimer formation stands out as the third factor, observed in stage 1 of the compounds *(E)-o*-Cl, *(E)-o*-Br, *(E)-p*-F, *(E)-p*-Cl, *(E)-p*-Br, and *(E)-p*-I, and in step 2 of *(E)-m*-Cl, *(Z)-p*-Cl, and *(Z)-p*-Br. All proposed mechanisms were concluded after the occurrence of all interactions present in the cluster with the formation of a three-dimensional structure.

The variation in the position of the halogen caused major changes in the proposed crystallization mechanisms and, consequently, in the crystal packing, while a change in the halogen in the benzene ring did not cause major changes for *para*-benzoximes, unlike for orthobenzoximes, where it was observed that the change from chlorine to bromine significantly affects the proposed mechanisms due to steric hindrance that compromises the formation of the σ-hole, especially in the case of chlorine. The benzoxime pairs *(E)-p*-Cl and *(E)-p*-Br, as well as *(Z)-p*-Cl and *(Z)-p*-Br are isostructural, which presents proposals of analogous crystallization mechanisms. Furthermore, the application of our model was able to propose crystallization mechanisms with distinct trajectories for the isomeric oximes *(E)-p*-Br and *(Z)-p*-Br, highlighting the difference in the morphologies observed experimentally, in which *(E)-p*-Br has a plate-like crystalline habit and *(Z)-p*-Br has a needlelike crystalline habit, according to the final supramolecular structure obtained. Understanding the crystallization process at the molecular level is essential for developing crystalline structures with desirable physical or biological properties. Therefore, the authors believe that these results are the first step toward understanding the crystallization process at the molecular level and extending the proposed analysis to other functional groups. As long as the computational cost required for reliable calculations for more complex functional groups is taken into account, this approach presented by the authors can be extended to other compounds, whether halogenated or not.

## 4 Experimental Section

The single-crystal X-ray diffraction crystallographic data was obtained from the Cambridge Structural Database (CSD), using ConQuest Version 2022.3.0.^65^ The compounds studied have the following CCDC/CSD refcodes: TECJOB for *o*-Cl, AYULIQ for *o*-Br, JIYYEY for *m*-Cl, JIYYIC for *p*-F, CBALOS02 for *p*-Cl, BAGWOW for *p*-Br, and GIKSOK for *p*-I. Initially, the molecular coordination number (MCN)^66^ of each compound and the contact surface (M_1_···M_n_) between the molecules in the cluster were determined by the Voronoi–Dirichlet polyhedron (VDP),^56^ using the ToposPro program.^67^ The Hirshfeld surfaces were determined using the Crystal Explorer program.^68,69^ In this work, the stabilization energy of the intermolecular interactions of each pair of molecules selected in the first coordination sphere was calculated. This value was obtained from the difference between the energy of a Mn molecule interacting with the M_1_ molecule (*E*_M1···Mn_) and the energy of twice an isolated M1 molecule (*E*_M1_) using the equation *G*_M1···Mn_ = *E*_M1···Mn_–2*E*_M1_.^35^ This energy is used to describe the first stage of the crystallization mechanism. For the other stages, the same method is used; however, the SMc···SMn interaction occurs between two equal supramolecular structures, as expressed in the equation *G*_SMc···SMn_ = *E*_SMc···SMn_–2*E*_SMc_. When we have the possibility of expanding the previous stage, the stabilizing energy is determined by the SMc···2SMn interaction between a central supramolecular structure and two supramolecular structures from the previous stage, as shown in the equation *G*_SMc···2SMn_ = *E*_SMc···2SMn_–*E*_SMc_–2*E*_SMn_. The cohesive energy of the first coordination sphere is determined by the sum of the stabilizing energy of the intermolecular interactions of the M1···Mn dimers of the first coordination sphere; in the presence of identical dimers (same interatomic distance, contact area, interaction energy, and electronic density), the energy of only one of these dimers is considered for the calculation since it is given per mol. This energy was then correlated with the melting point.^70^ These energies were determined by single point gas phase calculations (no structural optimization) at the ωB97X-D3 ^60^ level of theory, using the def2-TZVP^61^ basis set and the RIJCOSX^71^ approximation with the auxiliary bases def2/J^72^ and def2-TZVP/C^73^ in the ORCA (Version 5.0.3) program.^74^ All compounds used the default PModel initial guess method from the version of ORCA 5.0.3. The basis set superposition error (BSSE) was calculated using Boys and Bernardi’s counterpoise (CP) method. The interactions involved, as well as their electronic densities, were determined by ORCA 5.0.3,^74^ MultiWFN^75^ and AIMAII (Version 10.05.04).^76^ The inputs for the energy calculations were made in the Avogadro (version 1.2.0) program. The figures were made using ToposPro,^67^ Crystal Explorer,^68,69^ and Mercury (Version 2022.3.0).^77^

## Supplementary Material

















## Notes

In the Supporting Information you will find tables and figures showing the intermolecular interactions with their data (PDF)
Animation of the proposed crystallization mechanism of the *(E)-p*-Cl compound (video S1 mp4) (MP4)
Animation of the proposed crystallization mechanism of the (Z)-*p*-Cl compound (video S2 mp4) (MP4)
Animation of the proposed crystallization mechanism of the *(E)-o*-Cl compound (video S3 mp4) (MP4)
Animation of the proposed crystallization mechanism of the *(E)-m*-Cl compound (video S4 mp4) (MP4)
Animation of the proposed crystallization mechanism of the *(E)*-*o*-Br compound (video S5 mp4)­(MP4)
Animation of the proposed crystallization mechanism of the *(E)-p*-F compound (video S6 mp4) (MP4)
Animation of the proposed crystallization mechanism of the *(E)-p*-I compound (video S7 mp4) (MP4)

## Abbreviations

**1D**, **2D** and **3D**: 1, 2, and 3 dimensions, respectively
BCP: bond critical point
BSSE: basis set superposition error
CCDC: Cambridge crystallographic data centre
CP: counterpoise
CSD: Cambridge structural database
**D**_**%**_: percentage discrepancy
D3: dispersion correction
**D**_**abs**_: absolute discrepancy
DFT: density functional theory
**E**_**M1**_: total energy of the central molecule
**E**_**M1···Mn**_: total energy of between the central molecule and the *n* molecule
**E**_**Mn**_: total energy of the *n* molecule
**E**_**SMc**_: total energy of the central supramolecular structure
**E**_**SMc···SMn**_: total energy between the central supramolecular structure and the *n* supramolecular structure
**E**_**SMc···2SMn**_: total energy between the central supramolecular structure and the 2 *n* supramolecular structure
**E**_**SMn**_: total energy of the *n* supramolecular structure
**G**_**M1···Mn**_: energy of interaction between the central molecule and the *n* molecule
**G**_**SMc···SMn**_: energy of interaction between the central supramolecular structure and the *n* supramolecular structure
**G**_**SMc···2SMn**_: energy of interaction between the central supramolecular structure and 2 *n* supramolecular structures
M1: central molecule
MCN: molecular coordination number
Mn: *n* molecule
QTAIM: quantum theory of atoms in molecules
SE: sublimation enthalpy
VDP: voronoi-dirichlet polyhedron
ΔH: sublimation enthalpy change
ρ: electron density
