# Supplementary material for: Influence of Halogen and Its Position on Crystal Packing: Proposals for Molecular-Level Crystallization Mechanisms of Isomeric Halogenated Benzoximes
Source: ACS Omega. 2025 Dec 16;10(51):63028–43. doi: 10.1021/acsomega.5c08976 (PMC12756728; doi:10.1021/acsomega.5c08976)
Supplement: Supplementary file 1 [file ao5c08976_si_001.pdf]

Supporting Information to

# Influence of halogen and its position on crystal packing: Proposals for molecular-level crystallization mechanisms of isomeric halogenated benzoximes


Patrick Teixeira Campos[1*], Isabella Burchardt Ferreira[1], Pedro Henrique Cunha do Couto[1], Davi Fernando Back[2]

[1] Laboratório de Química Orgânica Sintética, Estrutural e Computacional (LaQuiOSEC), Instituto Federal de Educação, Ciência e Tecnologia Sul-rio-grandense (IFSul) – Câmpus Pelotas, CEP 96015-360, Pelotas, Brazil

[2] Laboratório de Materiais Inorgânicos (LMI), Departamento de Química – UFSM, Av. Roraima 1000, 97105-900 Santa Maria, RS, Brazil


# Table of Contents


**Figure S1.** First coordination spheres of all compound studied
**Figure S2.** Hirshfeld surfaces of the compound (E)-p-F: (a) Surface on the central molecule, M1, surrounded by six molecules of the principal plane, (b) Surface on the six molecules surrounding M1, (c) Surface of all molecules of the principal plane and (d) Surface of M1 with the upper planes.
**Table S1.** Supramolecular cluster data of (E)-*o*-Br
**Table S2.** Supramolecular cluster data of (E)-*m*-Cl
**Table S3.** Supramolecular cluster data of (E)-*p*-F
**Table S4.** Supramolecular cluster data of (E)-*p*-Cl
**Table S5.** Supramolecular cluster data of (Z)-*p*-Cl
**Table S6.** Supramolecular cluster data of (E)-*p*-Br
**Table S7.** Supramolecular cluster data of (Z)-*p*-Br
**Table S8.** Supramolecular cluster data of (E)-*p*-I
**Figure S3.** Planes of the first coordination sphere for (E)-*o*-Cl
**Figure S4.** Planes of the first coordination sphere for (E)-*o*-Br
**Figure S5.** Planes of the first coordination sphere for (E)-*m*-Cl
**Figure S6.** Planes of the first coordination sphere for (E)-*p*-F
**Figure S7.** Planes of the first coordination sphere for (Z)-*p*-Cl
**Figure S8.** Planes of the first coordination sphere for (E)-*p*-Br
**Figure S9.** Planes of the first coordination sphere for (Z)-*p*-Br
**Figure S10.** Planes of the first coordination sphere for (E)-*p*-I
**Table S9.** Pairwise first-shell cohesive energy and melting point of each compound
**Figure S11.** Comparison between the contact areas and stabilization energies of the configurations present in the first coordination sphere of the oximes (E)-m-Cl and (E)-p-Cl. (a) π-stacking of the compound (E)-m-Cl. (b) π-stacking of the compound (E)-p-Cl. (c) Pair of molecules M1···M2 of the oxime (E)-m-Cl (with O-H···N and C-H···O interactions). (d) Supramolecular dimer M1···M2 of the oxime (E)-p-Cl (with O-H···N interactions).
**Figure S12.** Number of occurrences and energetic contribution of each class of intermolecular interaction for all compounds.
**Figure S13:** Linear correlations between pairwise first-shell cohesive energy and melting points of compounds.
**Figure S14.** Crystallization mechanism proposal for (E)-*o*-Cl
**Figure S15.** Crystallization mechanism proposal for (E)-*o*-Br
**Figure S16.** Crystallization mechanism proposal for (E)-*m*-Cl
**Figure S17.** Crystallization mechanism proposal for (E)-*p*-F
**Figure S18.** Crystallization mechanism proposal for (E)-*p*-Br

**Figure S19.** Crystallization mechanism proposal for (Z)-*p*-Br
**Figure S20.** Crystallization mechanism proposal for (E)-*p*-I

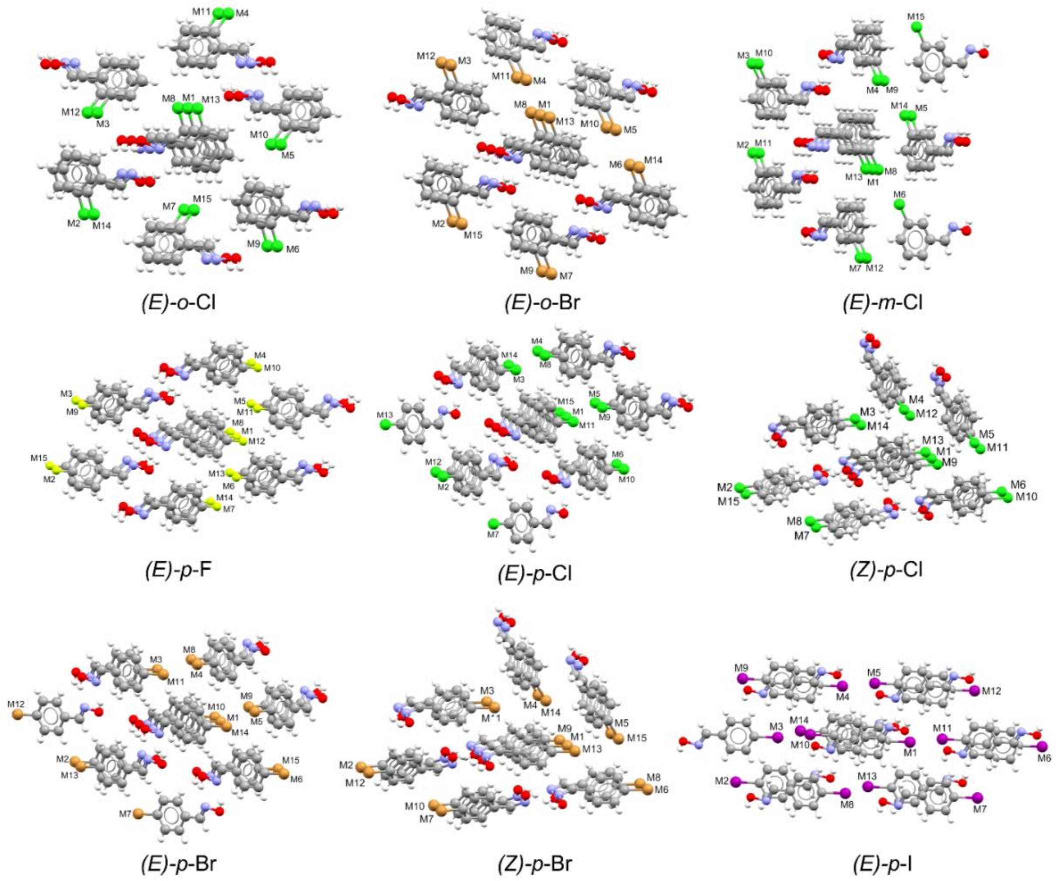


**Figure S1.** First coordination spheres of all compound studied.

A topological analysis based on the Hirshfeld surface (**Figure S2**) was performed, which highlights areas of contact between the central molecule and some peripheral molecule, expanding the analysis beyond the approach of interatomic distances equal to or less than the sum of the van der Waals radii, which avoids the exclusion of any molecule from the study. The Hirshfeld surface allows the identification of a greater number of intermolecular interactions, and the interatomic distance, in this context, serves as a criterion for comparing the intensities of similar contacts. The Crystal Explorer software [56,57] was used to represent the Hirshfeld surfaces,[58] made from the defined planes of the *(E)*-*p*-Cl oxime. The red regions are the regions of greatest electron density and, therefore, are the regions around the molecules in which the most intense intermolecular interactions occur.

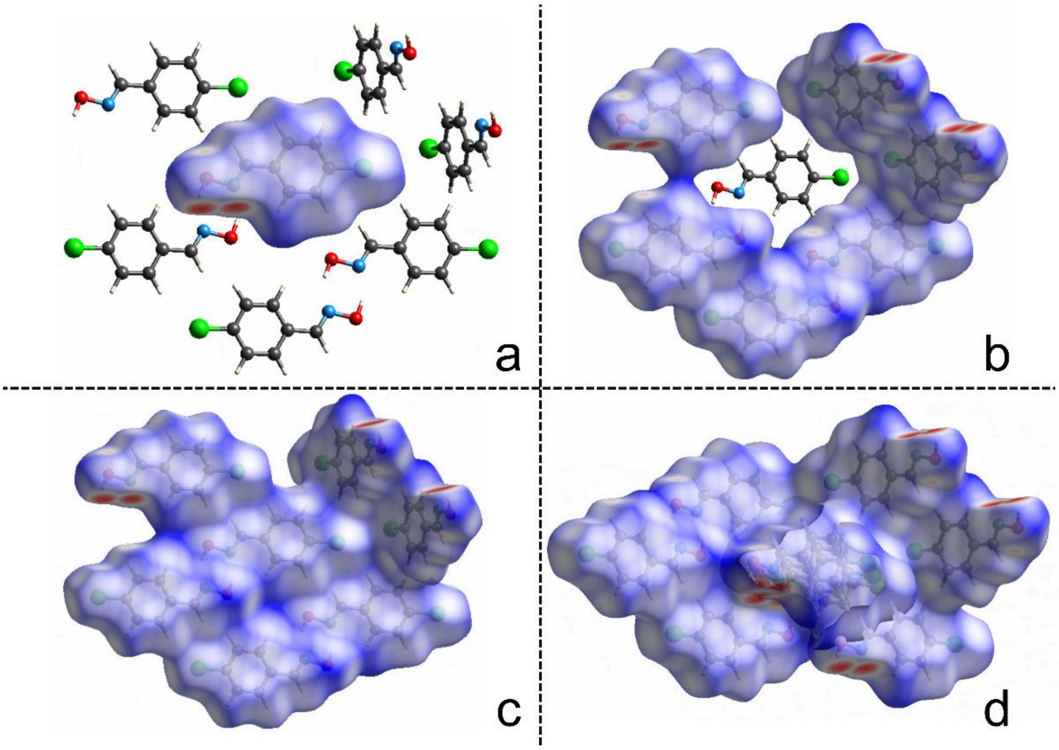


**Figure S2**: Hirshfeld surfaces of the compound (E)-p-Cl: (a) Surface on the central molecule, M1, surrounded by six molecules of the principal plane, (b) Surface on the six molecules surrounding M1, (c) Surface of all molecules of the principal plane and (d) Surface of M1 with the upper planes.

**Table S1:** Pair of molecules M1···Mn, contact area, interaction energy, interaction, interatomic distance, contact energy, contribution percentage and electron density for the supramolecular cluster of *(E)-o*-Cl.

| Dimer | Contact Area[a] (Å$^2$) | Interaction Energy[b] (kcal.mol-1) | Interaction[c] | Interatomic distance (Å) | Contact energy[d] (kcal.mol-1) | Contribution percentage (%) | $\rho_{INT}$[c] (u.a.) |
|---|---|---|---|---|---|---|---|
| M1···M2 | 20.54 | -7,53 | N2···H1-O1 | 2.137 | -3,77 | 50,00 | 0,019191 |
| | | | O1-H1···N2 | 2.137 | -3,77 | 50,00 | 0,01919 |
| M1···M3 | 11.26 | -1,64 | Cl5···H7-C7 | 3.114 | -1,05 | 64,34 | 0,003599 |
| | | | C3-H3···H6-C6 | 3.010 | -0,43 | 26,10 | 0,00146 |
| | | | O1···Cl5 | 4.577 | -0,16 | 9,56 | 0,000535 |
| M1···M4 | 4.54 | -1,14 | Cl5···C8 | 3.900 | -1,14 | 100.00 | 0,002179 |
| M1···M5 | 12.72 | -2,21 | C6-H6···O1 | 2.552 | -2,16 | 97,77 | 0,007237 |
| | | | C7-H7···Cl5 | 4.709 | -0,05 | 2,23 | 0,000165 |
| M1···M6 | 13.15 | -0,91 | C7-H7···H8-C8 | 2.697 | -0,45 | 49,98 | 0,002436 |
| | | | C8-H8···H7-C7 | 2.697 | -0,45 | 50,02 | 0,002438 |
| M1···M7 | 9.33 | -1,47 | C8-H8···Cl5 | 3.143 | -0,66 | 44,94 | 0,003753 |
| | | | C9-H9···Cl5 | 3.054 | -0,81 | 55,06 | 0,004599 |
| M1···M8 | 32.33 | -7,40 | C8···C7 | 3.566 | -1,58 | 21,37 | 0,004538 |
| | | | C4···C5 | 3.533 | -1,55 | 20,95 | 0,004448 |
| | | | Cl5···Cl5 | 3.922 | -1,21 | 16,41 | 0,003485 |
| | | | C3···Cl5 | 3.704 | -1,28 | 17,29 | 0,00367 |
| | | | N2···C3 | 3.302 | -1,77 | 23,97 | 0,00509 |
| M1···M9 | 11.55 | -1,19 | C8-H8···H7-C7 | 2.821 | -0,60 | 49,99 | 0,002112 |
| | | | C7-H7···H8-C8 | 2.821 | -0,60 | 50,01 | 0,002113 |
| M1···M10 | 11.26 | -1,64 | Cl5···O1 | 4.576 | -0,16 | 9,56 | 0,000535 |
| | | | C6-H6···H3-C3 | 3.010 | -0,43 | 26,09 | 0,00146 |
| | | | C7-H7···Cl5 | 3.114 | -1,05 | 64,35 | 0,003601 |
| M1···M11 | 9.33 | -1,47 | Cl5···C8-H8 | 3.142 | -0,66 | 44,93 | 0,003752 |
| | | | Cl5···C9-H9 | 3.054 | -0,81 | 55,07 | 0,004598 |
| M1···M12 | 12.72 | -2,21 | O1···H6-C6 | 2.552 | -2,16 | 97,77 | 0,007239 |
| | | | Cl5···H7-C7 | 4.709 | -0,05 | 2,23 | 0,000165 |
| M1···M13 | 32.33 | -7,40 | C7···C8 | 3.566 | -1,58 | 21,37 | 0,004535 |
| | | | C5···C4 | 3.534 | -1,55 | 20,98 | 0,004452 |
| | | | Cl5···Cl5 | 3.924 | -1,21 | 16,42 | 0,003485 |
| | | | Cl5···C3 | 3.704 | -1,28 | 17,29 | 0,003669 |
| | | | C3···N2 | 3.302 | -1,77 | 23,95 | 0,005082 |
| M1···M14 | 18.84 | -2,86 | O1···H9-C9 | 3.036 | -0,83 | 29,16 | 0,003542 |
| | | | N2···N2 | 3.233 | -1,19 | 41,69 | 0,005064 |
| | | | C9-H9···O1 | 3.036 | -0,83 | 29,16 | 0,003542 |
| M1···M15 | 4.54 | -1,14 | C8···Cl5 | 3.900 | -1,14 | 100,00 | 0,002181 |

**a:** ToposPro; **b:** (GM1···Mn = EM1···Mn – 2*EM1) corrected by BSSE; **c:** QTAIM (MultiWFN); **d:** Ec = GCM1···Mn * Contribution percentage.

**Table S2:** Pair of molecules M1···Mn, contact area, interaction energy, interaction, interatomic distance, contact energy, contribution percentage and electron density for the supramolecular cluster of *(E)-o*-Br.

| Dimer | Contact Area[a] (Å$^2$) | Interaction Energy[b] (kcal.mol-1) | Interaction[c] | Interatomic distance (Å) | Contact energy[d] (kcal.mol-1) | Contribution percentage (%) | $\rho_{INT}$[c] (u.a.) |
|---|---|---|---|---|---|---|---|
| M1···M2 | 19.53 | -8.46 | N2···H1-O1 | 1.981 | -4.23 | 50.00 | 0.026556 |
| | | | O1-H1···N2 | 1.981 | -4.23 | 50.00 | 0.026556 |
| M1···M3 | 15.73 | -2.16 | Br5···H7-C7 | 3.361 | -0.66 | 30.59 | 0.003149 |
| | | | O1···H8-C8 | 2.572 | -1.50 | 69.41 | 0.007144 |
| M1···M4 | 5.07 | -0.88 | Br5···Br5 | 3.655 | -0.88 | 100.00 | 0.006662 |
| M1···M5 | 18.06 | -3.19 | Br5···C7 | 3.983 | -0.88 | 27.71 | 0.003283 |
| | | | C6-H6···C6 | 2.892 | -1.20 | 37.69 | 0.004465 |
| | | | C6-H6···Br5 | 3.236 | -1.10 | 34.60 | 0.004099 |
| M1···M6 | 15.73 | -2.16 | C7-H7···Br5 | 3.361 | -0.66 | 30.60 | 0.003149 |
| | | | C8-H8···O1 | 2.572 | -1.50 | 69.40 | 0.007143 |
| M1···M7 | 11.32 | -1.54 | C8-H8···H9-C9 | 2.561 | -0.77 | 50.00 | 0.002929 |
| | | | C9-H9···H8-C8 | 2.561 | -0.77 | 50.00 | 0.002929 |
| M1···M8 | 32.59 | -7.24 | N2···C4 | 3.364 | -1.42 | 19.65 | 0.00527 |
| | | | C3···Br5 | 3.583 | -1.53 | 21.09 | 0.005655 |
| | | | Br5···Br5 | 4.002 | -1.31 | 18.12 | 0.004858 |
| | | | C4···C6 | 3.518 | -1.47 | 20.26 | 0.005434 |
| | | | C9···C7 | 3.521 | -1.51 | 20.88 | 0.005599 |
| M1···M9 | 1.00 | -0.39 | N2···H8-C8 | 5.043 | -0.12 | 31.03 | 0.000054 |
| | | | C9-H9···H9-C9 | 4.302 | -0.15 | 37.93 | 0.000066 |
| | | | C8-H8···N2 | 5.043 | -0.12 | 31.03 | 0.000054 |
| M1···M10 | 18.06 | -3.19 | Br5···H6-C6 | 3.237 | -1.10 | 34.59 | 0.004101 |
| | | | C6···H6-C6 | 2.892 | -1.20 | 37.71 | 0.004471 |
| | | | C7···Br5 | 3.983 | -0.88 | 27.71 | 0.003285 |
| M1···M11 | 5.07 | -0.88 | Br5···Br5 | 3.656 | -0.88 | 100.00 | 0.006668 |
| M1···M12 | 9.18 | -1.28 | O1···H8-C8 | 2.970 | -1.28 | 100.00 | 0.0035 |
| M1···M13 | 32.59 | -7.24 | C7···C9 | 3.521 | -1.51 | 20.87 | 0.005594 |
| | | | C6···C4 | 3.519 | -1.47 | 20.26 | 0.005431 |
| | | | Br5···Br5 | 4.001 | -1.31 | 18.10 | 0.00485 |
| | | | Br5···C3 | 3.583 | -1.53 | 21.10 | 0.005655 |
| | | | C4···N2 | 3.364 | -1.42 | 19.67 | 0.005271 |
| M1···M14 | 9.18 | -1.28 | C8-H8···O1 | 2.970 | -1.28 | 100.00 | 0.003504 |
| M1···M15 | 13.47 | -3.19 | C9···O1 | 3.543 | -0.87 | 27.45 | 0.003153 |
| | | | N2···N2 | 3.245 | -1.44 | 45.08 | 0.005178 |
| | | | O1···C9 | 3.544 | -0.88 | 27.47 | 0.003155 |

**a:** ToposPro; **b:** (GM1···Mn = EM1···Mn – 2*EM1) corrected by BSSE; **c:** QTAIM (MultiWFN); **d:** Ec = GCM1···Mn * Contribution percentage.

**Table S3:** Pair of molecules M1···Mn, contact area, interaction energy, interaction, interatomic distance, contact energy, contribution percentage and electron density for the supramolecular cluster of *(E)*-*m*-Cl.

| Dimer | Contact Area[a] (Å$^2$) | Interaction Energy[b] (kcal.mol-1) | Interaction[c] | Interatomic distance (Å) | Contact energy[d] (kcal.mol-1) | Contribution percentage (%) | $\rho_{INT}$[c] (u.a.) |
|---|---|---|---|---|---|---|---|
| M1···M2 | 13.42 | -4.84 | O1-H1···N2 | 1.957 | -4.06 | 83.845 | 0.027316 |
| | | | O1···H5-C5 | 2.739 | -0.78 | 16.155 | 0.005263 |
| M1···M3 | 17.71 | -3.34 | O1···H3-C3 | 2.658 | -1.67 | 49.946 | 0.005581 |
| | | | C3-H3···O1 | 2.659 | -1.67 | 50.054 | 0.005593 |
| M1···M4 | 14.29 | -1.86 | C8-H8···Cl6 | 3.040 | -1.86 | 100.00 | 0.005632 |
| M1···M5 | 17.72 | -3.05 | Cl6···C8 | 3.767 | -1.01 | 33.036 | 0.003893 |
| | | | C7-H7···C6 | 3.018 | -1.02 | 33.529 | 0.003951 |
| | | | C7-H7···Cl6 | 3.113 | -1.02 | 33.435 | 0.00394 |
| M1···M6 | 4.60 | -0.19 | Cl6···Cl6 | 3.431 | -0.19 | 100.00 | 0.006044 |
| M1···M7 | 14.29 | -1.86 | Cl6···H8-C8 | 3.040 | -1.86 | 100.00 | 0.005639 |
| M1···M8 | 31.59 | -6.13 | Cl6···C5 | 3.639 | -1.16 | 18.91 | 0.004338 |
| | | | C7···C4 | 3.259 | -1.86 | 30.40 | 0.006972 |
| | | | C5···N2 | 3.305 | -1.67 | 27.25 | 0.00625 |
| | | | C4···O1 | 3.300 | -1.44 | 23.44 | 0.005377 |
| M1···M9 | 4.79 | -0.94 | C8-H8···Cl6 | 3.341 | -0.94 | 100.00 | 0.002246 |
| M1···M10 | 13.42 | -3.94 | C9-H9···O1 | 2.823 | -1.47 | 37.24 | 0.005344 |
| | | | C3-H3···H3-C3 | 2.893 | -1.01 | 25.54 | 0.003665 |
| | | | O1···H9-C9 | 2.823 | -1.47 | 37.22 | 0.00534 |
| M1···M11 | 19.30 | -4.86 | C5-H5···O1 | 2.739 | -0.78 | 16.11 | 0.005264 |
| | | | N2···H1-O1 | 1.956 | -4.07 | 83.89 | 0.027403 |
| M1···M12 | 4.79 | -0.94 | Cl6···H8-C8 | 3,341 | -0.94 | 100.00 | 0.002249 |
| M1···M13 | 31.59 | -6.13 | O1···C4 | 3.300 | -1.44 | 23.55 | 0.005377 |
| | | | N2···C5 | 3.305 | -1.68 | 27.35 | 0.006244 |
| | | | C4···C7 | 3.258 | -1.85 | 30.10 | 0.006871 |
| | | | C5···Cl6 | 3.639 | -1.17 | 19.00 | 0.004338 |
| M1···M14 | 17.72 | -3.05 | Cl6···H7-C7 | 3.113 | -1.02 | 33.41 | 0.003938 |
| | | | C6···H7-C7 | 3,018 | -1.02 | 33.50 | 0.003949 |
| | | | C8···Cl6 | 3.767 | -1.01 | 33.09 | 0.003901 |
| M1···M15 | 1.51 | -0.09 | C8-H8···H8-C8 | 4.267 | -0.09 | 100.00 | 0.000076 |

**a:** ToposPro; **b:** (GM1···Mn = EM1···Mn – 2*EM1) corrected by BSSE; **c:** QTAIM (MultiWFN); **d:** Ec = GCM1···Mn * Contribution percentage.

**Table S4:** Pair of molecules M1···Mn, contact area, interaction energy, interaction, interatomic distance, contact energy, contribution percentage and electron density for the supramolecular cluster of *(E)*-*p*-F.

| Dimer | Contact Area[a] (Å$^2$) | Interaction Energy[b] (kcal.mol-1) | Interaction[c] | Interatomic distance (Å) | Contact energy[d] (kcal.mol-1) | Contribution percentage (%) | $\rho_{INT}$[c] (u.a.) |
|---|---|---|---|---|---|---|---|
| M1···M2 | 18.93 | -13.00 | N2···H1-O1 | 1.877 | -6.50 | 50.05 | 0.035219 |
| | | | O1-H1···N2 | 1.877 | -6.49 | 49.95 | 0.03515 |
| M1···M3 | 18.15 | -3.35 | O1···C3 | 3.177 | -1.12 | 33.43 | 0.005709 |
| | | | C3-H3···O1 | 2.498 | -1.65 | 49.13 | 0.008389 |
| | | | C5-H5···O1 | 3.049 | -0.58 | 17.43 | 0.002977 |
| M1···M4 | 9.85 | -2.22 | C5-H5···N3 | 3.370 | -0.62 | 27.98 | 0.001716 |
| | | | C5-H5···H9-C9 | 2.607 | -0.96 | 42.95 | 0.002634 |
| | | | C6-H6···C9 | 3.390 | -0.65 | 29.06 | 0.001782 |
| M1···M5 | 10.53 | -1.59 | C6-H6···F7 | 2.646 | -0.59 | 37.24 | 0.004221 |
| | | | F7···F7 | 3.254 | -0.41 | 25.52 | 0.002893 |
| | | | F7···H6-C6 | 2.646 | -0.59 | 37.24 | 0.004221 |
| M1···M6 | 10.82 | -1.65 | F7···H8-C8 | 2.936 | -0.67 | 59.48 | 0.005283 |
| | | | F7···C8 | 3.467 | -0.98 | 40.52 | 0.003599 |
| M1···M7 | 9.98 | -1.31 | C8-H8···H6-C6 | 2.545 | -0.78 | 59.13 | 0.002989 |
| | | | C9-H9···C6 | 3.322 | -0.54 | 40.87 | 0.002066 |
| M1···M8 | 26.27 | -5.15 | N2···O1 | 3.498 | -1.10 | 21.33 | 0.002986 |
| | | | C9···C4 | 3.429 | -2.04 | 39.65 | 0.005549 |
| | | | C7···C6 | 3.431 | -2.01 | 39.02 | 0.005461 |
| M1···M9 | 18.15 | -3.36 | O1···H5-C5 | 3.049 | -0.58 | 17.43 | 0.002977 |
| | | | O1···H3-C3 | 2.498 | -1.65 | 49.18 | 0.008398 |
| | | | C3···O1 | 3.178 | -1.12 | 33.39 | 0.005702 |
| M1···M10 | 9.98 | -1.31 | C6-H6···H8-C8 | 2.544 | -0.78 | 59.08 | 0.002981 |
| | | | C6···H9-C9 | 3.323 | -0.54 | 40.92 | 0.002065 |
| M1···M11 | 2.18 | -0.26 | F7···F7 | 3.693 | -0.26 | 100.00 | 0.000881 |
| M1···M12 | 26.27 | -5.15 | O1···N2 | 3.498 | -1.10 | 21.33 | 0.002986 |
| | | | C4···C9 | 3.429 | -2.04 | 39.62 | 0.005546 |
| | | | C6···C7 | 3.430 | -2.01 | 39.05 | 0.005466 |
| M1···M13 | 10.82 | -1.65 | F7···H8-C8 | 2.579 | -0.98 | 59.45 | 0.005273 |
| | | | C8···F7 | 3.322 | -0.67 | 40.55 | 0.003596 |
| M1···M14 | 9.85 | -2.22 | C9···H6-C6 | 3.390 | -0.65 | 29.06 | 0.001784 |
| | | | C9-H9···H5-C5 | 2.608 | -0.96 | 42.98 | 0.002638 |
| | | | N2···H5-C5 | 3.370 | -0.62 | 27.96 | 0.001716 |
| M1···M15 | 8.77 | -1.36 | O1···O1 | 3.142 | -1.36 | 100.00 | 0.005929 |

**a:** ToposPro; **b:** (GM1···Mn = EM1···Mn – 2*EM1) corrected by BSSE; **c:** QTAIM (MultiWFN); **d:** Ec = GCM1···Mn * Contribution percentage.

**Table S5:** Pair of molecules M1···Mn, contact area, interaction energy, interaction, interatomic distance, contact energy, contribution percentage and electron density for the supramolecular cluster of *(Z)-p-Cl*.

| Dimer | Contact Area[a] (Å$^2$) | Interaction Energy[b] (kcal.mol-1) | Interaction[c] | Interatomic distance (Å) | Contact energy[d] (kcal.mol-1) | Contribution percentage (%) | $\rho_{INT}$[c] (u.a.) |
|---|---|---|---|---|---|---|---|
| M1···M2 | 8.11 | -3.99 | N2···H1-O1 | 1.986 | -3.99 | 100.00 | 0.025761 |
| M1···M3 | 11.94 | -2.07 | C3-H3···H6-C6 | 2.677 | -0.60 | 28.95 | 0.001965 |
| | | | C3-H3···Cl7 | 3.308 | -0.86 | 41.61 | 0.002824 |
| | | | C9-H9···Cl7 | 3.410 | -0.61 | 29.44 | 0.001998 |
| M1···M4 | 16.49 | -2.31 | C9-H9···H8-C8 | 2.871 | -0.49 | 21.19 | 0.002293 |
| | | | C9-H9···Cl7 | 3.294 | -0.69 | 29.72 | 0.003216 |
| | | | C8-H8···Cl7 | 2.991 | -1.13 | 49.09 | 0.005312 |
| M1···M5 | 16.49 | -2.30 | C8-H8···H9-C9 | 2.871 | -0.49 | 21.20 | 0.002297 |
| | | | Cl7···H9-C9 | 3.294 | -0.68 | 29.71 | 0.003219 |
| | | | Cl7···H8-C8 | 2.991 | -1.13 | 49.08 | 0.005317 |
| M1···M6 | 11.94 | -2.07 | C6-H6···H3-C3 | 2.677 | -0.60 | 28.95 | 0.001965 |
| | | | Cl7···H3-C3 | 3.308 | -0.86 | 41.61 | 0.002824 |
| | | | Cl7···H9-C9 | 3.410 | -0.61 | 29.44 | 0.001998 |
| M1···M7 | 17.91 | -3.16 | O1···C5 | 3.511 | -0.90 | 28.33 | 0.002957 |
| | | | C5-H5···N2 | 3.009 | -1.08 | 34.05 | 0.003554 |
| | | | C6-H6···O1 | 2.895 | -1.19 | 37.63 | 0.003928 |
| M1···M8 | 17.91 | -3.17 | O1···H6-C6 | 2.895 | -1.19 | 37.62 | 0.003928 |
| | | | N2···H5-C5 | 3.008 | -1.08 | 34.07 | 0.003557 |
| | | | C5···O1 | 3.511 | -0.90 | 28.31 | 0.002956 |
| M1···M9 | 27.79 | -5.73 | C3-H3···O1 | 2.904 | -2.06 | 35.86 | 0.005074 |
| | | | C9···C6 | 3.379 | -2.24 | 39.06 | 0.005528 |
| | | | C8-H8···Cl7 | 3.357 | -1.44 | 25.08 | 0.003549 |
| M1···M10 | 13.06 | -2.66 | C6···N2 | 3.395 | -1.54 | 57.79 | 0.004606 |
| | | | Cl7···C4 | 3.867 | -1.12 | 42.21 | 0.003364 |
| M1···M11 | 5.75 | -1.32 | Cl7···C8 | 3.661 | -1.32 | 100.00 | 0.003699 |
| M1···M12 | 5.75 | -1.32 | C8···Cl7 | 3.661 | -1.32 | 100.00 | 0.003696 |
| M1···M13 | 27.79 | -5.73 | O1···H3-C3 | 2.904 | -2.06 | 35.86 | 0.005074 |
| | | | C6···C9 | 3.379 | -2.24 | 39.06 | 0.005528 |
| | | | Cl7···H8-C8 | 3.357 | -1.44 | 25.08 | 0.003549 |
| M1···M14 | 16.49 | -2.66 | C9-H9···H8-C8 | 2.871 | -0.56 | 21.19 | 0.002293 |
| | | | C9-H9···Cl7 | 3.294 | -0.79 | 29.72 | 0.003216 |
| | | | C8-H8···Cl7 | 2.991 | -1.30 | 49.09 | 0.005312 |
| M1···M15 | 8.11 | -3.99 | O1-H1···N2 | 1.986 | -3.99 | 100.00 | 0.025737 |

**a:** ToposPro; **b:** (GM1···Mn = EM1···Mn – 2*EM1) corrected by BSSE; **c:** QTAIM (MultiWFN); **d:** Ec = GCM1···Mn * Contribution percentage.

**Table S6:** Pair of molecules M1···Mn, contact area, interaction energy, interaction, interatomic distance, contact energy, contribution percentage and electron density for the supramolecular cluster of *(E)-p-Br*.

| Dimer | Contact Area[a] (Å$^2$) | Interaction Energy[b] (kcal.mol-1) | Interaction[c] | Interatomic distance (Å) | Contact energy[d] (kcal.mol-1) | Contribution percentage (%) | $\rho_{INT}$[c] (u.a.) |
|---|---|---|---|---|---|---|---|
| M1···M2 | 22.37 | -9.62 | N2···H1-O1 | 1.942 | -4.81 | 50.00 | 0.029396 |
| | | | O1-H1···N2 | 1.942 | -4.81 | 50.00 | 0.029396 |
| M1···M3 | 18.21 | -3.15 | O1···H8-C8 | 2.622 | -1.20 | 38.07 | 0.005959 |
| | | | C3-H3···Br7 | 3.180 | -0.99 | 31.46 | 0.004932 |
| | | | C5-H5···Br7 | 3.122 | -0.96 | 30.47 | 0.004777 |
| M1···M4 | 16.14 | -2.83 | C6-H6···C6 | 3.158 | -0.67 | 23.58 | 0.002705 |
| | | | Br7···H6-C6 | 3.251 | -1.11 | 39.36 | 0.004516 |
| | | | Br7···H6-C6 | 3.282 | -1.05 | 37.05 | 0.00425 |
| M1···M5 | 4.11 | -0.95 | Br7···Br7 | 3.638 | -0.95 | 100.00 | 0.007443 |
| M1···M6 | 18.21 | -3.15 | C8-H8···O1 | 2.622 | -1.20 | 38.03 | 0.005959 |
| | | | Br7···H3-C3 | 3.181 | -0.99 | 31.48 | 0.004932 |
| | | | Br7···H5-C5 | 3.122 | -0.96 | 30.49 | 0.004777 |
| M1···M7 | 2.24 | -0.45 | N2···H8-C8 | 5.128 | -0.12 | 25.61 | 0.000042 |
| | | | C9-H9···H9-C9 | 4.067 | -0.22 | 48.78 | 0.00008 |
| | | | C8-H8···N2 | 5.128 | -0.12 | 25.61 | 0.000042 |
| M1···M8 | 16.14 | -2.83 | C6···H6-C6 | 3.158 | -0.67 | 23.60 | 0.002707 |
| | | | C6-H6···Br7 | 3.251 | -1.11 | 39.36 | 0.004515 |
| | | | C5-H5···Br7 | 3.282 | -1.05 | 37.05 | 0.00425 |
| M1···M9 | 4.11 | -0.95 | Br7···Br7 | 3.639 | -0.95 | 100.00 | 0.007442 |
| M1···M10 | 24.58 | -6.28 | O1···C4 | 3.483 | -1.19 | 18.87 | 0.003756 |
| | | | N2···C9 | 3.474 | -1.40 | 22.21 | 0.004421 |
| | | | C3···C6 | 3.695 | -1.08 | 17.23 | 0.00343 |
| | | | C4···C7 | 3.664 | -1.09 | 17.39 | 0.003461 |
| | | | C8···Br7 | 3.797 | -1.53 | 24.30 | 0.004837 |
| M1···M11 | 19.40 | -3.07 | Br7···H5-C5 | 3.460 | -1.19 | 38.85 | 0.003277 |
| | | | C8-H8···H5-C5 | 2.892 | -0.99 | 32.32 | 0.002726 |
| | | | C8···H3-C3 | 3.273 | -0.88 | 28.82 | 0.002431 |
| M1···M12 | 3.86 | 0.12 | O1···O1 | 3.463 | 0.12 | 100.00 | 0.002431 |
| M1···M13 | 21.36 | -4.11 | C9···O1 | 3.401 | -1.64 | 39.89 | 0.004448 |
| | | | N2···N2 | 3.796 | -0.82 | 19.94 | 0.002224 |
| | | | O1···C9 | 3.401 | -1.65 | 40.17 | 0.00448 |
| M1···M14 | 24.58 | -6.28 | C4···O1 | 3.483 | -1.19 | 18.87 | 0.003756 |
| | | | C9···N2 | 3.474 | -1.40 | 22.21 | 0.004421 |
| | | | C6···C3 | 3.695 | -1.08 | 17.23 | 0.00343 |
| | | | C7···C4 | 3.664 | -1.09 | 17.39 | 0.003461 |
| | | | Br7···C8 | 3.797 | -1.53 | 24.30 | 0.004837 |
| M1···M15 | 19.40 | -3.07 | C5-H5···Br7 | 3.460 | -1.19 | 38.85 | 0.003277 |
| | | | C5-H5···H8-C8 | 2.892 | -0.99 | 32.32 | 0.002726 |
| | | | C3-H3···C8 | 3.273 | -0.88 | 28.82 | 0.002431 |

**a:** ToposPro; **b:** (GM1···Mn = EM1···Mn – 2*EM1) corrected by BSSE; **c:** QTAIM (MultiWFN); **d:** Ec = GCM1···Mn * Contribution percentage.

**Table S7:** Pair of molecules M1···Mn, contact area, interaction energy, interaction, interatomic distance, contact energy, contribution percentage and electron density for the supramolecular cluster of *(Z)-p-Br*.

| Dimer | Contact Area[a] (Å$^2$) | Interaction Energy[b] (kcal.mol-1) | Interaction[c] | Interatomic distance (Å) | Contact energy[d] (kcal.mol-1) | Contribution percentage (%) | $\rho_{INT}$[c] (u.a.) |
|---|---|---|---|---|---|---|---|
| M1···M2 | 7.73 | -4.48 | N2···H1-O1 | 1.975 | -4.48 | 100.00 | 0.026306 |
| M1···M3 | 11.82 | -2.33 | C3-H3···Br7 | 3.323 | -1.29 | 55.317 | 0.003657 |
| | | | C9-H9···Br7 | 3.343 | -1.04 | 44.683 | 0.002954 |
| M1···M4 | 15.41 | -2.25 | C9-H9···Br7 | 3.373 | -0.92 | 40.793 | 0.003613 |
| | | | C8-H8···Br7 | 3.132 | -1.33 | 59.207 | 0.005244 |
| M1···M5 | 15.41 | -2.25 | Br7···H9-C9 | 3.373 | -0.92 | 40.826 | 0.003618 |
| | | | Br7···H8-C8 | 3.132 | -1.33 | 59.174 | 0.005244 |
| M1···M6 | 11.82 | -2.33 | Br7···H3-C3 | 3.323 | -1.29 | 55.317 | 0.003657 |
| | | | Br7···H9-C9 | 3.343 | -1.04 | 44.683 | 0.002954 |
| M1···M7 | 18.63 | -3.46 | O1···C5 | 3.479 | -1.01 | 29.092 | 0.003212 |
| | | | C5-H5···N2 | 3.068 | -1.12 | 32.379 | 0.003575 |
| | | | C6-H6···O1 | 2.839 | -1.33 | 38.529 | 0.004254 |
| M1···M8 | 13.34 | -2.89 | C6···N2 | 3.399 | -1.47 | 50.853 | 0.004502 |
| | | | Br7···C4 | 3.837 | -1.42 | 49.147 | 0.004351 |
| M1···M9 | 28.07 | -6.32 | C8-H8···Br7 | 3.422 | -1.30 | 20.64 | 0.003927 |
| | | | C9···C6 | 3.311 | -2.04 | 32.22 | 0.006132 |
| | | | C3-H3···C5 | 3.248 | -0.93 | 14.76 | 0.002808 |
| | | | C3-H3···O1 | 2.734 | -2.05 | 32.38 | 0.006162 |
| M1···M10 | 18.63 | -3.46 | O1···H6-C6 | 2.839 | -1.33 | 38.521 | 0.00425 |
| | | | N2···H5-C5 | 3.068 | -1.12 | 32.394 | 0.003574 |
| | | | C5···O1 | 3.479 | -1.01 | 29.085 | 0.003209 |
| M1···M11 | 13.34 | -2.89 | C4···Br7 | 3.837 | -1.42 | 49.147 | 0.004351 |
| | | | N2···C6 | 3.399 | -1.47 | 50.853 | 0.004502 |
| M1···M12 | 7.73 | -4.47 | O1-H1···N2 | 1.975 | -4.47 | 100.00 | 0.026285 |
| M1···M13 | 28.07 | -6.32 | Br7···H8-C8 | 3.422 | -1.30 | 20.637 | 0.003927 |
| | | | C6···C9 | 3.311 | -2.04 | 32.224 | 0.006132 |
| | | | C5···H3-C3 | 3.248 | -0.93 | 14.756 | 0.002808 |
| | | | O1···H3-C3 | 2.734 | -2.05 | 32.382 | 0.006162 |
| M1···M14 | 6.68 | -1.73 | C8-H8···Br7 | 3.618 | -1.10 | 63.283 | 0.004993 |
| | | | Br7···Br7 | 4.114 | -0.64 | 36.717 | 0.002897 |
| M1···M15 | 6.68 | -1.73 | Br7···C8 | 3.618 | -1.10 | 63.283 | 0.004993 |
| | | | Br7···Br7 | 4.114 | -0.64 | 36.717 | 0.002897 |

**a:** ToposPro; **b:** (GM1···Mn = EM1···Mn – 2*EM1) corrected by BSSE; **c:** QTAIM (MultiWFN); **d:** Ec = GCM1···Mn * Contribution percentage.

**Table S8:** Pair of molecules M1···Mn, contact area, interaction energy, interaction, interatomic distance, contact energy, contribution percentage and electron density for the supramolecular cluster of *(E)-p-I*.

| Dimer | Contact Area[a] (Å$^2$) | Interaction Energy[b] (kcal.mol-1) | Interaction[c] | Interatomic distance (Å) | Contact energy[d] (kcal.mol-1) | Contribution percentage (%) | $\rho_{INT}$[c] (u.a.) |
|---|---|---|---|---|---|---|---|
| M1···M2 | 20.58 | -7.67 | N2···H1-O1 | 2.084 | -3.84 | 50.00 | 0.020953 |
| | | | O1-H1···N2 | 2.084 | -3.84 | 50.00 | 0.020953 |
| M1···M3 | 5.31 | -1.38 | O1···I7 | 3.447 | -1.38 | 100.00 | 0.006093 |
| M1···M4 | 18.40 | -2.37 | O1···H8-C8 | 3.305 | -0.36 | 15.03 | 0.001481 |
| | | | C3-H3···H8-C8 | 2.678 | -0.51 | 21.72 | 0.00214 |
| | | | C5-H5···I7 | 3.174 | -1.50 | 63.25 | 0.006232 |
| M1···M5 | 18.22 | -2.97 | C6-H6···I7 | 3.527 | -0.91 | 30.70 | 0.003507 |
| | | | C6-H6···H6-C6 | 2.367 | -1.15 | 38.65 | 0.004415 |
| | | | I7···H6-C6 | 3.526 | -0.91 | 30.65 | 0.003501 |
| M1···M6 | 5.31 | -1.38 | I7···O1 | 3.447 | -1.38 | 100.00 | 0.006094 |
| M1···M7 | 18.40 | -2.37 | C8-H8···O1 | 3.305 | -0.36 | 15.05 | 0.00148 |
| | | | C8-H8···H3-C3 | 2.679 | -0.51 | 21.69 | 0.002133 |
| | | | I7···H5-C5 | 3.175 | -1.50 | 63.27 | 0.006223 |
| M1···M8 | 17.30 | -3.37 | N2···H6-C6 | 2.870 | -1.60 | 47.44 | 0.004798 |
| | | | C9-H9···I7 | 3.299 | -1.77 | 52.56 | 0.005316 |
| M1···M9 | 2.95 | -0.46 | C3-H3···H3-C3 | 4.198 | -0.46 | 100.00 | 0.000081 |
| M1···M10 | 43.30 | -7.37 | O1···I7 | 4.015 | -1.34 | 18.20 | 0.003038 |
| | | | C4···C5 | 3.550 | -2.35 | 31.82 | 0.005312 |
| | | | C5···C4 | 3.550 | -2.34 | 31.80 | 0.005309 |
| | | | I7···O1 | 4.016 | -1.34 | 18.18 | 0.003035 |
| M1···M11 | 2.53 | -1.18 | I7···I7 | 4.151 | -1.18 | 100.00 | 0.004946 |
| M1···M12 | 17.30 | -3.37 | C6-H6···N2 | 2.871 | -1.60 | 47.40 | 0.004792 |
| | | | I7···H9-C9 | 3.299 | -1.77 | 52.60 | 0.005317 |
| M1···M13 | 19.27 | -3.84 | C9-H9···I7 | 3.499 | -1.16 | 30.18 | 0.004018 |
| | | | C8-H8···H8-C8 | 2.599 | -1.52 | 39.57 | 0.005268 |
| | | | I7···H9-C9 | 3.498 | -1.16 | 30.24 | 0.004026 |
| M1···M14 | 31.33 | -6.69 | O1···C7 | 3.455 | -1.52 | 22.66 | 0.004413 |
| | | | N2···C8 | 4.043 | -1.83 | 27.35 | 0.005326 |
| | | | C8···N2 | 4.053 | -1.83 | 27.35 | 0.005326 |
| | | | C7···O1 | 3.454 | -1.52 | 22.64 | 0.00441 |

**a:** ToposPro; **b:** (GM1···Mn = EM1···Mn – 2*EM1) corrected by BSSE; **c:** QTAIM (MultiWFN); **d:** Ec = GCM1···Mn * Contribution percentage.

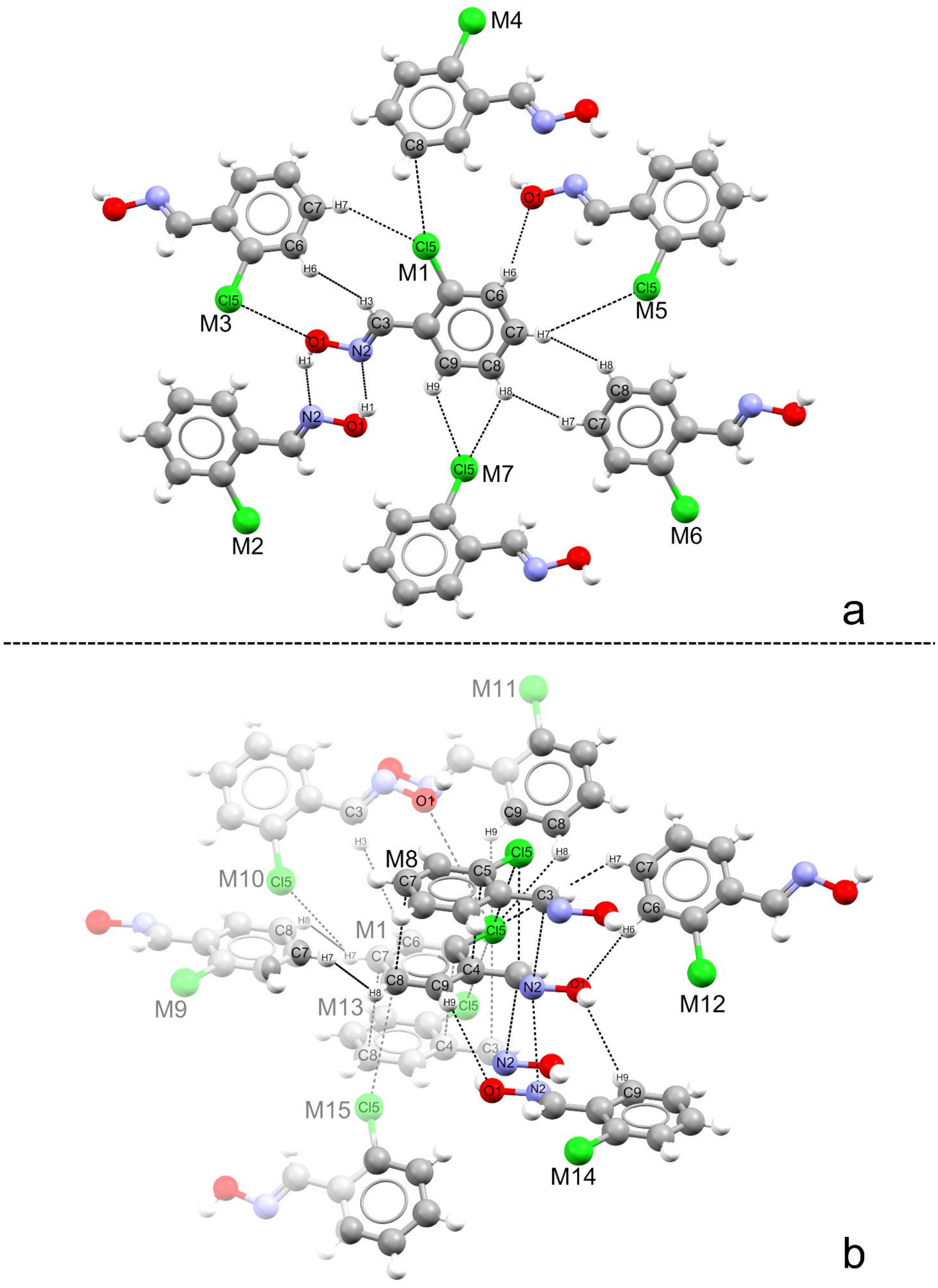


**Figure S3.** Planes of the first coordination sphere for *(E)-o*-Cl **a)** in the same plane and **b)** in upper and lower layers**.**

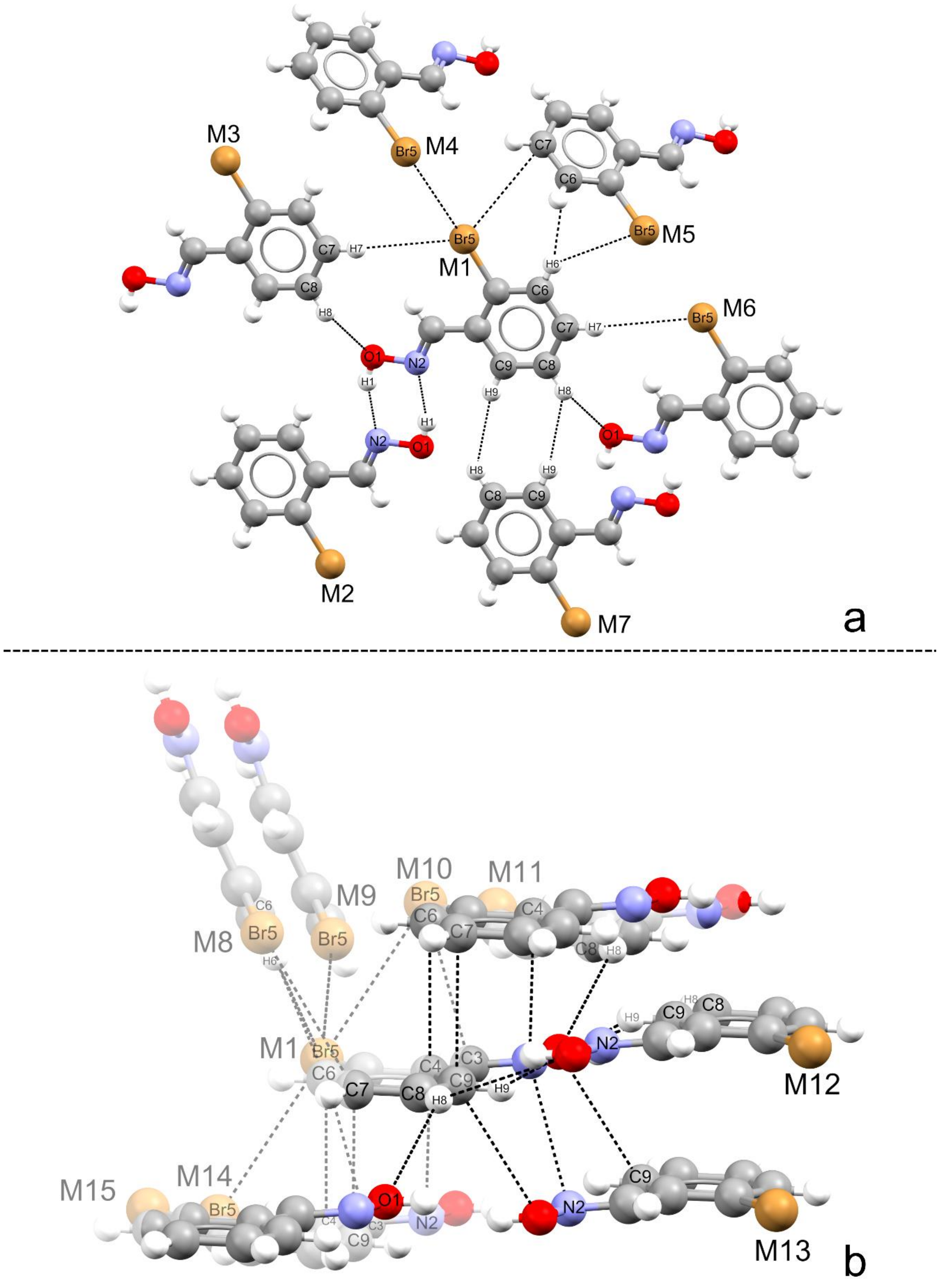


**Figure S4.** Planes of the first coordination sphere for *(E)-o*-Br **a)** in the same plane and **b)** in upper and lower layers**.**

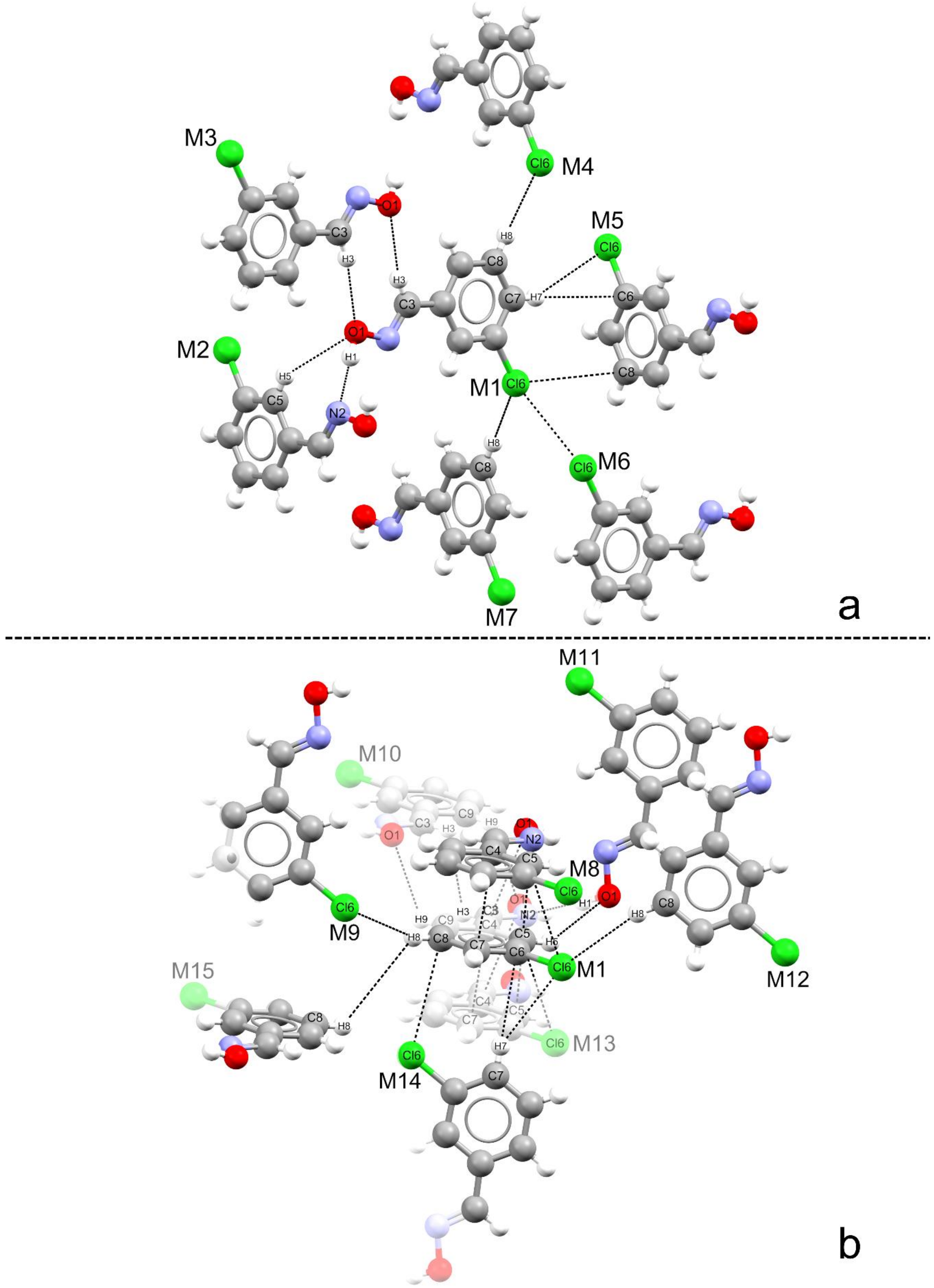


**Figure S5.** Planes of the first coordination sphere for *(E)-m*-Cl **a)** in the same plane and **b)** in upper and lower layers**.**

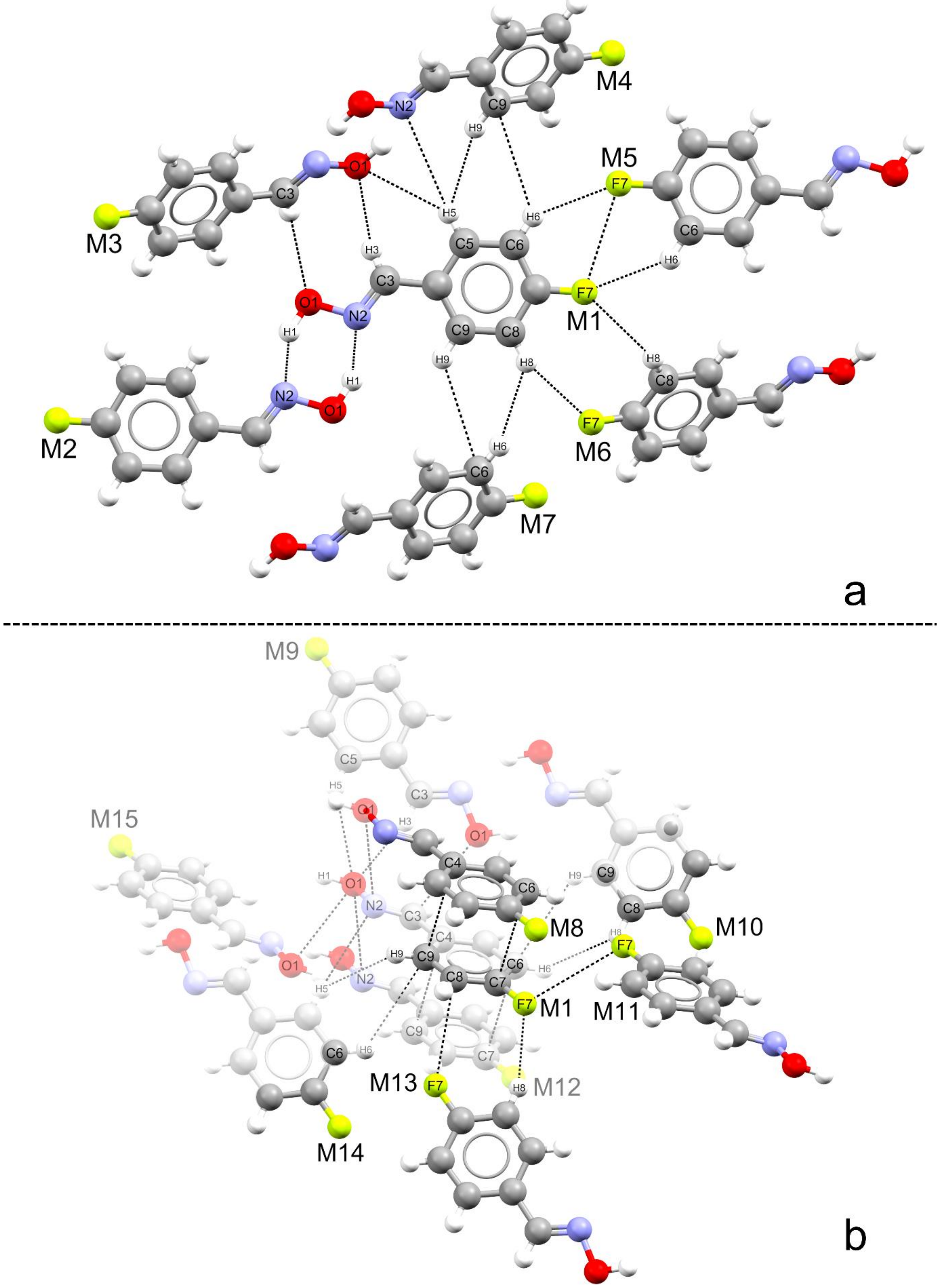


**Figure S6.** Planes of the first coordination sphere for *(E)-p*-F **a)** in the same plane and **b)** in upper and lower layers**.**

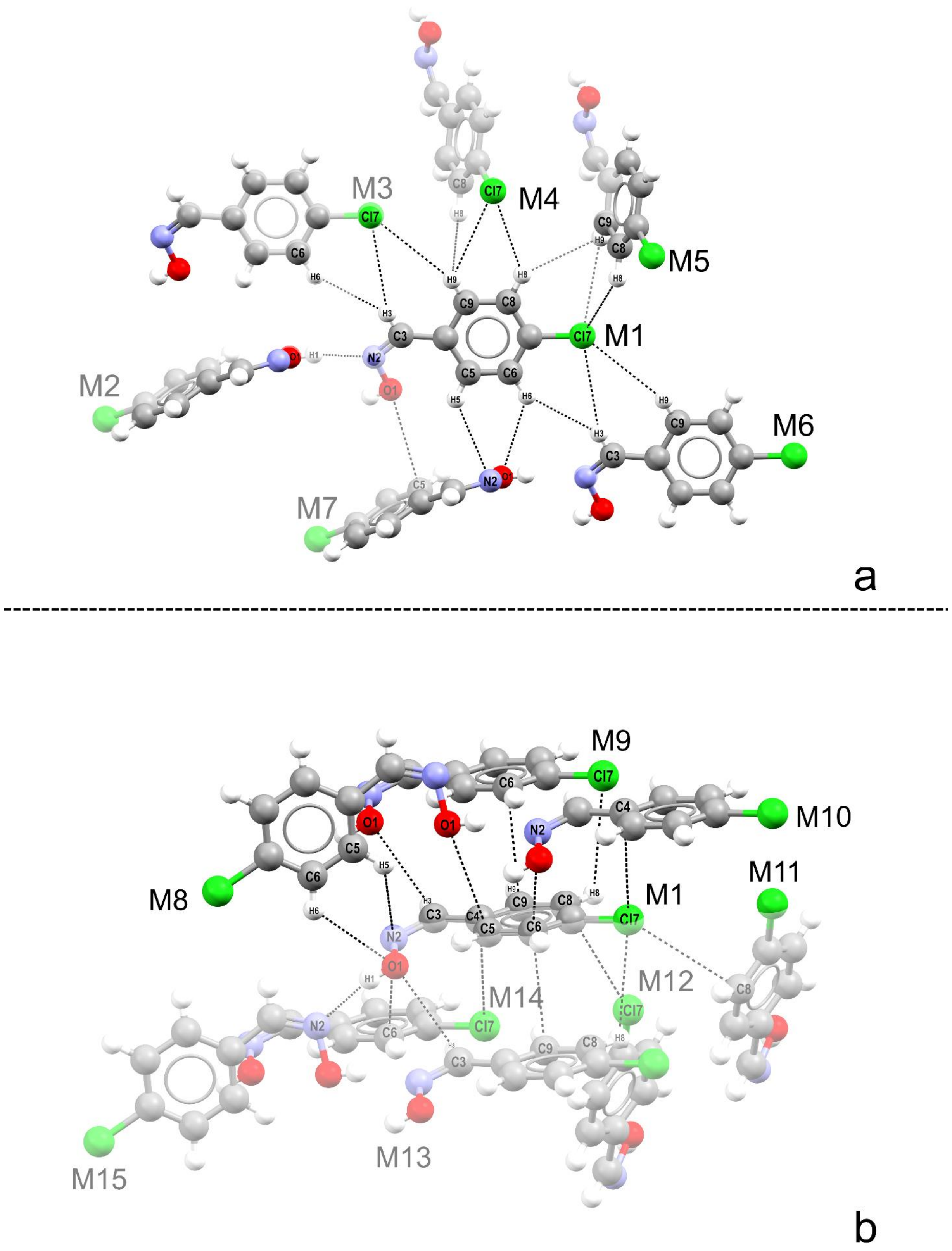


**Figure S7.** Planes of the first coordination sphere for *(Z)*-*p*-Cl **a)** in the same plane and **b)** in upper and lower layers**.**

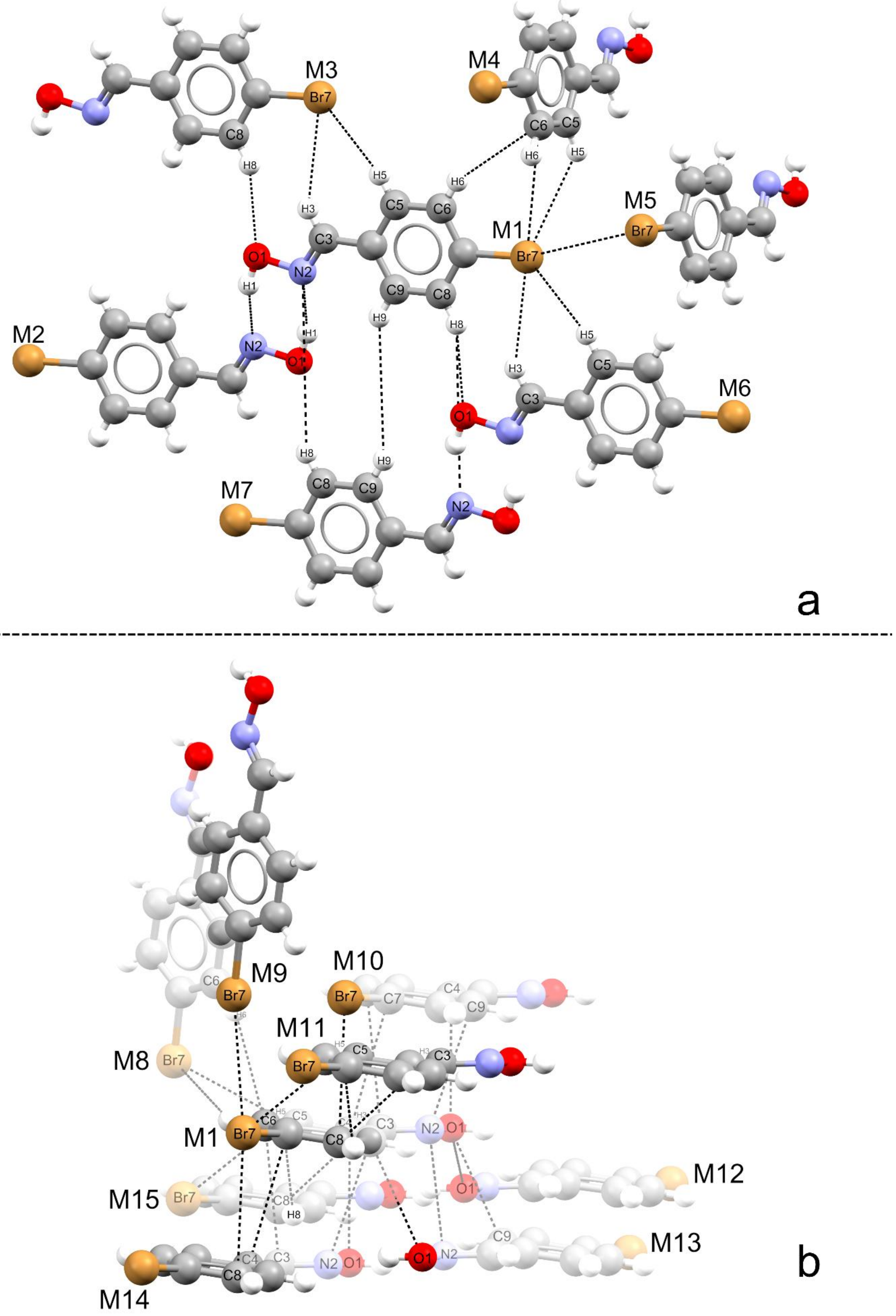


**Figure S8.** Planes of the first coordination sphere for *(E)*-*p*-Br **a)** in the same plane and **b)** in upper and lower layers**.**

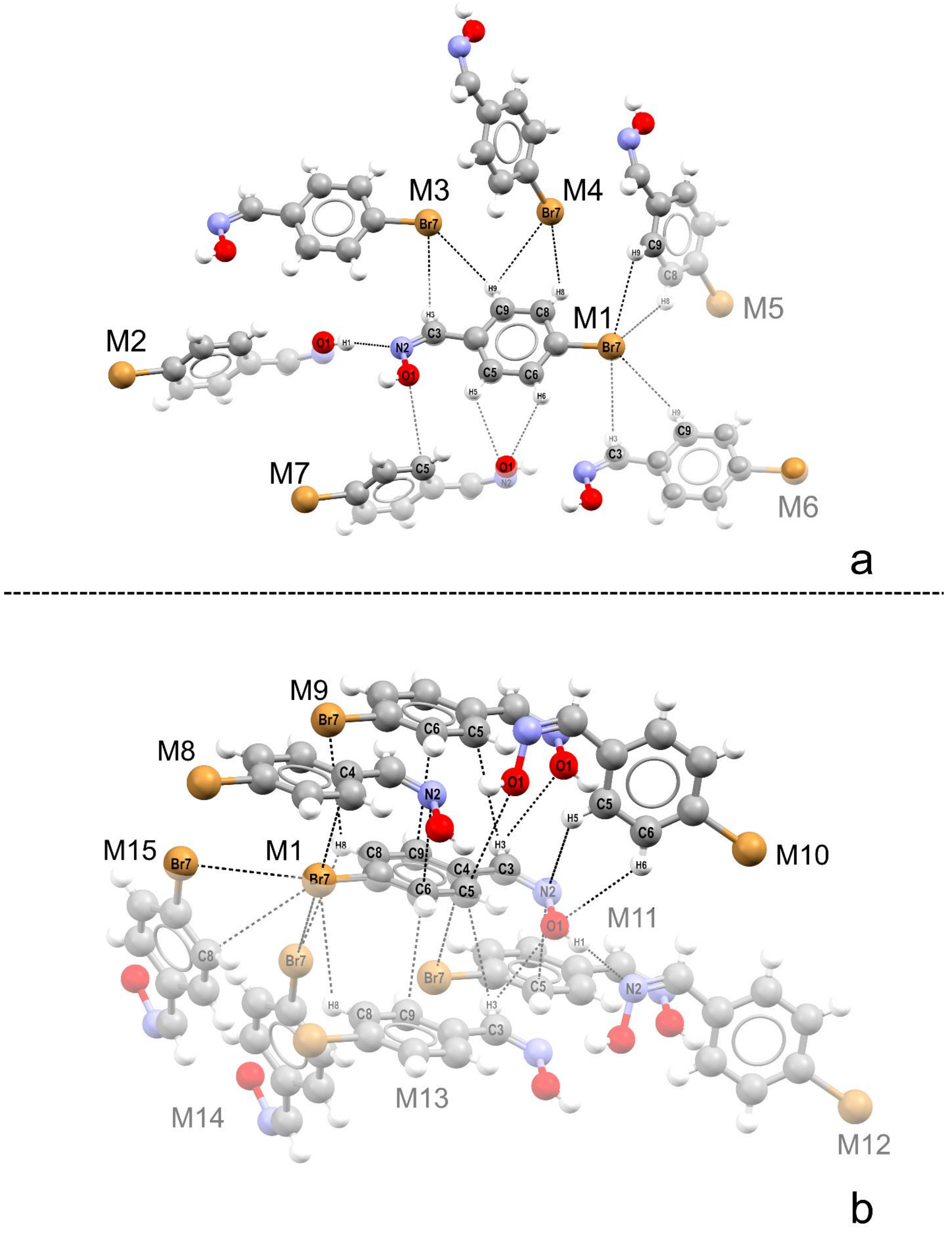


**Figure S9.** Planes of the first coordination sphere for *(Z)*-*p*-Br **a)** in the same plane and **b)** in upper and lower layers**.**

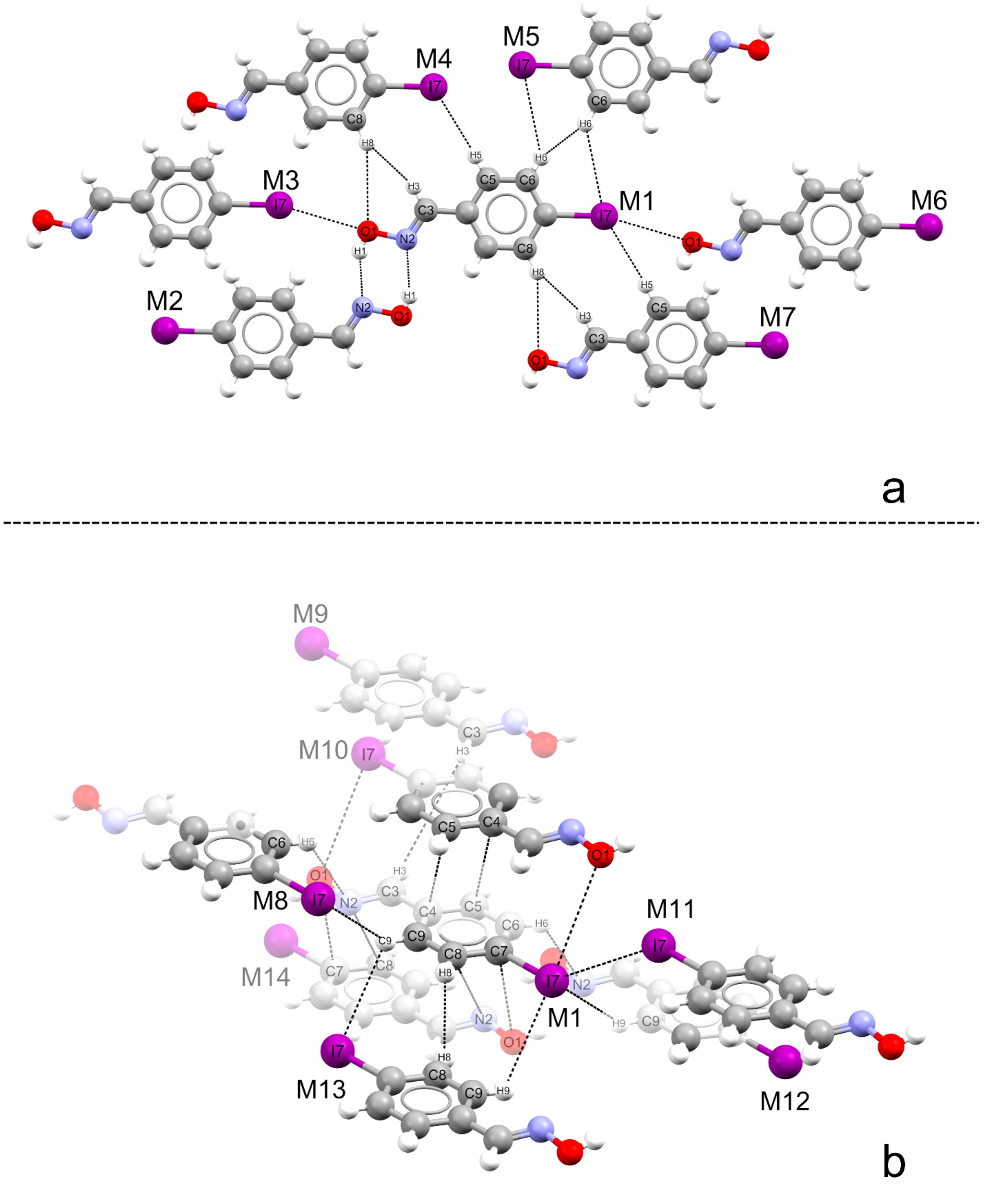


**Figure S10.** Planes of the first coordination sphere for *(E)*-*p*-I **a)** in the same plane and **b)** in upper and lower layers**.**

It was found that the O–H···N interaction stood out as the most stabilizing interaction of the first coordination sphere in 6 of the 9 compounds studied. However, in three specific cases, the most stabilizing interaction did not come from the hydrogen bond, but from the sum of weaker and more numerous interactions, as is the case of π stacking, which reveals a large contact area (*(E)-m*-Cl, *(Z)-p*-Cl and *(Z)-p*-Br). These compounds, which present a large area in the highest-energy interaction, contribute to the trend observed not only in this study but also in other studies by our research group.[45,46] This trend relates the contact area and the stabilization energy. The relationship between less intense interaction energies (which do not present high stabilization energy values) and the contact area is directly proportional: the larger the surface area formed, the higher the stabilization energy of the approximation. **Figure S11** illustrates this type of analysis. In π stacking, (E)-p-Cl presented a contact area of 23.65 Å² and a stabilization energy of –6.05 kcal·mol⁻¹ (**Figure S11a**), while (Z)-p-Cl exhibited a slightly larger area of 27.79 Å², associated with an energy of –5.73 kcal·mol⁻¹ **(Figure S11b**). This comparison was also performed for dimers formed by O–H···N interactions. In the case of (E)-p-Cl, due to its spatial configuration, which allows the occurrence of two hydrogen bonds between the pair of molecules, resulting in a contact area of 22.39 Å² and a stabilization energy of –10.64 kcal·mol⁻¹ (**Figure S11c**). In (Z)-p-Cl, only one hydrogen bond occurs between the pair of molecules, due to the restriction of the geometric arrangement. This interaction results in an area of 8.11 Å² and a stabilization energy of –3.99 kcal·mol⁻¹ (**Figure S11d**).

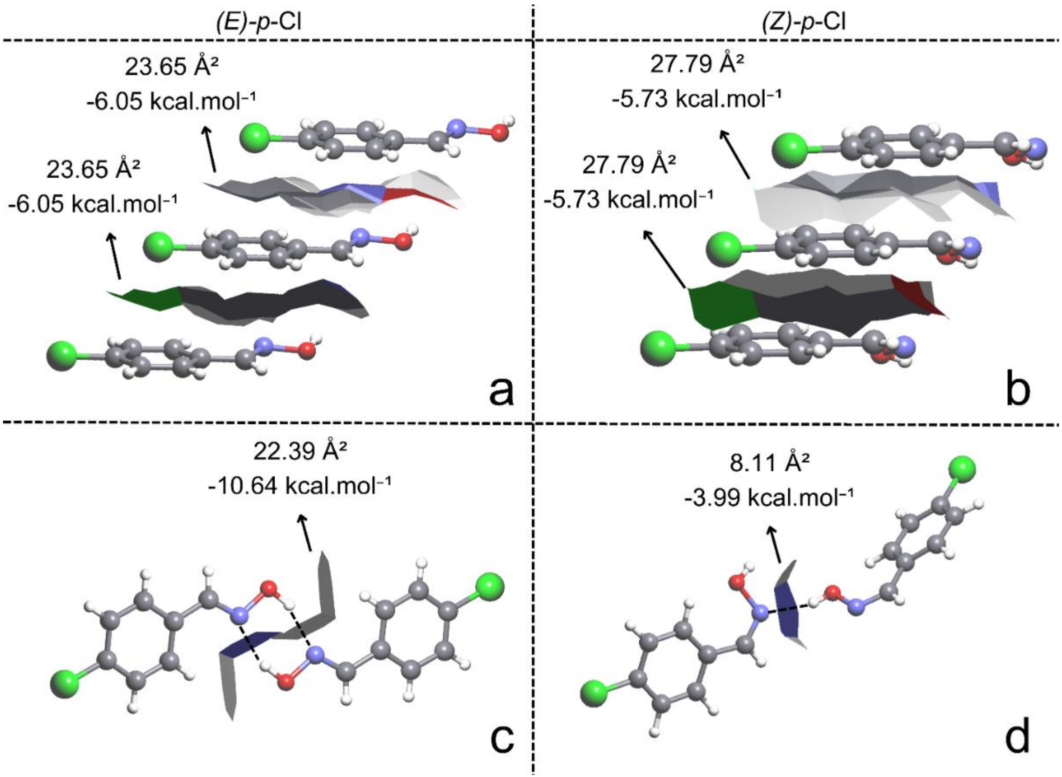


**Figure S11:** Comparison between the contact areas and stabilization energies of the configurations present in the first coordination sphere of the oximes *(E)-p*-Cl and *(Z)-p*-Cl. (a) π-stacking of the compound *(E)-p*-Cl. (b) π-stacking of the compound *(Z)-p*-Cl. (c) Pair of molecules M1···M2 of the oxime *(E)-p*-Cl (with O-H···N and C-H···O interactions). (d) Supramolecular dimer M1···M2 of the oxime *(Z)-p*-Cl (with O-H···N interactions).

After analyzing the data obtained, a significant number of occurrences of C-H···X interactions was observed for all compounds, reaching a value of 74, although the interaction class has a low stabilization energy (approximately 18.8%), compared to the 18 occurrences of O-H···N interactions representing a

total of 20.8% of energy contribution, as illustrated in the graph in **Figure S12**. This comparison reveals the strength of the intermolecular interactions. The π···π interaction is represented as C···C and the C-H···π interaction is represented as C-H···C, due to the QTAIM analysis. Some interactions, such as C-H···O, C-H···H-C, X···X, among others, are shown in **Figure S12** and play a fundamental role in the crystal packing of the structures.

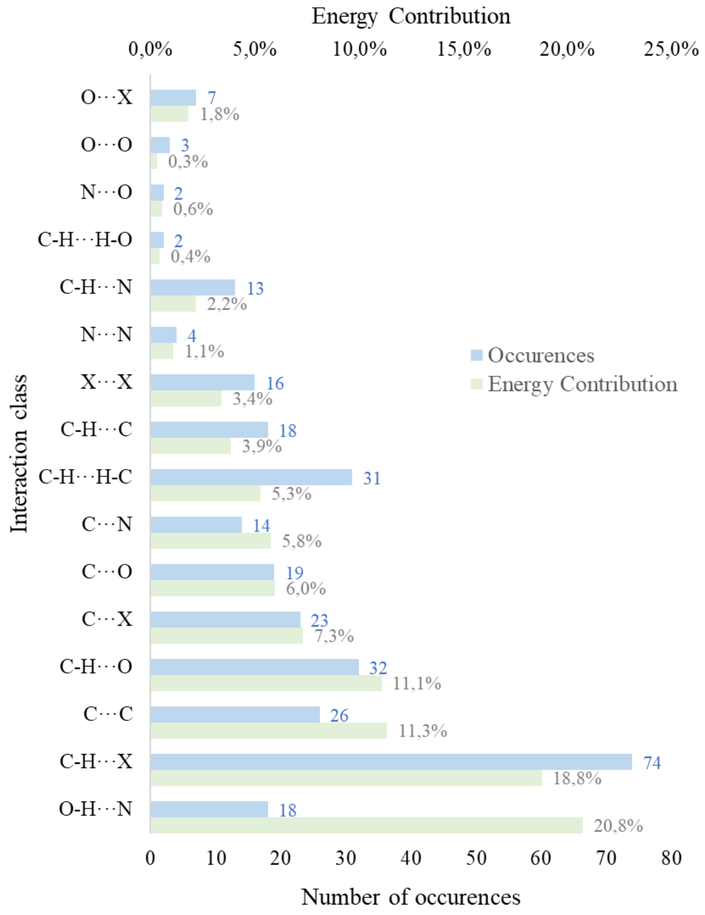


**Figure S12**: Number of occurrences and energetic contribution of each class of intermolecular interaction for all compounds.

To establish a reliable correlation between theoretical and experimental data, comparisons were made between compounds containing the chlorine atom in the three possible positions of the benzene ring and between compounds para-substituted by different halogens. In the first case, it is observed that in the experimental data the order of melting points is increasing (E)-m-Cl < (E)-o-Cl < (E)-p-Cl, a trend also observed for pairwise first-shell cohesive energy values. Statistical analysis confirmed this consistency, showing a high correlation coefficient ($R^2$ = 0.9855; **Figure S13**, green). For para-substituted compounds, the same increasing trend was observed for (E)-p-F < (E)-p-Cl < (E)-p-Br, both for melting points and pairwise first-shell cohesive energy, with $R^2$ = 0.971 (**Figure S13**, purple). However, the inclusion of compound (E)-p-I significantly alters this behavior: although its pairwise first-shell cohesive energy presents the highest value among the para-substituted compounds due to its larger atomic radius, its melting point decreases compared to the other oximes. This discrepancy drastically reduces the correlation coefficient ($R^2$ = 0.0094; Figure S13, pink) for the para-substituted compounds and also affects the overall analysis of compounds (E), where $R^2$ decreases from 0.8243 (without (E)-p-I, orange in **Figure S13**) to 0.5650 (with (E)-p-I included, yellow in **Figure S13**). Therefore, it was observed that (E)-p-I does not follow the same linear relationship between pairwise

first-shell cohesive energy and melting point observed for the other oximes, indicating that more factors are involved in crystal packing than intermolecular interactions. The iodine atom has a large volume and high polarizability, which intensifies the strength of its intermolecular interactions. However, the larger atomic radius also reduces packing efficiency due to strong steric hindrances, as evidenced by its MCN = 13 (lower than the others at 14). This can result in lower cohesion of the final crystal structure, also affecting the melting point. Furthermore, the unit cell of compound (E)-p-I has Z = 2, and the unit cells of the other para-substituted compounds have Z = 4, which influences the lower packing of compounds containing iodine, lowering the melting point.

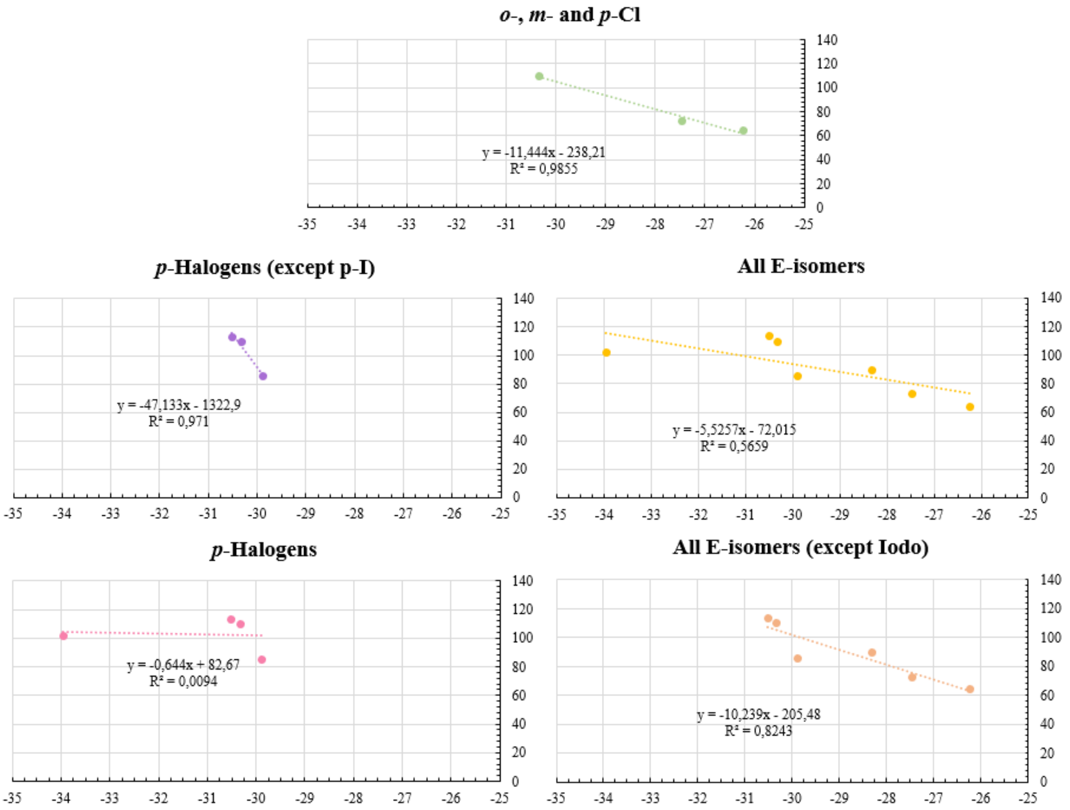


**Figure S13**: Linear correlations between pairwise first-shell cohesive energy and melting points of compounds.

**Table S9**: Pairwise first-shell cohesive energy and melting point of each compound.

| Compound | Pairwise first-shell cohesive energy (kcal.mol$^{-1}$) | Melting Point (°C) | Melting point references |
|---|---|---|---|
| *(E)-o*-Cl | -27.48 | 72 – 74 | 51 |
| *(E)-o*-Br | -28.32 | 90 | 52 |
| *(E)-m*-Cl | -26.25 | 64 – 65 | 53 |
| *(E)-p*-F | -29.90 | 85 – 86 | 53 |
| *(E)-p*-Cl | -30.34 | 110 | 47 |
| *(Z)-p*-Cl | -27.29 | 146 – 147 | 49 |
| *(E)-p*-Br | -30.52 | 113 – 114 | 51 |
| *(Z)-p*-Br | -27.93 | 109 – 110 | 54 |
| *(E)-p*-I | -33.97 | 101 – 103 | 7 |

Stage 1 of the crystallization mechanism for compound *(E)-o*-Cl begins with the interaction with the most stabilizing energy of the first coordination sphere, which is –7.53 kcal·mol$^{-1}$, referring to the O–H···N interaction. This interaction has an area of 20.54 Å$^2$ and is characterized by the formation of a supramolecular dimer, as can be seen in **Figure S14**, and it is from this dimer that the growth of stage 2 begins.

In stage 2, four hypotheses were proposed, named I, II, III, and IV. Hypothesis I has C-H···H-C interactions with an energy of -1.26 kcal·mol$^{-1}$ and an area of 11.55 Å$^2$ with growth along the *c*-axis. Hypothesis II reveals growth along the *b*-axis and *c*-axis from the interactions are C-H···O and C-H···Cl. These interactions have a stabilization energy of -3.78 kcal·mol$^{-1}$ and a contact area of 22.05 Å$^2$. Hypothesis III involves π stacking along the *a* axis, forming a supramolecular chain. This stacking results in an area of 83.50 Å$^2$ with a stabilization energy of -17.89 kcal mol$^{-1}$. Therefore, because it has the highest stabilization energy, hypothesis III should guide stage 2 of the mechanism, in which a supramolecular chain is formed (**Figure S14**). This type of interaction also occurs in the second growth stage in halogenated benzoic acids.[42]

In stage 3, there are three hypotheses that are visually identical to those presented previously (the only difference being a higher number of molecules than in the previous stage). Hypothesis I of the third stage, for example, has the same interactions as hypothesis I of the second stage, and so on. Therefore, only the stabilization energy and contact area data for each hypothesis in this stage will be presented. Hypothesis I reveals an energy of -6.96 kcal mol$^{-1}$ and an area of 60.97 Å$^2$. Hypothesis II presents a contact area of 97.76 Å$^2$, where the energy is -15.11 kcal mol$^{-1}$. In Hypothesis III, there is, again, π stacking, increasing the already formed chain. However, because this is a repetition of a hypothesis that has already occurred previously, this hypothesis will be referred to as an expansion of the previous stage. When considering a hypothesis such as expansion, the stabilization energy is assumed to be

doubled to ensure a fair comparison regarding the number of approaching molecules, since in hypothesis III the number of molecules moved is smaller than in hypothesis I. Therefore, the stabilization energy used in this stage competition is -37.29 kcal $mol^{-1}$, although the total area remains the same. Since hypothesis III has the highest energy among the other hypotheses, it should drive stage 3 of the mechanism, increasing the supramolecular chain to five molecules in one expansion stage (**Figure S14**).

In stage 4, the same situation as in stage 3 occurs; therefore, only the energies and areas will again be described. Hypothesis I presented a stabilization energy of -12.08 kcal $mol^{-1}$ with a contact area of 110.38 $Å^2$. Hypothesis II presents an area value equal to 173.46 $Å^2$ and stabilization energies of -32.98 kcal $mol^{-1}$. In Hypothesis III, another expansion occurs; therefore, the energy remains at -37.29 kcal $mol^{-1}$ and the area at 83.50 $Å^2$. Thus, Hypothesis III is thermodynamically favored and should once again drive growth along the *a*-axis, further increasing the previously formed supramolecular chain to seven molecules (**Figure S14**).

In stage 5, there are three hypotheses, this time distinct in order of increasing energy. Hypothesis I is the expansion of the previous stage, that is, growth in the direction of the *a* axis, with a stabilizing energy of -37.29 kcal $mol^{-1}$ and an area of 83.50 $Å^2$. Hypothesis II presents C-H···H-C and C-H···C interactions that, when added, result in a stabilization energy of -17.72 kcal $mol^{-1}$ and an area of 159.80 $Å^2$. Hypothesis III, on the other hand, involves growth along the *bc* plane, generating a contact area of 249.17 $Å^2$. Here, the contact area is significantly larger than the areas of the other hypotheses, and a fact observed when describing the proposed crystallization mechanisms was that, typically, the larger the contact area, the higher the stabilization energy, with the exception of expansions. The interactions that occur in this hypothesis are of the C-H···Cl and C-H···O type, resulting in an energy of -46.65 kcal $mol^{-1}$. This last hypothesis presented has the highest stabilization energy, therefore, it should guide stage 5, forming a supramolecular layer (**Figure S14**). Even though the structure formed in the fifth stage is already in three dimensions, the C-H···H-C interaction is still missing for all interactions of the first coordination sphere to be present in the crystal growth.

Thus, three hypotheses were investigated for stage 6 of the crystallization mechanism of benzoxime *(E)-o*-Cl. At this stage, there are, again, only three hypotheses. Hypothesis I presents growth along the *a* axis through the stacking of a central block with the block formed in the previous stage. The interactions of this hypothesis are C···C, Cl···Cl, C···Cl, and C···N and, when added together, result in an energy of -106.01 kcal·$mol^{-1}$ and an area of 503.82 $Å^2$. In hypothesis II, the interactions are of the C-H···Cl and C-H···O type, from a contact area of 747.50 $Å^2$. The growth of this hypothesis is along the *b* axis and the stabilization energy is -139.95 kcal·$mol^{-1}$. In hypothesis III, there is growth in the direction of the *c* axis, which results in an area of 1070.29 $Å^2$ formed by C-H···H-C and C-H···Cl interactions and the energy of this hypothesis is -174.54 kcal·$mol^{-1}$. Thus, hypothesis III is the one with the most stabilizing energy and should direct stage 6 of the proposed crystallization mechanism. Since all intermolecular interactions present in the first coordination sphere have already been described and there was growth along the three directions in the mechanism, the proposed crystallization of *(E)-o*-Cl is concluded with six stages (**Figure S14**).

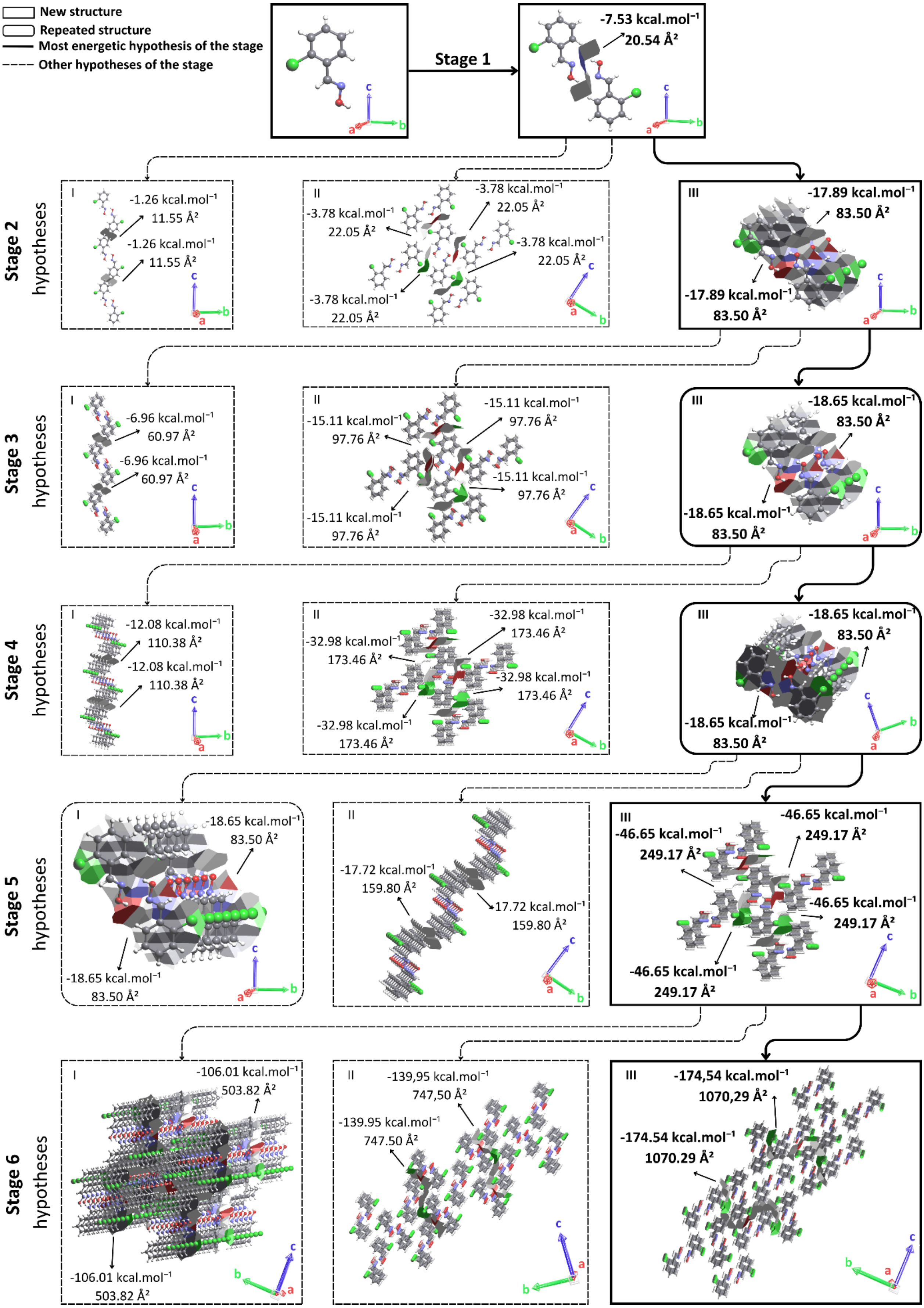


**Figure S14.** Crystallization mechanism proposal for *(E)*-*o*-Cl.

The first stage of the crystallization mechanism of *(E)-o*-Br is from the most stabilizing energy of the first coordination sphere and, when checking this value, it is found in the O-H···N interaction being -8.46 kcal·mol$^{-1}$ with a contact area of 19.53 Å$^2$. This higher energy forms a dimer, as represented in **Figure S15**, and it is from this dimer that the next stage follows.

In stage 2 of the proposed mechanism, four distinct hypotheses emerge. Hypothesis I presents growth along the *c* axis, driven by the single halogen bond Br···Br (via σ-hole) that results in an energy of -0.94 kcal·mol$^{-1}$ and an area of 5.07 Å$^2$. Hypothesis II presents growth that is between the *a* axis and the *c* axis, in which the stabilization energy is -3.17 kcal·mol$^{-1}$ due to interactions of the C-H···Br, C···Br and C-H···C type that form an area of 36.11 Å$^2$. In hypothesis III, the C-H···O and C-H···H-C contact points show growth in the *a* direction, with an energy of -4.43 kcal·mol$^{-1}$ and a contact area of 32.47 Å$^2$. In hypothesis IV, there is stacking that grows along the *b* axis, guided by π···π interactions (where the contact points are C···C, Br···Br, C···Br, and C···N). These interactions form a supramolecular chain with a surface area equal to 78.64 Å$^2$ and result in an energy value of -17.88 kcal·mol$^{-1}$ (**Figure S15**). When analyzing the hypotheses presented, it is clear that the one with the most stabilizing energy is hypothesis IV, therefore, the supramolecular chain formed by this hypothesis is the final structure of stage 2 and, therefore, it is from this chain that the growth of the next stage will occur.

In stage 3, there are four distinct hypotheses, however, similar to the hypotheses presented in the previous stage, the only difference being the number of molecules involved in each configuration, exactly the same case that occurred in the first stages of the proposed crystallization mechanism of the o-Cl oxime. In hypothesis I, the suggested growth is along the *c* axis, guided by the single Br···Br interaction that has an energy of -5.04 kcal·mol$^{-1}$ and a contact area of 25.33 Å$^2$. In hypothesis II, the C-H···Br, C···Br and C-H···C contact points suggest a growth distributed between the a and c axes, forming a surface with an area equal to 90.29 Å$^2$ with an energy of -17.23 kcal·mol$^{-1}$. Hypothesis III suggests a growth along the *a* axis which has a stabilization energy of -26.02 kcal·mol$^{-1}$ due mainly to the C-H···O and C-H···Br interactions which form a contact area of 154.00 Å$^2$. In the last hypothesis of this stage, hypothesis IV, there is an expansion of the previous stage and, therefore, the growth direction (chain of more molecules) and the interactions remain the same, with the only difference being the value of the stabilization energy, which, in order to compete, is doubled and, therefore, will be -37.44 kcal mol$^{-1}$, in addition to the total value of the area, which is 157.28 Å$^2$. When comparing the energies of each hypothesis presented, it is clear that hypothesis IV is thermodynamically favored and, therefore, the chain formed by this hypothesis is the structure resulting from stage 3 and it is from this structure that the growth of the next stage occurs (**Figure S15**).

Stage 4 has four hypotheses similar to those in the previous stage, where, again, there is only a distinction in the number of molecules present in each hypothesis. In the hypothesis I, growth along the *b*-axis suggests a new expansion; thus, the contact points and the growth axis are the same. Reinforcing this, the stabilization energy is -37.44 kcal mol$^{-1}$ and the total area is 157.28 Å$^2$, as presented in the previous stage. Hypothesis II suggests a growth in the direction of the *c* axis from the Br···Br type interactions, which form an area of 45.60 Å$^2$ and result in an energy of -9.47 kcal·mol$^{-1}$. Hypothesis III reveals an area of 162.52 Å$^2$ formed by the C-H···Br, C···Br and C-H···C contact points oriented along the *c* axis, resulting in an energy of -31.79 kcal·mol$^{-1}$. In the last hypothesis of this stage, growth occurs in the direction of the *a axis*, guided by C-H···O and C-H···Br interactions that present a contact area of 278.31 Å$^2$ and a stabilization energy of -44.64 kcal·mol$^{-1}$, forming a supramolecular layer (2D). Since hypothesis IV presents the highest stabilizing energy, it should guide stage 4 of the proposed crystallization mechanism of (*E*)-*o*-Br (**Figure S15**).

In stage 5, only three distinct hypotheses emerge. The first hypothesis is an expansion of the previous stage, that is, the same contact points and the same growth axis; however, the stabilization energy, for competition purposes, is doubled, resulting in a value of -89.28 kcal mol$^{-1}$ and a total area of 556.62 Å$^2$. In the second hypothesis, growth along the *b* axis is suggested, in which the contact area formed is 300.68 Å$^2$ and the energy contribution is -65.57 kcal mol$^{-1}$ due to π···π interactions (where the contact points are C···C, Br···Br, C···Br, and C···N). In the last hypothesis of this stage, hypothesis III, growth occurs in the direction of the *c* axis, guided by the contact points Br···Br, C-H···Br, C···Br, and C-H···C with an energy equal to -114.30 kcal·mol$^{-1}$ and a contact area of 578.75 Å$^2$ (**Figure S15**). When comparing the stabilizing energies of this stage, it is noted that the hypothesis with the highest stabilizing energy is hypothesis III. Therefore, after the occurrence of this hypothesis, the formed

structure already has three spatial dimensions and all the interactions present in the first coordination sphere occurred during the proposed mechanism, for the oxime *(E)*-*o*-Br.

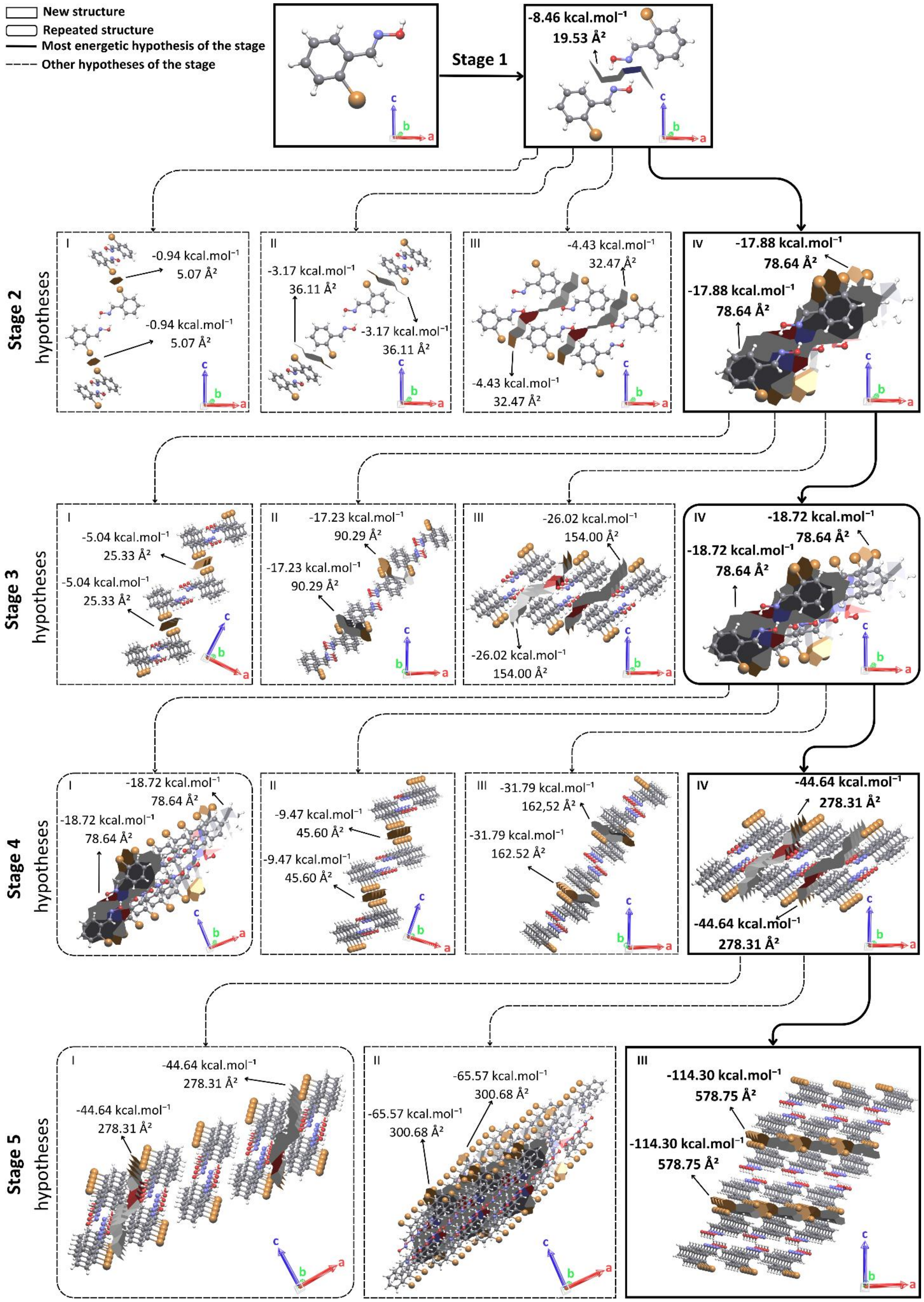


**Figure S15.** Crystallization mechanism proposal for *(E)-o*-Br.

When starting the proposed crystallization mechanism of *(E)-m*-Cl, the occurrence of π stacking was observed (an interaction that also occurs in the first growth stage in our previous study on phenols,[43] anilines,[45] and alcohols,[46] all halogenated). The stacking is in the direction of the *b* axis, in which each approach has an energy of -6.31 kcal $mol^{-1}$. Therefore, in the first stage, a chain of at least three molecules is formed, in which each approach results in a contact area of 31.59 $Å^2$ (**Figure S16**).

In stage 2, several hypotheses were observed, but only the hypotheses with higher energy than the previous stage were considered in the representation of **Figure S16**. In hypothesis I of stage 2 there is an expansion of the previous stage, forming a longer chain and, therefore, the energy for competition purposes is -12.62 kcal $mol^{-1}$ with a total contact area of 63.18 $Å^2$. Hypothesis II suggests growth along the *c* axis, driven by C-H···Cl interactions that contribute to a stabilization energy of -8.42 kcal·$mol^{-1}$, with a contact area of 53.85 $Å^2$ in each approximation. Hypothesis III suggests an approximation of two-chain, forming a dimeric structure. The supramolecular dimer has a contact area of 88.60 $Å^2$ and its energy is -16.04 kcal·$mol^{-1}$ due to C-H···C, C-H···Cl, and C···Cl interactions. Hypothesis IV points to a supramolecular chain approximation based on C-H···O and C-H···H-C interactions. The resulting energy is -20.13 kcal·$mol^{-1}$ and the contact area is 93.32 $Å^2$. Hypothesis V presents C-H···O and O-H···N contact points that result in an energy of -26.29 kcal·$mol^{-1}$ with a contact area of 67.09 $Å^2$. Hypothesis V should guide the growth of stage 2 with the formation of a supramolecular dimer (**Figure S16**), as it presents a higher stabilizing energy.

In stage 3 there are four distinct hypotheses. Hypothesis I of stage 3 suggests growth along the *a* axis, with the only interaction present being the halogen bond Cl···Cl via σ-hole, which has an energy of -1.00 kcal·$mol^{-1}$ and an area of 13.79 $Å^2$. Hypothesis II also suggests growth in the *a*-axis direction, with a contact area of 88.60 $Å^2$ formed by the C-H···C, C-H···Cl, and C···Cl contact points, resulting in an energy of -16.57 kcal·$mol^{-1}$. Hypothesis III points for a new π-stacking, in addition to the O-H···N and C-H···O interactions. This approximation results in an energy of -18.72 kcal·$mol^{-1}$ and an area of 76.59 $Å^2$. In hypothesis IV, growth should occur along the *c* axis, driven by C–H···Cl, C–H···O, and C–H···H–C interactions, with a stabilization energy of −36.13 kcal·$mol^{-1}$ and a contact area of 182.63 $Å^2$, resulting in a supramolecular layer. Therefore, this hypothesis should guide the growth of stage 3 (**Figure S16**).

In stage 4, there are only three different hypotheses. Hypothesis I suggests growth along the *a* axis from C···Cl, C-H···C, C-H···Cl, and Cl···Cl interactions (interaction via the σ-hole, also present in our previous studies with carboxylic acids,[42] phenols,[43] and anilines,[45] all halogenated aryl) that result in an energy value of -51.87 kcal·$mol^{-1}$ and form a contact area of 290.23 $Å^2$. Hypothesis II reveals a π-stacking formed by the O-H···N, C-H···O, C-H···Cl and C-H···H-C contacts, with an area equal to 304.76 $Å^2$, which grows along the *b* axis with an energy of -69.60 kcal·$mol^{-1}$. In the hypothesis III, an expansion of the previous stage should occur, which only increases the supramolecular layer already formed. Growth occurs from the same interactions (C-H···Cl, C-H···O, and C-H···H-C) with an area of 365.26 $Å^2$ and stabilizing energy of -72.26 kcal·$mol^{-1}$ (energy doubled because it is an expansion stage). Therefore, hypothesis III should guide the growth of stage 4 in the crystallization mechanism (**Figure S16**).

In stage 5, three distinct hypotheses were observed. The hypothesis I is an expansion of the previous stage, and therefore, growth proceeds in the direction of the *c*-axis, and the interactions, contribution energy, and contact area remain the same as in hypothesis III of stage 4. Hypothesis II of stage 5 proposes growth along the *a*-axis, based on interactions of the C···Cl, C-H···C, C-H···Cl, and Cl···Cl types, which, when added together, result in an energy of -87.69 kcal·$mol^{-1}$, resulting in a contact area equal to 491.87 $Å^2$. In hypothesis III a new π-stacking should occur, growing along the *b* axis, in which the observed contact points are O-H···N, C-H···O, C-H···Cl and C-H···H-C. The stabilization energy and contact area found were -121.60 kcal·$mol^{-1}$ and 532.93 $Å^2$, respectively. Thus, the growth of stage 5 should be guided by hypothesis III (**Figure S16**).

In stage 6, three different hypotheses were calculated. Hypothesis I exhibits a π-stacking along the *b*-axis, presenting the same interactions as hypothesis III from the previous stage, characterizing an expansion. Thus, the stabilization energy is -243.2 kcal $mol^{-1}$ and the total area is 532.93 $Å^2$. Hypothesis

II suggests growth along the *c*-axis, based on interactions of the C-H···Cl, C-H···O, and C-H···H-C types. These interactions, when added together, result in a energy of -123.48 kcal $mol^{-1}$ and form a contact surface equal to 642.05 $Å^2$. Finally, in hypothesis III, it indicates a growth in the direction of the *a axis*, according to the presence of the C···Cl, C-H···C, C-H···Cl and Cl···Cl interactions, in which the sum of these interactions results in a stabilizing energy of -310.60 kcal·$mol^{-1}$ and an area of 1701.71 $Å^2$. Stage 6 should have its growth guided by hypothesis III, forming a three-dimensional structure from the occurrence of all interactions present in the cluster. Thus, the proposed crystallization mechanism of the oxime *(E)-m*-Cl is completed in 6 stages (**Figure S16**).

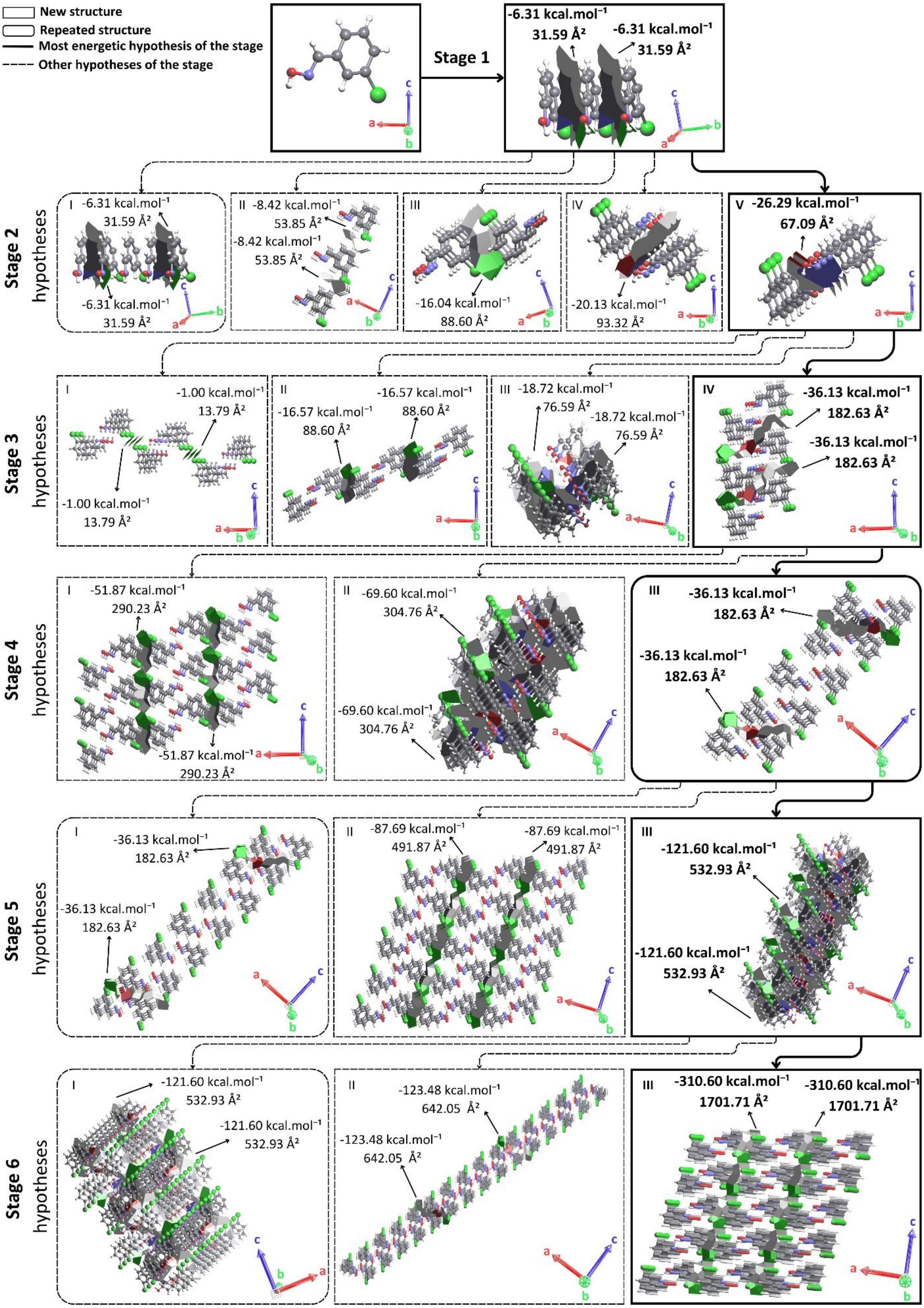


**Figure S16.** Crystallization mechanism proposal for *(E)-m*-Cl.

When starting the proposed crystallization mechanism for compound *(E)-p*-F, it is observed that the most stabilizing energy present in the first coordination sphere is that of the O-H···N interaction. This interaction has an energy of -13.00 kcal·mol$^{-1}$ and occupies a contact area of 18.93 Å$^2$. Because this is the most favorable stabilization energy, the first stage of the proposed mechanism begins with the formation of this dimer, as illustrated in **Figure S17**, and it is from this dimer that stage 2 proceeds.

In stage 2, four distinct hypotheses are suggested, in which hypothesis I reveals interactions of the C-H···F and F···F type, which represent an energy of -1.67 kcal·mol$^{-1}$, with an area of 10.53 Å$^2$ and a growth between the *a* and *c* axes. Hypothesis II reveals a growth in the *a* axis direction from the C···F and C-H···F interactions. The contact area is 10.82 Å$^2$ with a stabilization energy of -1.74 kcal·mol$^{-1}$. In hypothesis III, growth along the *c* axis is observed, guided by interactions of the C-H···O, C-H···H-C, C-H···C and C···O types. This interaction contributes an energy of -7.29 kcal·mol$^{-1}$ and forms a contact area of 37.98 Å$^2$. In hypothesis IV, the contact area is 61.31 Å$^2$ with a stabilization energy of -12.09 kcal·mol$^{-1}$, forming a π···π stacking that occurs along the *b* axis, and results in the formation of a supramolecular chain. Because it has the most stabilizing energy, hypothesis IV, in which a supramolecular chain is formed, presents the structure formed at the end of stage 2(**Figure S17**).

In stage 3, four distinct hypotheses are also calculated. Hypothesis I is an expansion of the previous stage, doubling the energy of the hypothesis, resulting in -25.05 kcal mol$^{-1}$ with a total area of 122.62 Å$^2$. Hypothesis II suggests growth along the *a*-axis, revealing an area of 35.95 Å$^2$ due to the C-H···F and F···F interactions, which, when added, yield an energy of -6.42 kcal mol$^{-1}$. Hypothesis III proposes a stabilizing energy of -9.26 kcal mol$^{-1}$, with growth along the *a*-axis, yielding an area of 54.09 Å$^2$ from the C···F and C-H···F interactions. In hypothesis IV of the third stage, growth occurs along the *c* axis, guided by interactions of the C-H···O, C-H···H-C and C-H···C and C···O types, which form a supramolecular layer with a contact area equal to 189.88 Å$^2$, with a stabilization energy of -39.63 kcal·mol$^{-1}$ (**Figure S17**). Because it is the energetically most favorable hypothesis, hypothesis IV is the one that should guide stage 3 and this formed supramolecular layer should initiate the next growth.

In stage 4, only three distinct hypotheses are investigated. Hypothesis I present an energy of -38.57 kcal·mol$^{-1}$, with a contact area of 234.18 Å$^2$. The interactions of this hypothesis are of the C···F, C-H···F, and F···F types and present growth along the *a* axis. Hypothesis II suggests a stacking by π···π interactions that grow in the direction of the *b* axis, revealing an area of 259.89 Å$^2$ and an energy of -53.25 kcal·mol$^{-1}$. The hypothesis III is an expansion of the previous stage and, therefore, the growth axis and interactions remain the same, which elongates the already formed layer. The energy considered for competition purposes has its value doubled, resulting in -79.26 kcal mol$^{-1}$, presenting a total area of 379.76 Å$^2$. Thus, because it presents the greatest amount of stabilizing interaction energy, hypothesis III should guide step 4 of the proposed crystallization mechanism of benzoxime (E)-p-F (**Figure S17**).

In stage 5, three distinct hypotheses emerge again. Hypothesis I reveals an expansion of the previous stage that has the same data as hypothesis III of the fourth stage. In hypothesis II, the suggested growth is in the direction of the *a* axis, from interactions of the types C···F, C-H···F, and F···F, which have a combined energy of -68.34 kcal·mol$^{-1}$ with a contact area of 414.28 Å$^2$. In hypothesis III a new stacking occurs along the *b* axis, driven by π···π interactions, resulting in an area of 458.47 Å$^2$ and a stabilization energy of -94.83 kcal mol$^{-1}$ (**Figure S17**). When comparing the energies presented in this stage, hypothesis III has the highest stabilization energy, which should lead to stage 5 of the mechanistic proposal.

In stage 6, there are, once again, only three hypotheses to be checked. In hypothesis I, the suggested approximation is a stacking that grows along the *b* axis, with the same interactions as hypothesis III of the fifth stage. Therefore, it is an expansion, resulting in an energy of -189.66 kcal mol$^{-1}$ and a total area of 916.94 Å$^2$. Hypothesis II exhibits growth along the *c* axis, oriented by interactions of the C-H···O, C-H···H-C, and C-H···C and C···O types, which result in an energy of -133.39 kcal mol$^{-1}$ and form an area of 645.58 Å$^2$. In hypothesis III, there is growth in the direction of the *a* axis, forming an area of 1368.47 Å$^2$, which represents a stabilization energy of -221.41 kcal·mol$^{-1}$ due to the C···F, C-H···F and F···F interactions. Hypothesis III is thermodynamically favored and, after its

occurrence, the formed structure already has three dimensions and presents all the interactions of the first coordination sphere. Therefore, the proposed crystallization mechanism of compound *(E)*-*p*-F ends with six stages (**Figure S17**).

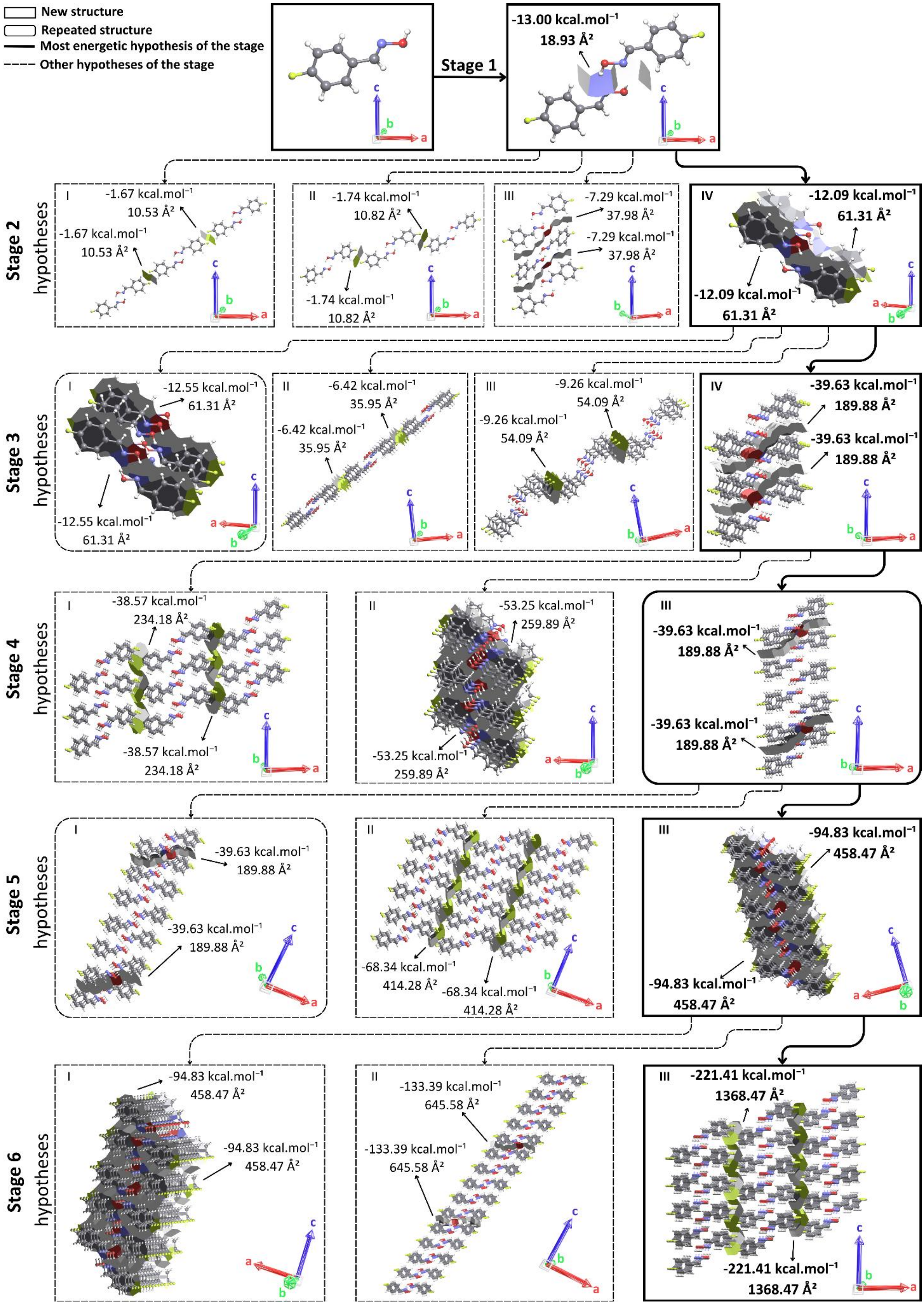


**Figure S17.** Crystallization mechanism proposal for *(E)-p*-F.

Benzoxime *(E)-p*-Br is isostructural to *(E)-p*-Cl; therefore, only the most energetic hypotheses of each stage will be addressed. The proposal begins with stage 1, where the O-H···N dimer forms, resulting in a contact area of 22.37 Å² and a stabilization energy of -9.62 kcal·mol⁻¹ (**Figure S18**).

In stage 2, dimers should stack, forming a supramolecular chain, growing along the *b* axis from the C····O, C···N, C···C, and C···Br interactions, representing -16.84 kcal·mol⁻¹ of stabilizing energy and generating an area of 70.51 Å² (**Figure S18**).

In stage 3, the approximation along the a axis results in a contact area and a stabilization energy equal to 205.26 Å² and -34.20 kcal.mol$^{-1}$, respectively, due to the C-H···Br, C-H···O and C-H···H-C interactions (**Figure S18**).

In stage 4 there was an expansion of the previous step, changing only the energy value: -68.40 kcal.mol-1 (**Figure S18**).

Stage 5 must have its growth guided by π stacking, along the b axis, which results in a contact area of 523.16 Å² and a stabilization energy of -113.43 kcal.mol$^{-1}$ (**Figure S18**).

In stage 6, growth should occur along the c axis, resulting in a stabilization energy of -332.23 kcal.mol-1 and a contact area equal to 1651.69 Å², due to C-H···Br and Br···Br interactions. This last growth results in a three-dimensional structure that presents all the interactions of the first coordination sphere, concluding the proposed crystallization mechanism of *(E)-p*-Br (**Figure S18**).

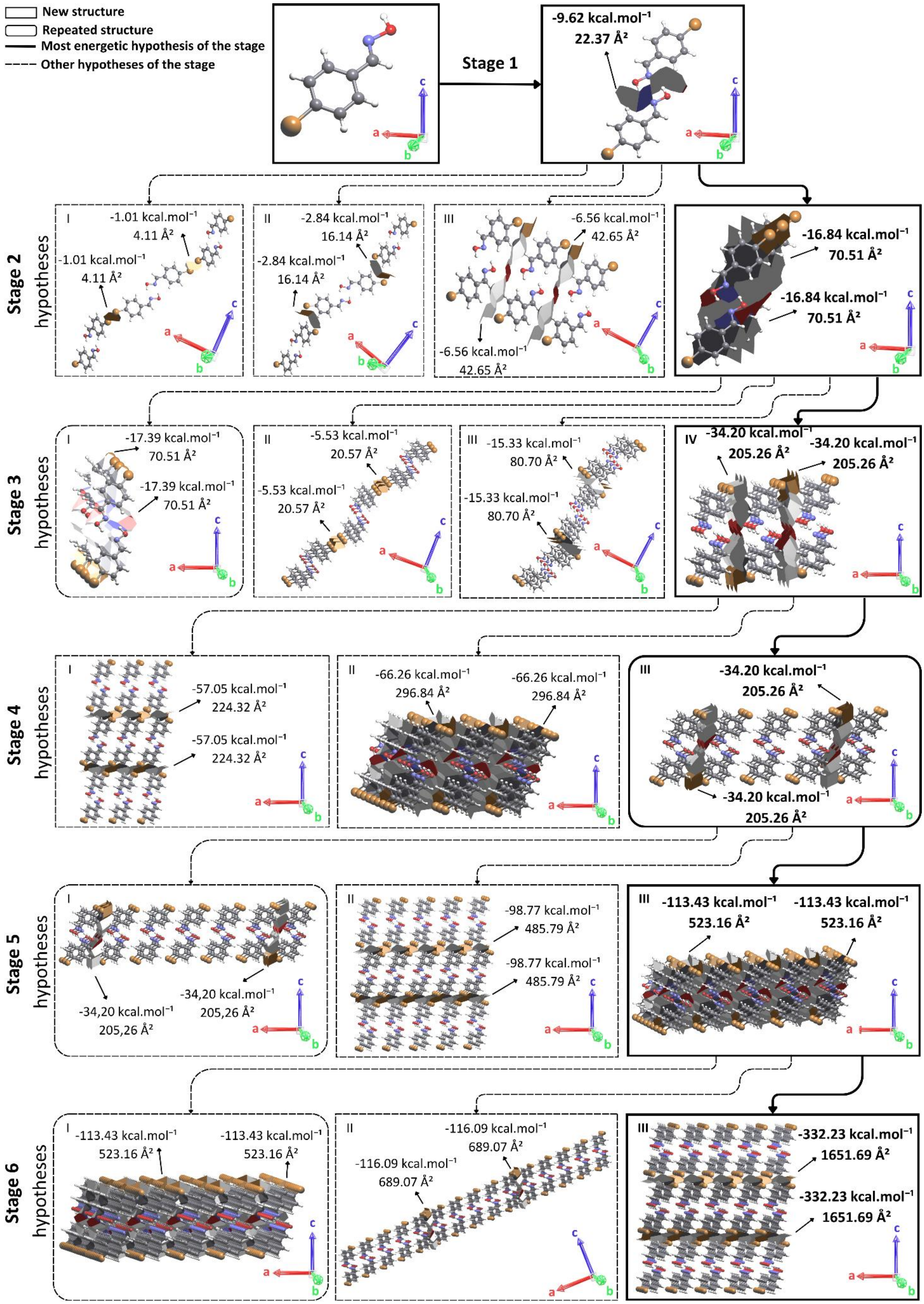


**Figure S18.** Crystallization mechanism proposal for *(E)*-*p*-Br.

Like the pair *(E)-p*-Cl/*(E)-p*-Br, the pair *(Z)-p*-Cl/*(Z)-p*-Br was also found to be isostructural. Therefore, again only the energetically favored hypotheses will be presented. Stage 1 should occur by a π stacking, along the *c* axis, with a contact area of 28.07 Å² and an energy of -6.32 kcal·mol⁻¹, referring to the contact of the dimers M1···M9 and M1···M13 of the first coordination sphere (**Figure S19**). The most energetic hypothesis in stage 2 is hypothesis V, which presents a dimeric approximation, by hydrogen bonds (O-H···N), forming a supramolecular chain with an area of 38.65 Å² and an energy of -23.28 kcal·mol⁻¹ (**Figure S19**). In stage 3, growth occurs along the *a* axis, guided mainly by C-H···O, C-H···N, C···N, and C····O interactions, which presents an area of 220.47 Å² and an energy of -46.91 kcal·mol⁻¹, resulting in the formation of a supramolecular layer (two-dimensional structure, as shown in **Figure S19**). An expansion of the previous stage occurs in stage 4, doubling the energy to -93.82 kcal mol⁻¹ and the area to 440.94 Å, with the same interactions, only stretching the already formed supramolecular layer (**Figure S19**). π-stacking occurs in stage 5 along the *c* axis, which has an energy of -126.96 kcal mol⁻¹ and a contact area of 488.49 Å², forming a three-dimensional structure. However, since not all interactions present in the first coordination sphere have been described so far, the mechanism proceeds to stage 6 (**Figure S19**). In stage 6, growth occurs along the a and b axes, resulting in a contact area of 1650.43 Å² and a stabilization energy of -313.60 kcal·mol⁻¹. This growth is guided by C-H···Br interactions. After stage 6, the formed structure presents three-dimensional growth (mesocrystal) and all interactions present in the first coordination sphere are represented in the proposed mechanism of the compound *(Z)-p*-Br (**Figure S19**).

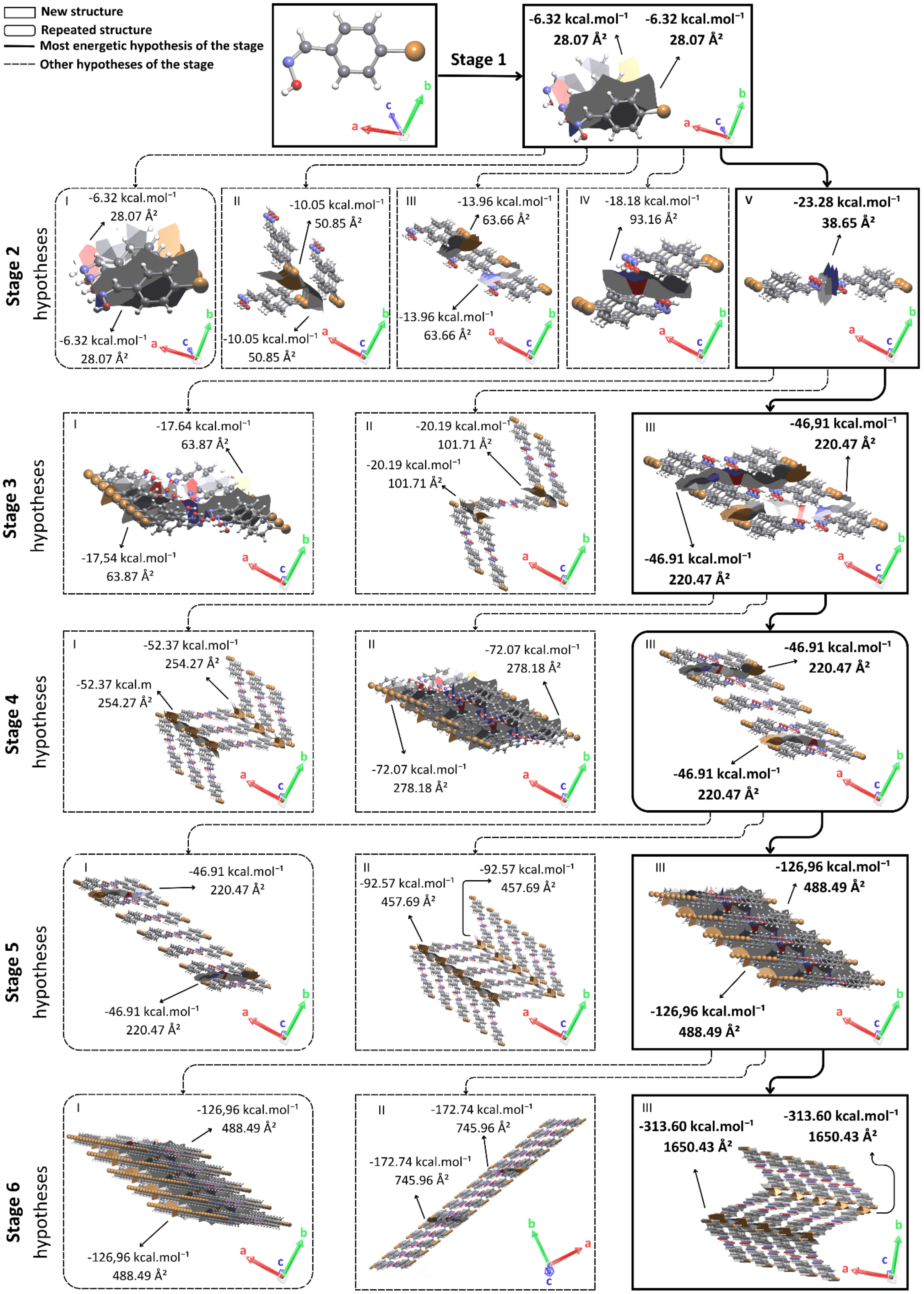


**Figure S19.** Crystallization mechanism proposal for *(Z)-p*-Br.

When starting the proposal of the crystallization mechanism of (*E*)-*p*-I, it is observed that the most stabilizing energy of the first coordination sphere comes from the O-H···N interaction, which has a value of -7.67 kcal·mol⁻¹. The contact area related to this interaction is 20.58 Å², in which a supramolecular dimer forms in the first stage 1 (**Figure 20**).

In stage 2, there are four distinct hypotheses. Hypothesis I suggests growth along the *a* axis, with C-H···H-C and C-H···I interactions that, when added, result in an energy of -2.93 kcal·mol⁻¹ with a contact area of 18.19 Å². In Hypothesis II, the interactions C-H···H-C, C-H···I, and C-H···O are observed. These interactions have an energy of -5.00 kcal·mol⁻¹ and form an area of 36.81 Å² along the *c* axis. Hypothesis III suggests growth between the a and c axes, driven by interactions of the O···I, C-H···I and C-H···H-C types, which have an area of 29.89 Å² and an energy contribution of -6.56 kcal·mol⁻¹. Hypothesis IV points to a growth between the a and b axes forming a supramolecular chain from interactions such as O···I, C···N, C-H···N and C-H···I, which, when added, result in an energy of -13.59 kcal·mol⁻¹ with an area of 65.92 Å² (**Figure S20**). Because it has the highest stabilizing energy, hypothesis IV should guide stage 2 of the mechanistic proposal for (*E*)-*p*-I.

In stage 3, there are only three different hypotheses. Hypothesis I is an expansion of the previous stage; therefore, the contact points and growth axis remain the same, while the stabilization energy value, for the sake of comparison is doubled, resulting in -28.46 kcal mol⁻¹, giving a total area of 131.84 Å². Hypothesis II suggests growth along the b axis, with a stabilization energy of -25.85 kcal·mol⁻¹ due to the C-H···I and C-H···H-C interactions, which form a contact area of 141.24 Å². Hypothesis III indicates growth along the a axis with interactions of the C-H···I, O···I and C-H···H-C type, which represent an energy of -32.18 kcal·mol⁻¹ and an area of 163.43 Å². At the end of stage 3, the most stabilizing energy is that of hypothesis III, forming a supramolecular layer, and it is from this formed layer that the growth of the next stage will occur (**Figure S20**).

Stage 4 of the proposed (*E*)-*p*-I crystallization mechanism reveals three hypotheses to be investigated. Hypothesis I is an expansion of the previous stage and, thus, has a total contact area of 326.86 Å² and a stabilization energy of -64.36 kcal mol⁻¹, which are double the values of hypothesis I from Stage 3. Hypothesis II suggests a stacking along the *a* axis, formed from the interactions C-H···O, C-H···I, C-H···H-C, and C-H···N, which represents a contact area of 271.55 Å² and a stabilization energy of -52.93 kcal mol⁻¹. In hypothesis III the contact area formed by the O···I, C···N, C-H···N, and C-H···I interactions presents a value of 525.98 Å² with a stabilizing energy of -100.40 kcal·mol⁻¹. Hypothesis III has the most stabilizing energy, and after its occurrence, a three-dimensional structure should be formed from all the interactions present in the first coordination sphere. Therefore, the proposed crystallization mechanism of the oxime *(E)*-*p*-I is concluded in the fourth stage (**Figure S20**).

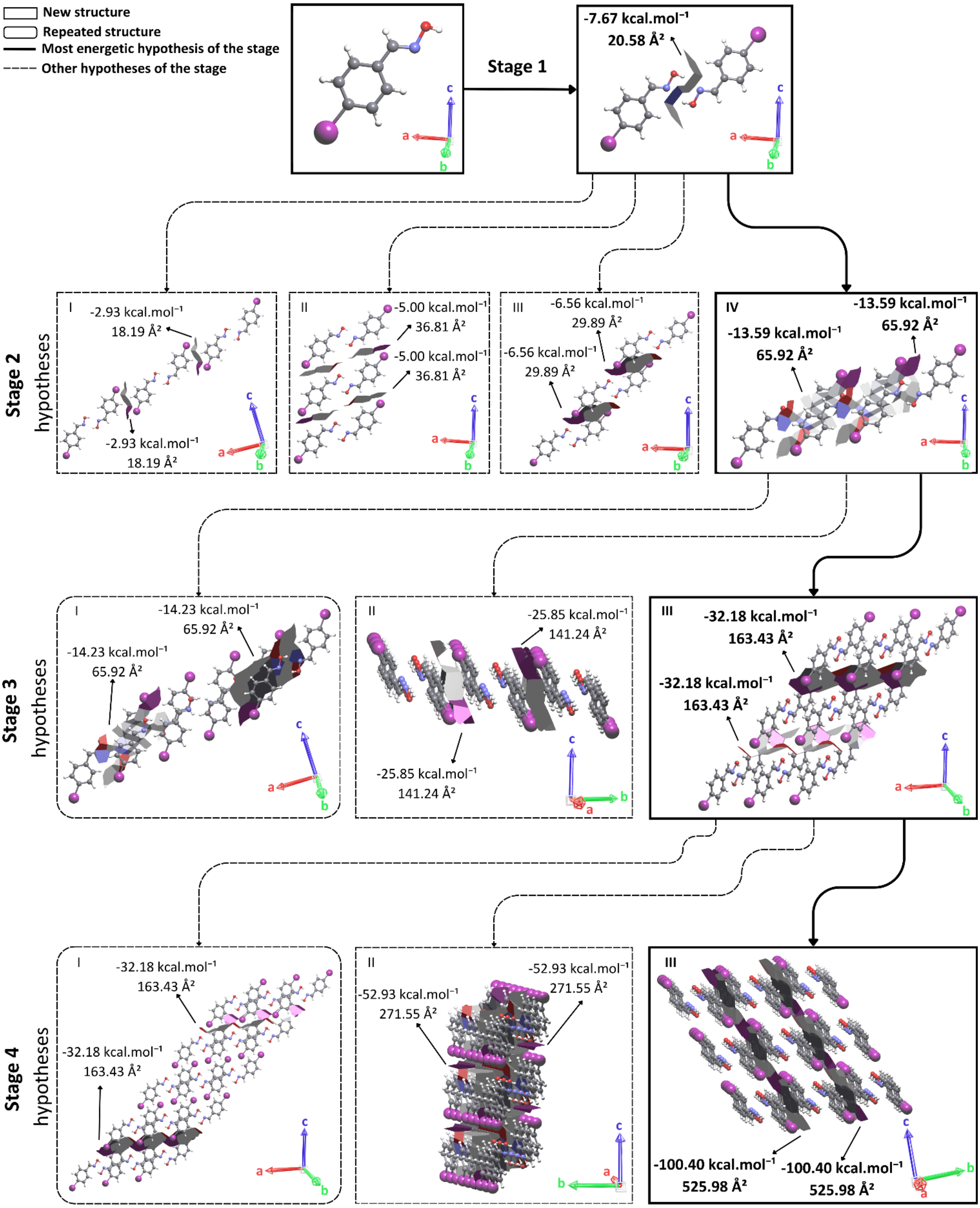


**Figure S20.** Crystallization mechanism proposal for *(E)*-*p*-I.
